# Supplementary material for: Intraoperative margin assessment for basal cell carcinoma with deep learning and histologic tumor mapping to surgical site
Source: NPJ Precis Oncol. 2024 Jan 3;8:2. doi: 10.1038/s41698-023-00477-7 (PMC10764333; doi:10.1038/s41698-023-00477-7)
Supplement: Supplementary file 1 — Supplementary Information File [file 41698_2023_477_MOESM1_ESM.pdf]

Supplemental Information

Supplementary Figures

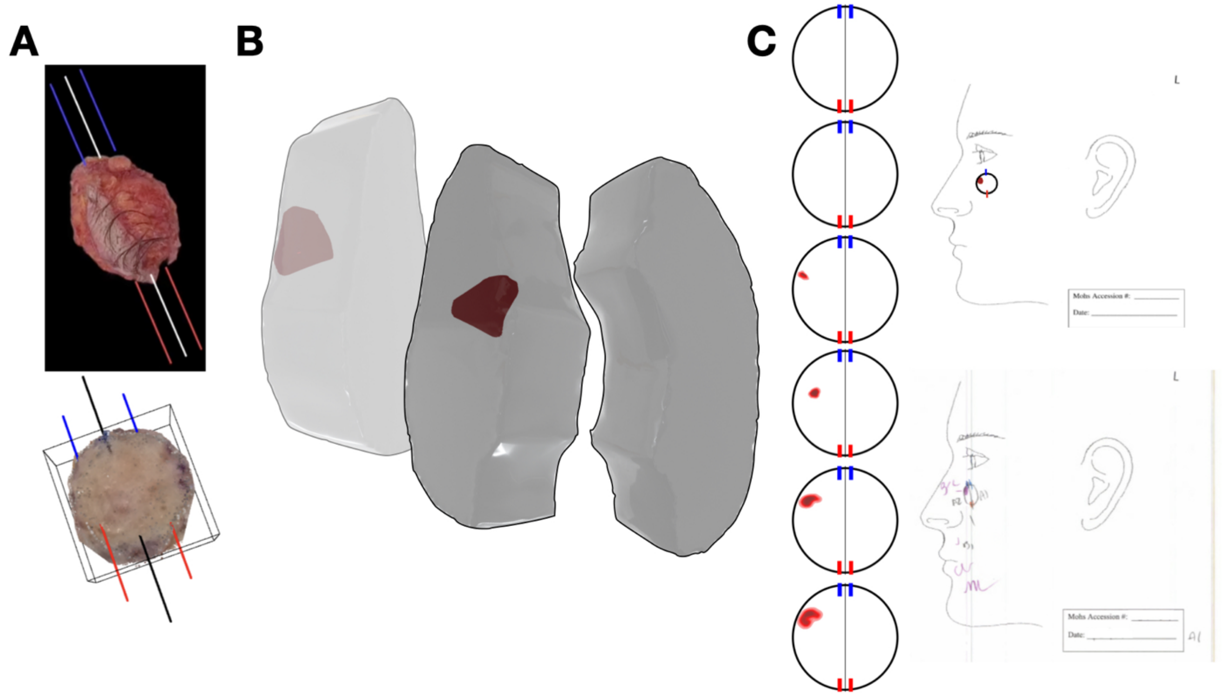

Supplementary Figure 1: Comparison of three different forms of 3D modeling in pathology: A) gross tissue specimen modeling prior to tissue grossing, sectioning, staining and scanning; B) 3D histopathology visualized using 3D web application, where image registration algorithms are used to align serial sections of tissue separated by 5-micron depth to each other, segmenting histological results at each layer (positive margin in red); C) surgical tumor mapping, where histological results (red heatmap) at each section are mapped to a standard 2D coordinate system using inks (blue is 12 o'clock, red is 6 o'clock) to guide standard placement of the histological results, using inks to also orient these results to the original anatomic position/orientation; methods A and C are the focus of this study as precise 3D histopathology is too time intensive for intraoperative assessment and does not focus on anatomic orientation using inks; 3D models and surgical maps from B) and C) were generated for the same patient (example patient 1)

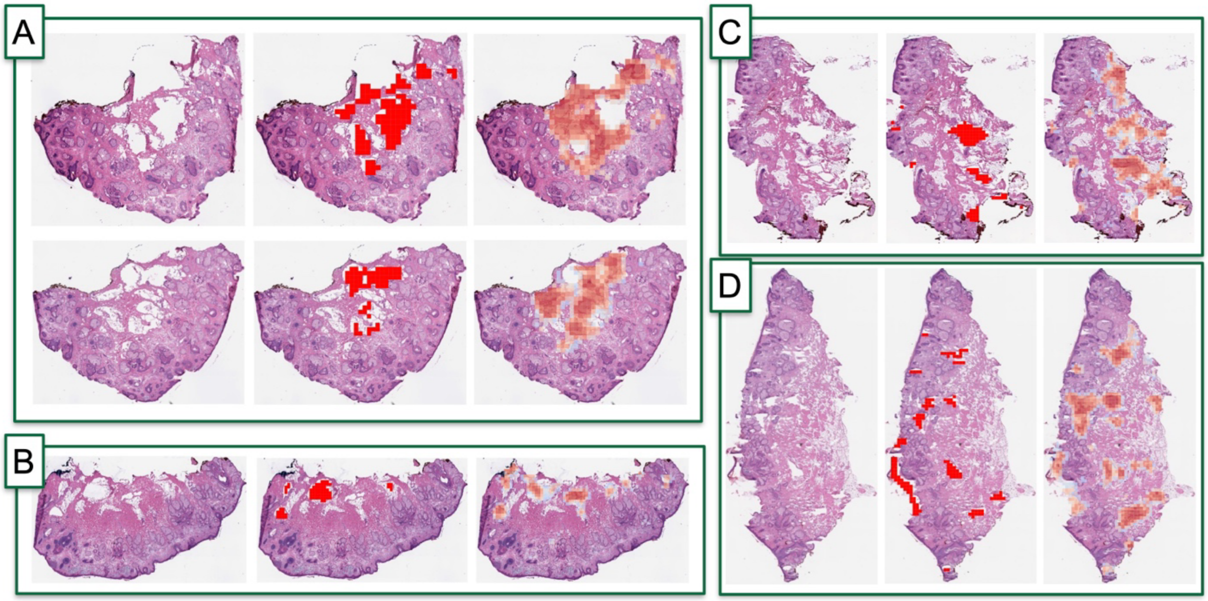

Supplementary Figure 2: Examples of holes and tears missed/overcalled: A-D) represent sections randomly selected from four separate cases; left image is original WSI; middle image annotated holes/tears from the pathologist are denoted in red; right image overlaid is a heatmap where degree of red for an image subarray indicates predicted probability of incompleteness by Completeness GNN

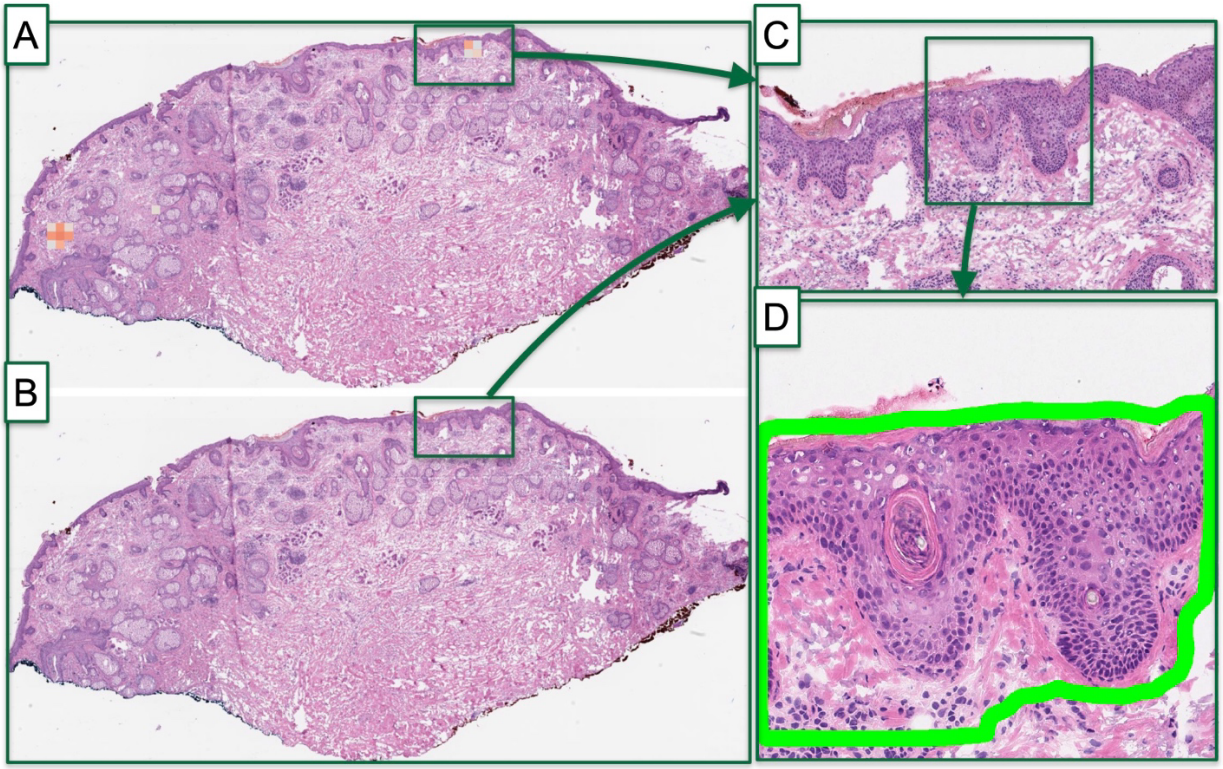

Supplementary Figure 3: Example of follicle prediction impact on half of one tissue section: A) heatmap is overlaid on WSI to denote predicted tumor probability (red) by Tumor GNN; B) tumor probability heatmap after accounting for presence of follicles as predicted by the follicle detection neural network; note how in this slide tumor is no longer predicted, which results in an increased area under the curve of 0.05 across the WSI for the case; C) zooming in on one tumor-predicted region called follicle by the follicle detection algorithm; D) zooming in on specific subarray where follicle was predicted by follicle detection network, where predicted follicular structure is outlined in green by the neural network

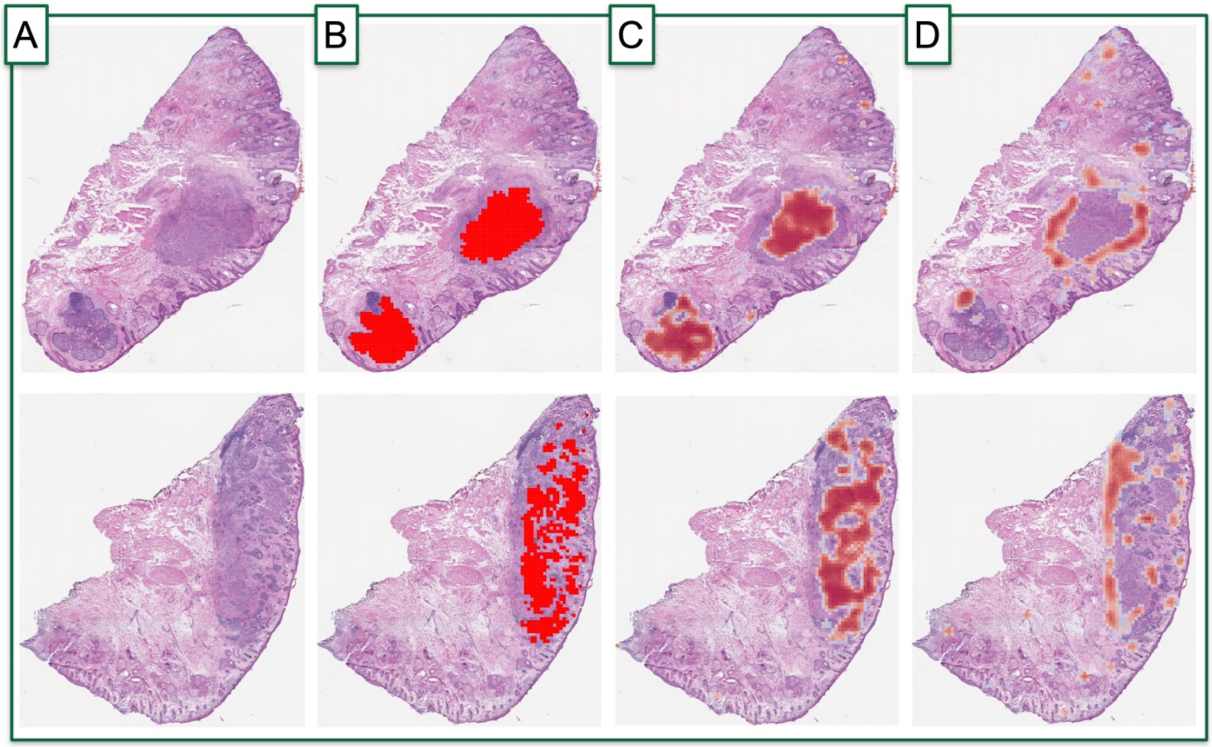

Supplementary Figure 4: Prediction of inflammation on two separate tissue sections to illustrate importance of taking inflammation into account for tumor prediction: A) original slide image; B) ground truth / annotated tumor denoted in solid red regions; C) predicted tumor regions given by heatmap where degree of red reflects tumor probability via Tumor GNN; D) predicted inflammatory regions given by heatmap where degree of red reflects inflammatory probability via Tumor GNN

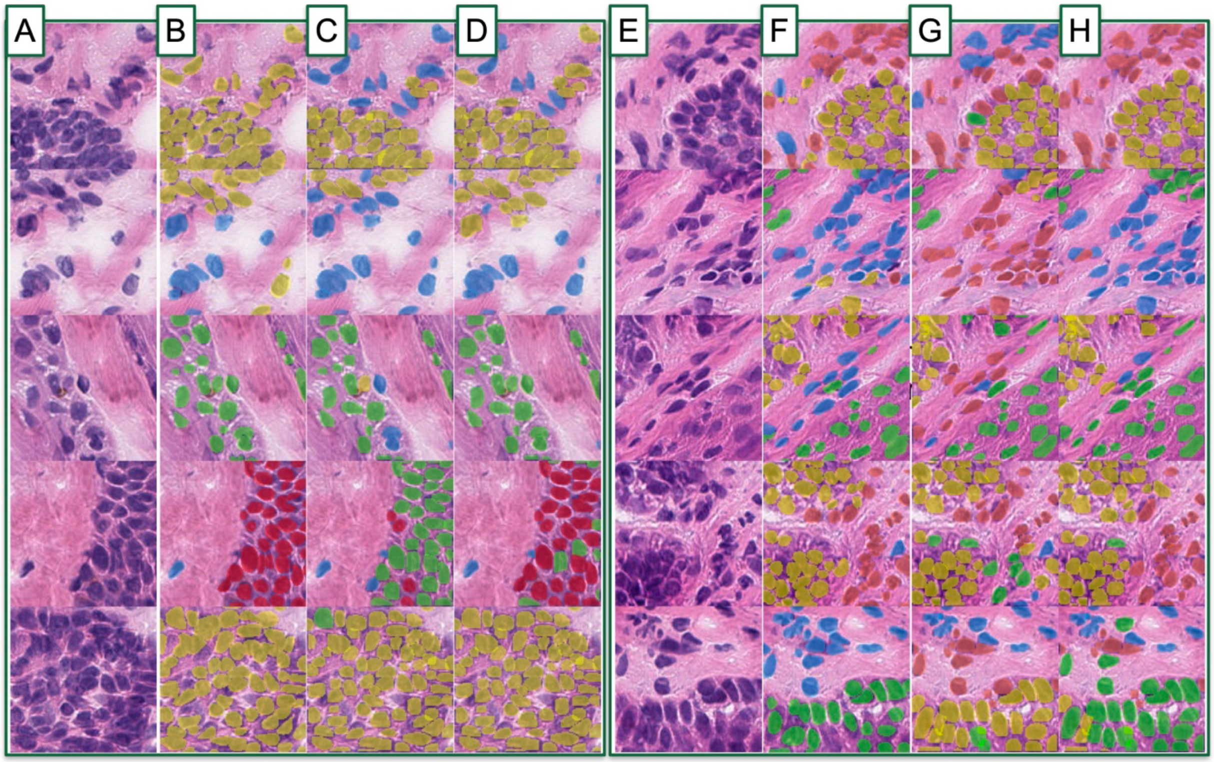

Supplementary Figure 5: Example outputs from nuclei prediction workflow to delineate BCC (yellow), hair follicle (green), inflammatory (orange), fibroblast (blue), and epidermal keratinocyte cells (red): A,E) original subimages; B,F) overlaid annotated cells and cell assignments; C,G) Detectron2 predicted cell assignments; D,H) Cell graph neural network predicted cell assignments

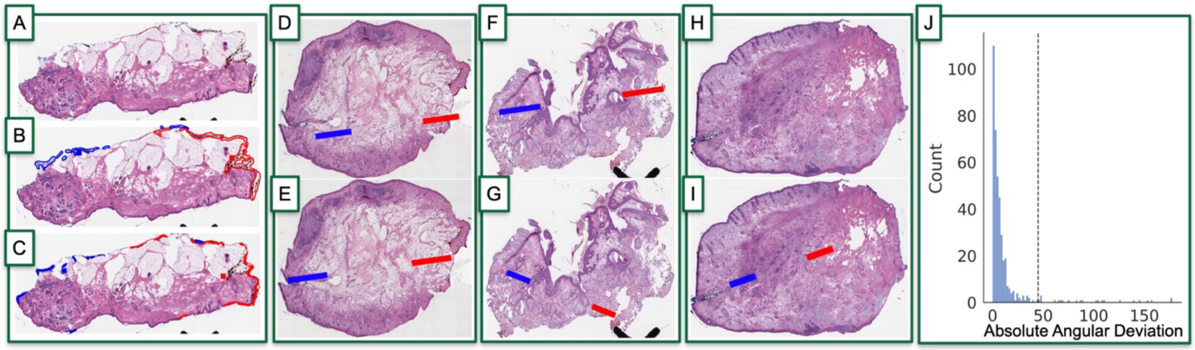

Supplementary Figure 6: Tissue orientation compared to ground truth: A) Original slide image; B) pathologist annotation of red and blue inks given by outlined splines; red and blue squares denote center of mass for red and blue inks respectively; C) ink detector's prediction of red/blue ink and ink centers of mass; D-E) example of optimal ink detection and orientation; D) tissue orientation (i.e., line between red and blue inks) as inferred from pathologist's annotations; E) tissue orientation (i.e., line between red and blue inks) as predicted by ink orientation algorithm; F,G) example of suboptimal ink detection and orientation due to sectioning quality; with same comparison from D-E); H,I) example of suboptimal ink detection and orientation due to missing red ink on right side; while pathologist was unable to annotate H), the algorithm still managed to report orientation for I); J) histogram denoting angular difference between pathologist-annotated orientation and algorithm-predicted orientation, in degrees, where each element reflects tissue section

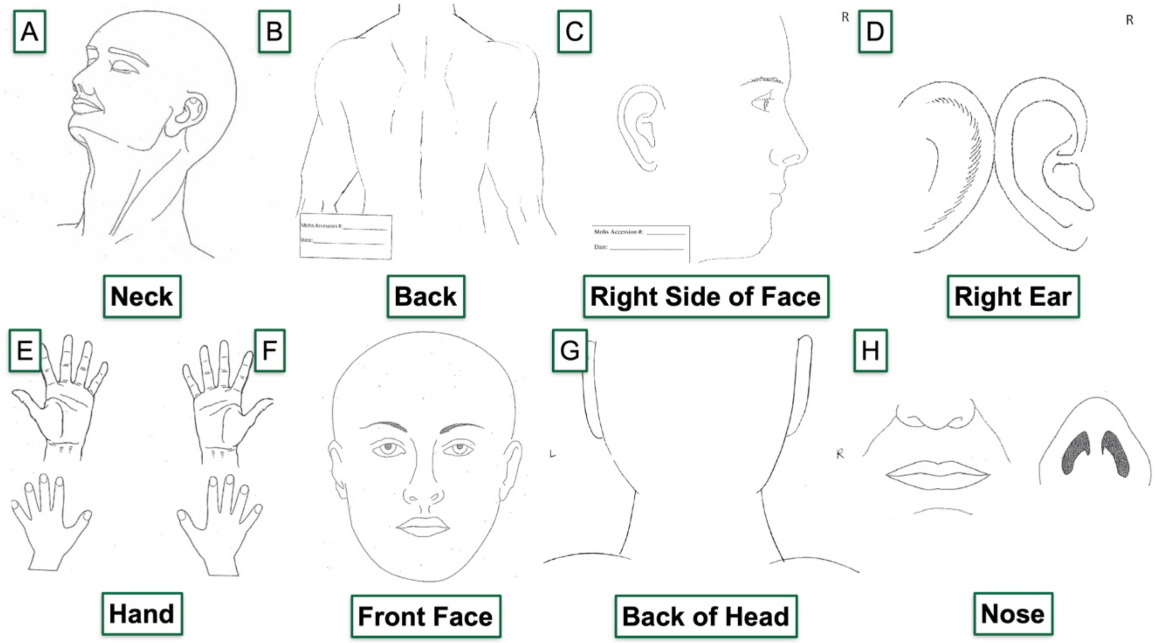

Supplementary Figure 7: Example templates of anatomic locations for surgical tumor mapping: A-F) examples of different anatomical locations that can be selected by the user for real-time mapping

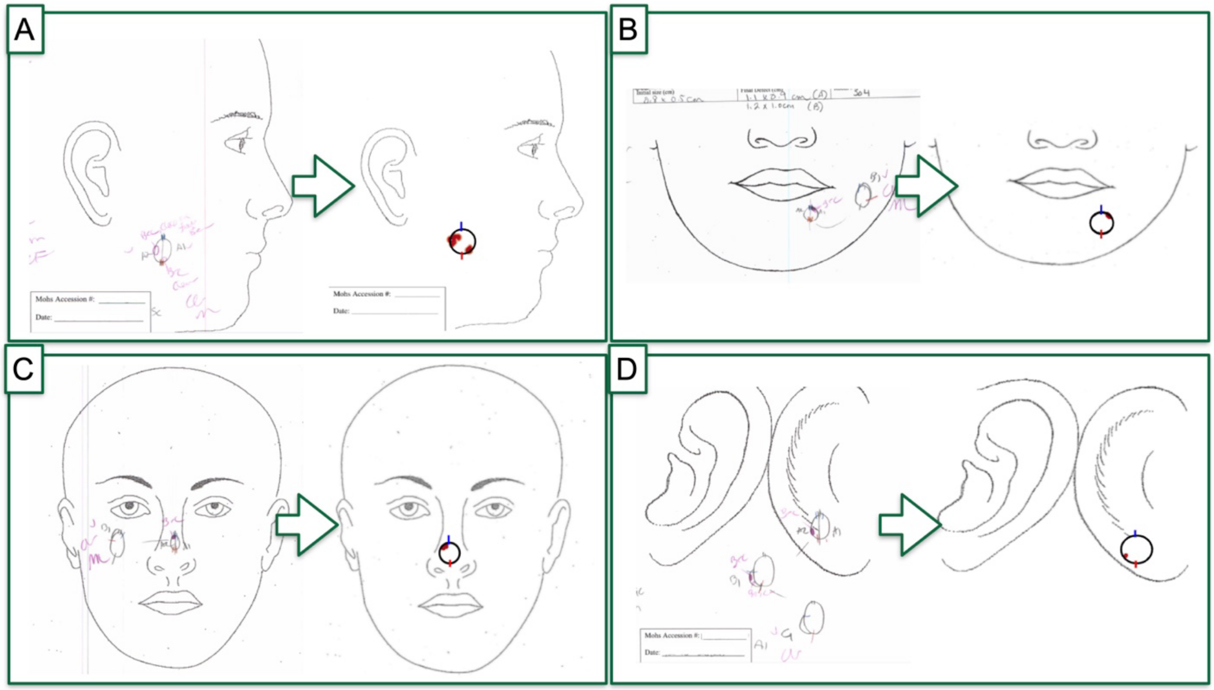

Supplementary Figure 8: Comparison of hand-drawn surgical tumor maps (left) to algorithm predicted tumor maps (right): A-D) four separate cases, predictions from first site predictions compared to ground truth; subsequent resection stages were removed from these images to draw attention to the initial resection

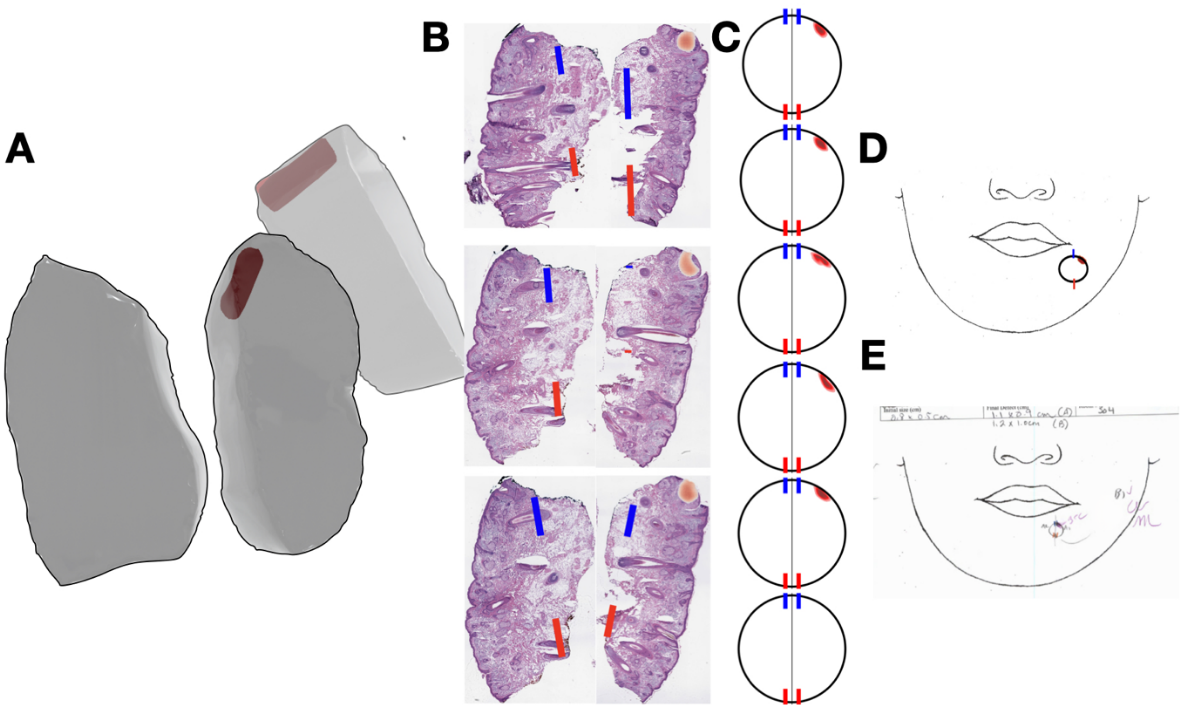

Supplementary Figure 9: Demonstration of 3D surgical tumor mapping process for example patient 2: A) Illustration of 3D histopathology illustrating location of positive tumor margin; B) representative set of tissue sections extracted from multiple tissue layers, illustrating results from AI model to predict tumor margin and automated placement of red/blue ink; C) mapping of histological results to surgical tumor map in standard 2D coordinate system for six serial sections; D) integration of results from serial sections and mapping of predicted histological results to correct position and orientation of tumor map using inks to guide placement in anatomic position/orientation; E) tumor map drawn by surgeon indicating location of positive margins for further resection; subsequent resection stages were removed from these images to draw attention to the initial resection

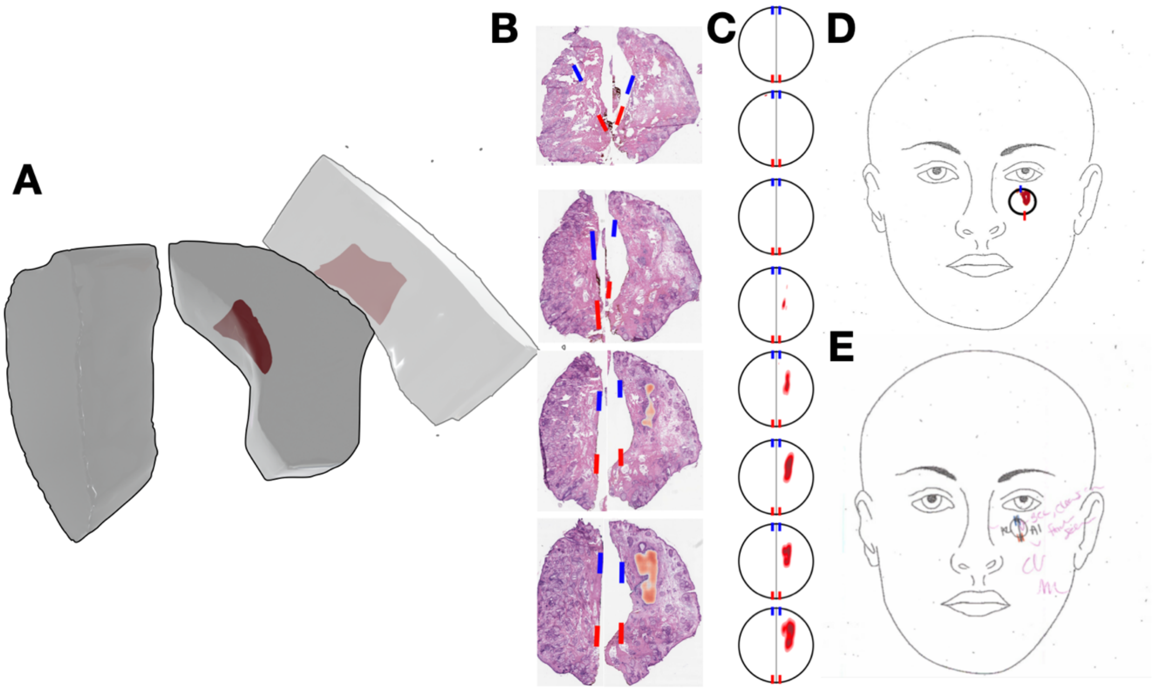

Supplementary Figure 10: Demonstration of 3D surgical tumor mapping process for example patient 3: A) Illustration of 3D histopathology illustrating location of positive tumor margin; B) representative set of tissue sections extracted from multiple tissue layers, illustrating results from AI model to predict tumor margin and automated placement of red/blue ink; C) mapping of histological results to surgical tumor map in standard 2D coordinate system for six serial sections; D) integration of results from serial sections and mapping of predicted histological results to correct position and orientation of tumor map using inks to guide placement in anatomic position/orientation; E) tumor map drawn by surgeon indicating location of positive margins for further resection; subsequent resection stages were removed from these images to draw attention to the initial resection

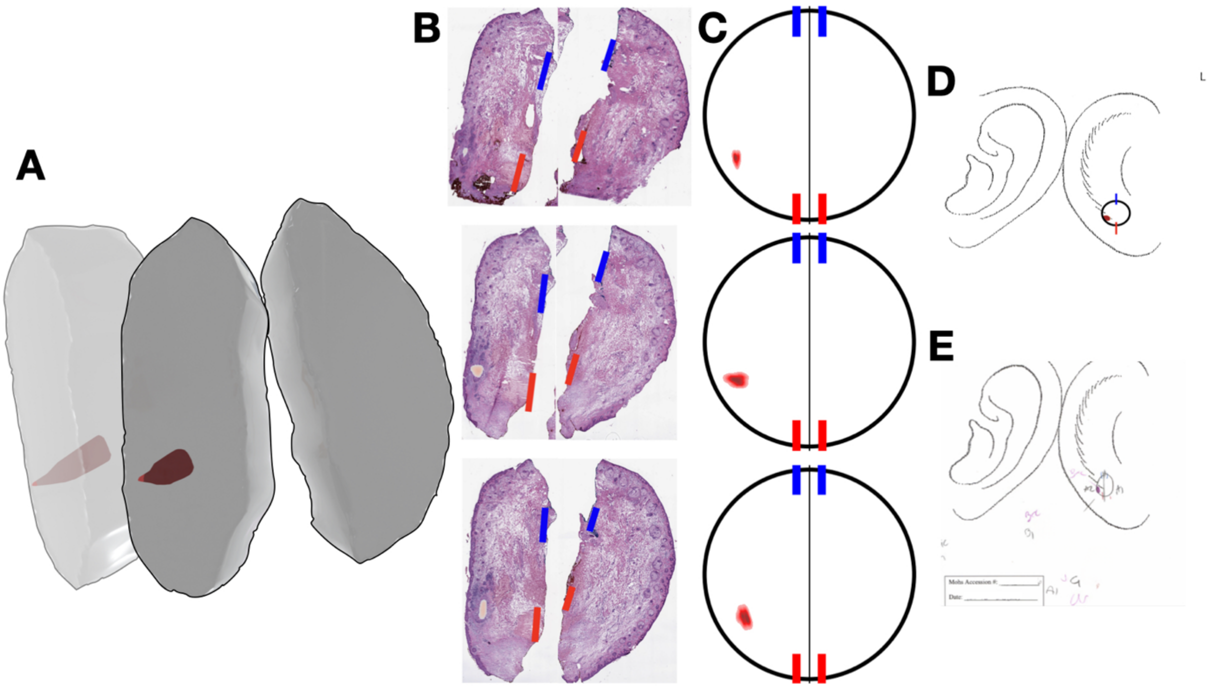

Supplementary Figure 11: Demonstration of 3D surgical tumor mapping process for example patient 4: A) Illustration of 3D histopathology illustrating location of positive tumor margin; B) representative set of tissue sections extracted from multiple tissue layers, illustrating results from AI model to predict tumor margin and automated placement of red/blue ink; C) mapping of histological results to surgical tumor map in standard 2D coordinate system for six serial sections; D) integration of results from serial sections and mapping of predicted histological results to correct position and orientation of tumor map using inks to guide placement in anatomic position/orientation; E) tumor map drawn by surgeon indicating location of positive margins for further resection; subsequent resection stages were removed from these images to draw attention to the initial resection

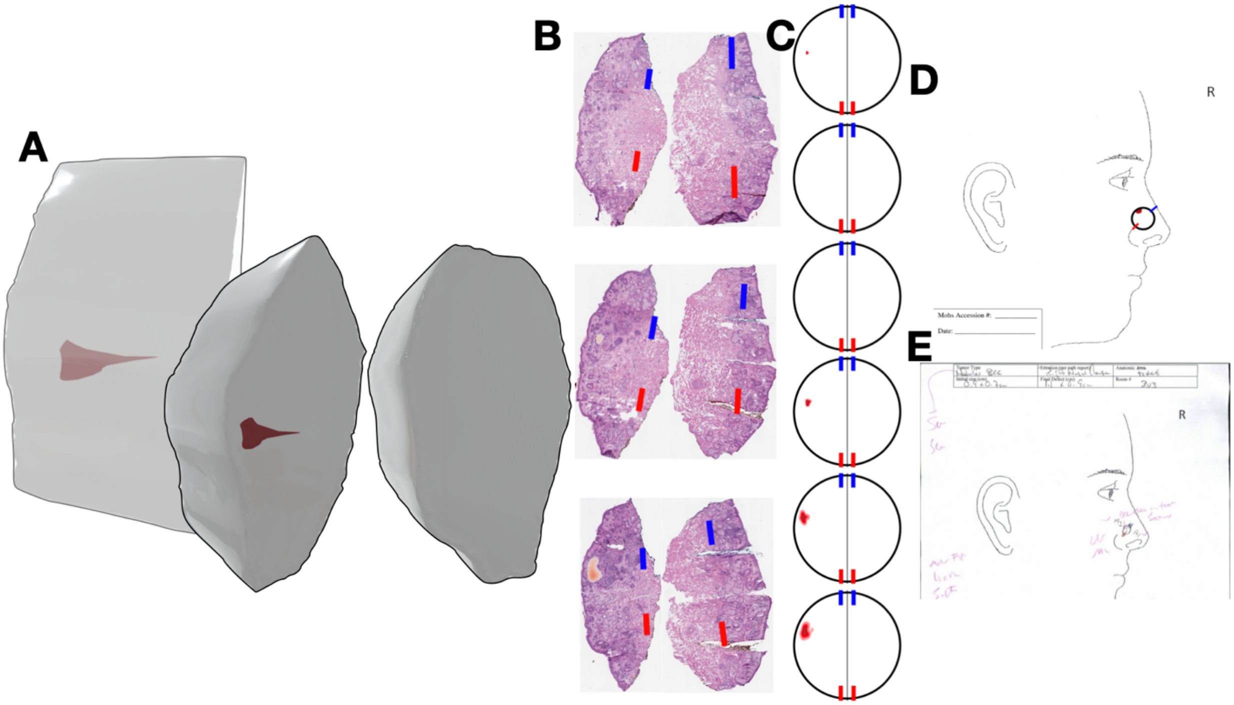

Supplementary Figure 12: Demonstration of 3D surgical tumor mapping process for example patient 5: A) Illustration of 3D histopathology illustrating location of positive tumor margin; B) representative set of tissue sections extracted from multiple tissue layers, illustrating results from AI model to predict tumor margin and automated placement of red/blue ink; C) mapping of histological results to surgical tumor map in standard 2D coordinate system for six serial sections; D) integration of results from serial sections and mapping of predicted histological results to correct position and orientation of tumor map using inks to guide placement in anatomic position/orientation; E) tumor map drawn by surgeon indicating location of positive margins for further resection; subsequent resection stages were removed from these images to draw attention to the initial resection

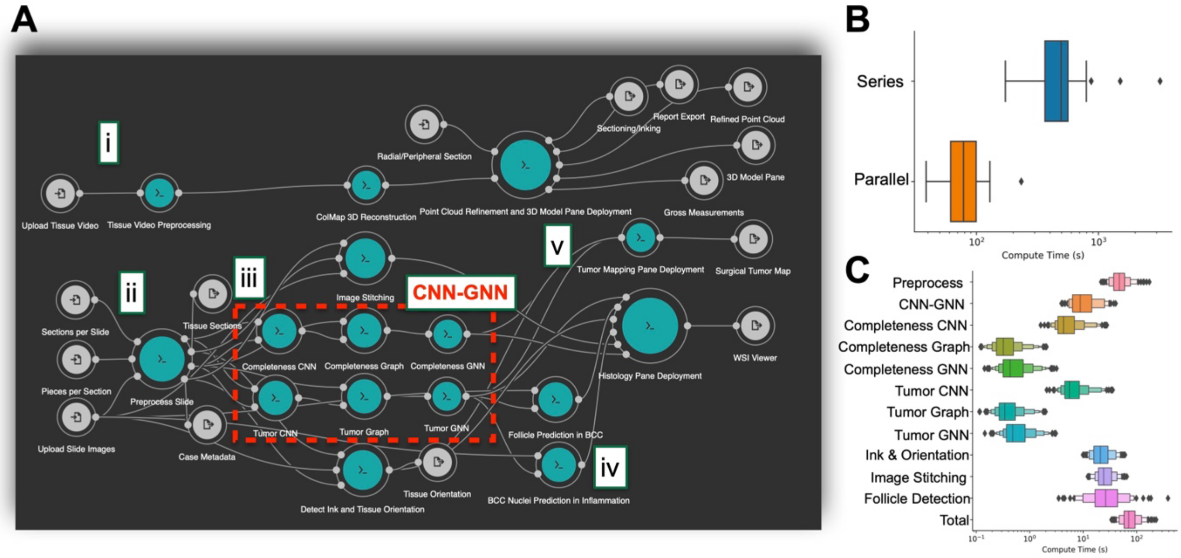

Supplementary Figure 13: Workflow diagram and speed: A) *ArcticAI* workflow diagram, visualized using the Rabix Composer: i) 3D modeling subworkflow, comprised of tissue preprocessing, ColMap reconstruction and point cloud refinement and application deployment; ii-v) histological assessment and tumor mapping subworkflows; ii) slide preprocessing; each tissue section in parallel passes through iii) image stitching, CNN-GNN, and ink/orientation algorithms, which are themselves executed in parallel; iv) optional follicle and nuclei prediction based on Tumor GNN results; finally, results from all sections/WSI are combined for visualization using v) the Histology and tumor mapping panes; B) boxplot denoting total execution time per case for ii-v), given serial and parallel workflow execution; C) boxenplot denoting execution time of ii-v) subcomponents and total given optimized parallel execution across WSI/sections

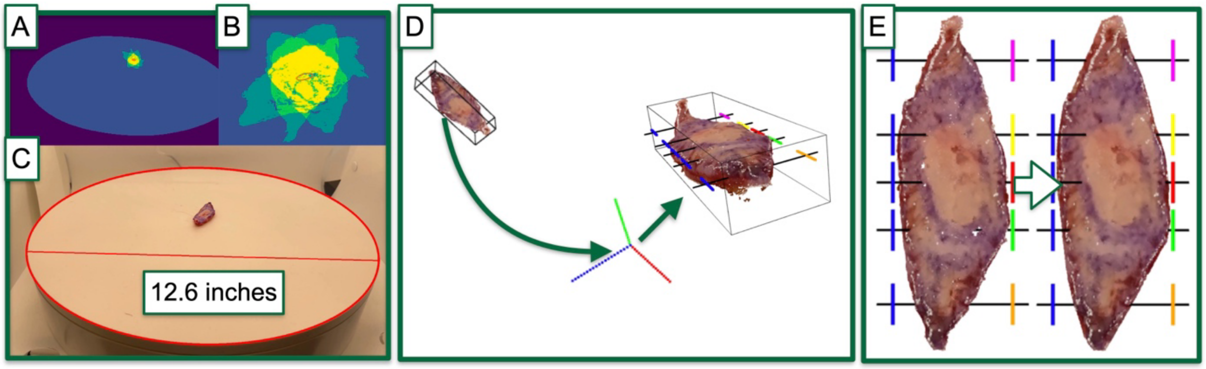

Supplementary Figure 14: Illustration of automated 3D tissue modeling workflow steps: A-C) tissue video preprocessing; A) tissue (yellow; imaged across multiple timesteps) and turntable (blue) are identified in video; B) orange ellipse in middle charts path of center of mass of tissue, used to help delineate tissue versus other image objects; C) calibration of image pixels to length measurements, where red ellipse and line are results from automated ellipse detection algorithm, where double the major axis length is approximated to be the diameter of the turntable, 12.6 inches; D,E) 3D model post-processing; D) reconstructed tissue is initially placed at arbitrary orientation; tissue is filtered and rotated/aligned to origin to automate placement of tissue sections/inks; size is recorded using bounding box; E) 3D tissue model is additionally refined via interpolation to smooth the model for viewing

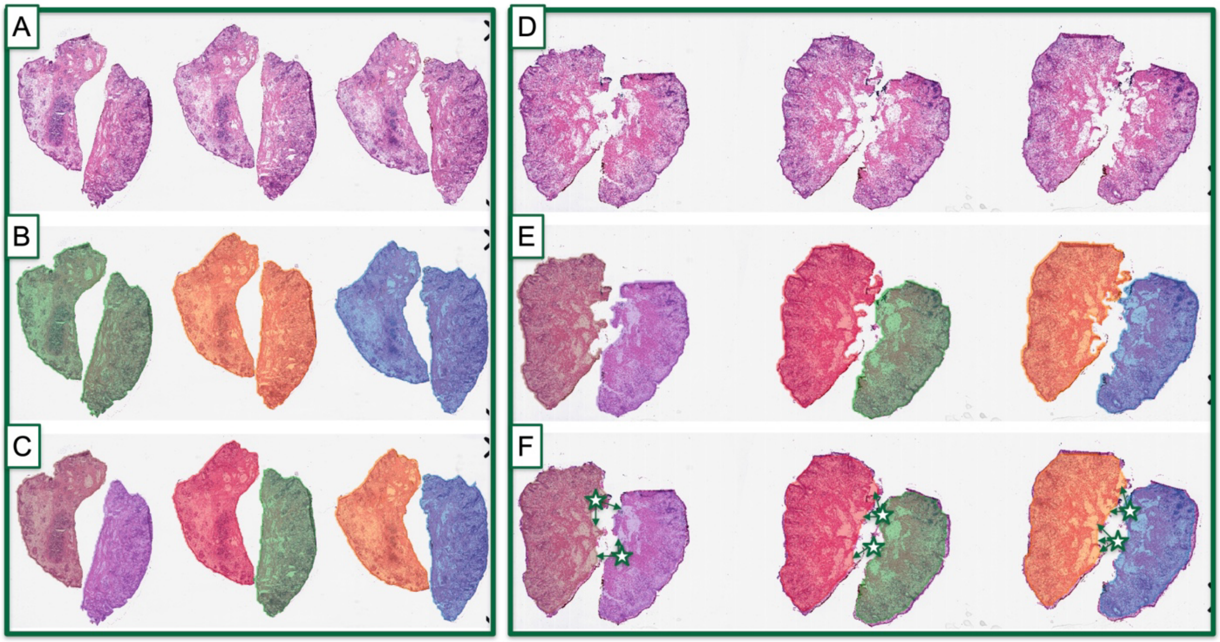

Supplementary Figure 15: Histological assessment WSI preprocessing and separation of problematic conjoined sections for two cases: A) original slide image (of three WSI) for first patient; B) tissue section assignment via 3 colors, where there are two conjoined pieces per section; C) separation of conjoined pieces for each section yields a unique piece identifier, each of which is separately processed via the histological assessment subworkflow; D) original slide image (of three WSI) for second patient; E) illustration of tissue patches selected for Tumor CNN-GNN algorithm; F) illustration of tissue patches selected for Completeness CNN-GNN algorithm, where arrows emanating from stars denote location of candidate tears which are normally missed but here are picked up through an alpha shape algorithm

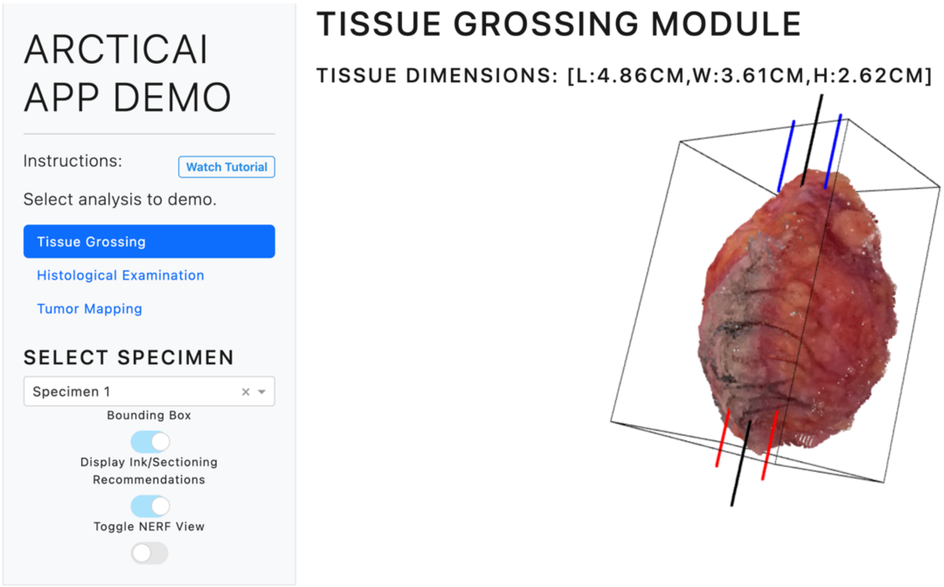

Supplementary Figure 16: Illustration of tissue grossing panel for sample specimen, demonstrated using interactive web application; orientation indicators (red/blue), vertical black line and box drawn to indicate places to ink, bisect specimen, and measure tissue dimensions respectively

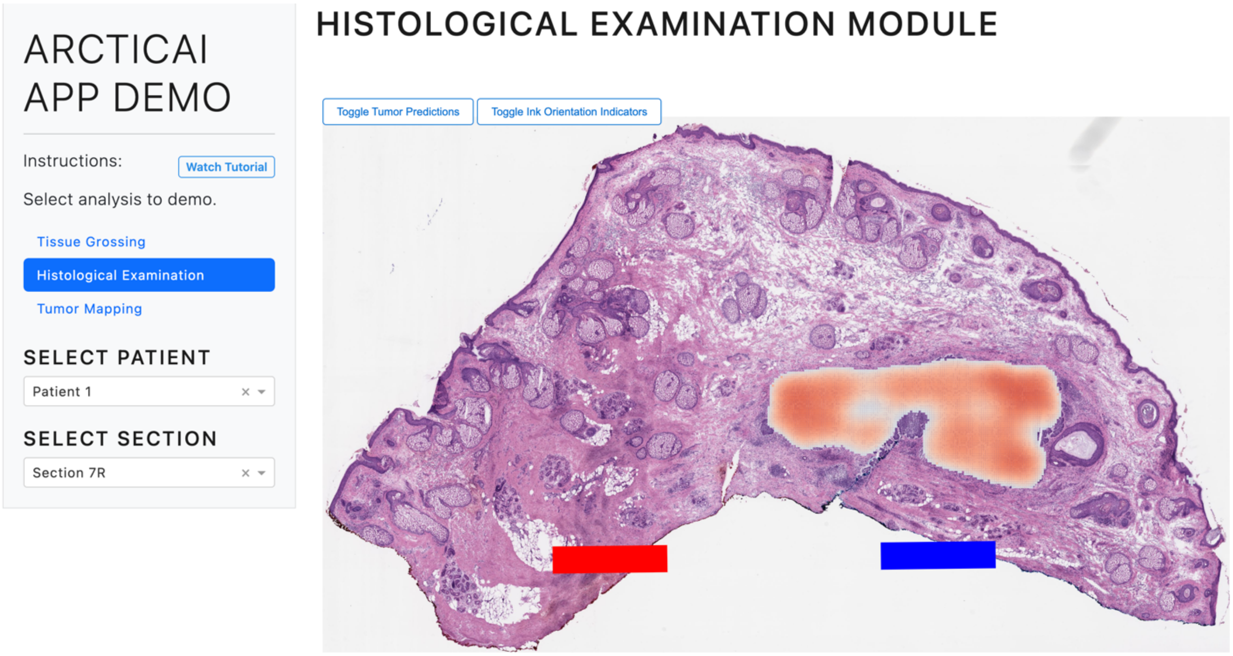

Supplementary Figure 17: Illustration of histological assessment panel for sample patient at seventh serial section, demonstrated using interactive web application; tissue orientation as derived using detected blue and red inks and tumor histological findings using heatmap were toggled using the zoomable whole slide image viewer

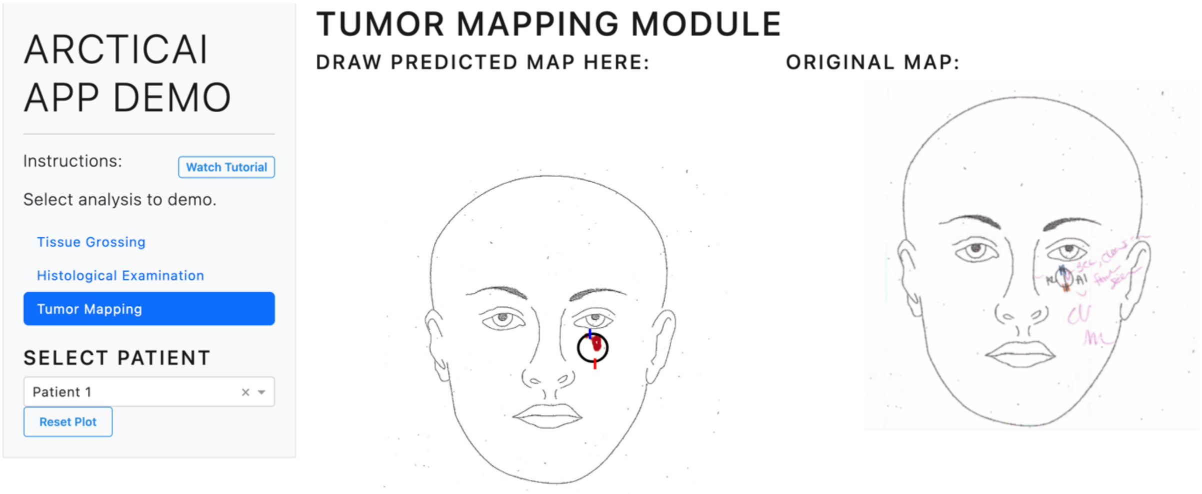

Supplementary Figure 18: Illustration of tumor mapping panel for sample patient, demonstrated using interactive web application; circle, blue and red inks drawn by user to indicate anatomic location of specimen and orientation respectively, with red heatmap drawn to indicate location of positive margin as reported using the neural networks; results compared to the original tumor map on right

Supplementary Tables

Supplementary Table 1: Case Count: Number of cases, slides, tissue sections, pieces and annotations comprising the training/validation and test sets for the margin assessment (completeness, tumor localization) and ink orientation algorithms, where number of WSI are also broken down by BCC histological subtype and clear margins (in the test set as controls). Note that some of the WSI featured in the BCC subtype breakdown are double counted, as they represent several histological subtypes

		Tumor and Holes/Tears		Test Set: Inking Patterns
	**Total**	**Training/Validation**	**Test**	**Tissue Orientation**
**Cases**	178	76	41	31
**Slides**	351	122	121	108
**Sections**	1065	381	360	324
**Tissue Pieces**	1537	472	559	506
**Number Annotations**	16,128	11,343	2,535	2,250

	**Training/Validation**	**Test**
**Superficial**	36	21
**Nodular**	102	58
**Micronodular**	11	6
**Infiltrative**	20	23
**Sclerosing**	6	0
**Microcystic**	2	2
**Squamatized**	0	3
**Nodular/Micronodular**	0	1
**No tumor**	0	45

Supplementary Table 2: Tissue size measurements (length, width, height), measured empirically (left) and predicted with the automated tissue grossing tool (point cloud and neural network based, NeRF), in centimeters (cm). Also reported was whether the excision type was a radial section for breadloafing or circumferential excision for the assessment of peripheral margins.

**Specimen**	**Excision Type**	**Measured (cm)**			**Predicted (cm) 3D Point Cloud**			**Predicted (cm) NeRF**		
		L	W	H	L	W	H	L	W	H
**0748**	Radial	2.4	1.3	0.6	2.38	1.23	0.79	2.35	1.32	0.63
**0749**	Radial	4.5	4	3.3	4.86	3.61	2.61	4.43	3.89	3.49
**0750**	Peripheral	2.8	2	2.2	2.43	2.17	1.73	2.61	2.24	2.16
**0751**	Radial	4.5	2	2.5	4.64	2.52	2	4.69	2.21	2.23
**0752**	Peripheral	4.5	3.5	3	4.87	3.49	2.98	3.97	3.33	3.37
**0753**	Peripheral	2	2	0.6	2.02	1.98	1.05	2.33	2.29	0.96
**0754**	Peripheral	3.5	2.5	2	3.97	3.3	1.9	3.39	2.57	2.13
**0756**	Radial	2	1	0.7	1.68	0.88	0.45	2.09	0.81	0.95
**0796**	Radial	3	1.5	0.5	3.84	1.98	0.78	3.43	1.67	0.89
**0798**	Radial	3	1.5	0.5	2.47	1.27	0.85	3.9	1.71	0.49
**6693**	Peripheral	1.5	1.3	0.7	1.54	1.28	0.8	1.33	1.52	1.1
**6694**	Peripheral	2.9	2.4	1.1	2.86	2.2	1.09	3.14	2.2	0.93
**6696**	Peripheral	2	1.5	0.6	2.23	2.06	1.14	1.89	1.36	0.95
**6697**	Radial	5.1	1.7	0.6	3.46	1.26	0.79	4.4	1.51	0.86
**6698**	Radial	2.8	1.3	0.7	3.96	2.03	1.34	3.58	1.67	1.2
**6699**	Radial	1.7	1	0.5	1.91	1.13	0.79	1.49	0.82	0.81
**6700**	Peripheral	2.1	1.3	1	3.59	2.05	1.53	2.65	1.83	1.34

Supplementary Table 3: 3D Specimen Modeling Time, measured empirically (left) and predicted with the automated tissue grossing tool (point cloud and neural network based), in centimeters (cm).

Specimen	NeRF Runtime (s)	3D Point Cloud Runtime (s)
**0748**	27.4	114.9
**0749**	32.4	85.3
**0750**	29.6	82.7
**0751**	25.8	101.3
**0752**	28.9	100.1
**0753**	25.3	83.6
**0754**	30.6	106.7
**0756**	34.7	78.9
**0796**	30.4	118.8
**0798**	30.9	127.2
**6693**	30.4	89
**6694**	27.1	95.1
**6696**	25.3	75.8
**6697**	26.8	98.4
**6698**	28	77.2
**6699**	31.7	85.9
**6700**	30.5	121.4
**Average**	29.2 ± 2.7	96.6 ± 16.4

Supplementary Table 4: Impact of modeling inflammation within internal validation set for model selection; comparison of AUCs for different modeled classes for internal validation set with and without incorporation of inflammation into the set; for both models, the proportion of 64-micron patches where inflammatory regions were accidentally labeled as tumor was estimated, indicating that models that did not account for inflammation were nearly ten-times more likely to call tumor for pockets of inflammatory cells

				Two-Class: Benign vs. Tumor Model		
	**AUC**	**2.5% CI**	**97.5% CI**	**% Inflammatory Patches Conflated with Tumor**	**2.5% CI**	**97.5% CI**
**Benign**	0.957	0.955	0.958	-	-	-
**Inflammation**	-	-	-	38.76%	35.56%	41.96%
**Tumor**	0.957	0.955	0.958	-	-	-
				**Three-Class: Benign vs. Inflammation vs. Tumor Model**		
**Benign**	0.969	0.968	0.97	-	-	-
**Inflammation**	0.953	0.951	0.956	4.09%	3.62%	4.56%
**Tumor**	0.981	0.98	0.982	-	-	-
**Difference in Proportions between Methods, % Inflammatory Patches Conflated with Tumor**					**z-score**	-35.622
					**p-value**	6.21e-278

Supplementary Table 5: CNN-GNN Margin Assessment Model Performance Across Different BCC Histological Subtypes. Macro-AUC represents reporting of AUC statistic on slide level and averaging across slides, giving each slide equal weight, while normal AUC statistic is calculated for subimages across all slides. 95% confidence intervals were acquired using 1000-sample non-parametric bootstrap, where bootstrapping was done on the WSI level to account for variation in concordance across the cases.

	**Weighted-Average**			**Macro-Averaged**		
	**AUC**	**2.5% CI**	**97.5% CI**	**AUC**	**2.5% CI**	**97.5% CI**
**Infiltrative**	0.963	0.956	0.979	0.961	0.945	0.974
**Microcystic**	0.960	0.955	0.963	0.959	0.955	0.963
**Micronodular**	0.972	0.962	0.994	0.953	0.896	0.990
**Nodular**	0.975	0.967	0.981	0.969	0.960	0.977
**Squamatized**	0.984	0.961	0.996	0.982	0.961	0.996
**Superficial**	0.963	0.948	0.973	0.954	0.944	0.964

Supplementary Table 6: Nuclei Prediction Workflow Accuracy

**Task**	**Model**	**Metric**	**Score**	**2.5% CI**	**97.5% CI**
**Nuclei Detection**	**Detectron**	AP50 (0-1)	0.224	0.220	0.227
		Dice Coefficient	0.847	0.846	0.847
**Nuclei Classification**	**Detectron**	F1-Score	0.684	0.682	0.685
	**Cell-CNN**	F1-Score	0.775	0.774	0.776
	**Cell-GNN**	F1-Score	0.856	0.852	0.856

Supplementary Table 7: Comprehensive performance characteristics for tissue orientation results derived from identification of inks from histological sections; with focus on identifying proportion of sections in correct orientation based on a sensitivity analysis of minimally allowable angular differences between observed and predicted tissue orientation derived from ink; 95% confidence intervals derived using 1000-sample non-parametric bootstrapping

Maximal Angular Difference (°)	Proportion Correct Orientation	2.5% CI	97.5% CI
**1**	13.6	10.2	16.9
**2**	26.6	22	30.8
**3**	38.3	33.7	42.6
**4**	45.3	40.4	50.1
**5**	51.3	46.7	55.7
**6**	58.4	53.5	63.4
**7**	64.6	60.3	69.2
**8**	70	65.6	74.6
**9**	74.3	70	78.7
**10**	76.5	72.4	80.4
**11**	78.9	75.1	82.8
**12**	81.4	77.5	85
**13**	82.8	79.2	86.4
**14**	84.7	81.6	87.9
**15**	86	82.6	88.9
**16**	86.4	83.1	89.6
**17**	87.4	84.3	90.8
**18**	87.7	84.3	90.8
**19**	88.4	85.2	91.5
**20**	89.1	86.2	92
**21**	89.3	86.2	92.3
**22**	89.8	86.9	92.5
**23**	90.1	86.9	92.7
**24**	90.1	87.2	92.7
**25**	90.8	88.1	93.5
**26**	91.3	88.6	93.9
**27**	91.5	88.9	94.2
**28**	91.5	88.6	94.2
**29**	91.5	88.9	94.2
**30**	92	89.3	94.4
**31**	92.5	90.1	94.9
**32**	92.5	89.8	94.9
**33**	92.7	90.3	94.9
**34**	93.2	90.8	95.4
**35**	93.5	91	95.6
**36**	93.7	91	96.1
**37**	93.9	91.8	95.9
**38**	93.9	91.5	95.9
**39**	93.9	91.8	96.1
**40**	94.2	92	96.4
**41**	94.2	92	96.4
**42**	94.2	91.8	96.1
**43**	94.2	92	96.4
**44**	94.2	91.8	96.4
**45**	94.7	92.5	96.6
**46**	94.7	92.3	96.9
**47**	94.9	93	96.9
**48**	95.2	93	97.1
**49**	95.4	93.2	97.3

Supplementary Table 8: Average GPU execution time for workflow subcomponents. Final times for a case were given by the maximum compute time across sections for the case after preprocessing, using GPUs for all neural network tasks. After preprocessing a WSI, the CNN-GNN, Tissue Orientation and Image Stitching tasks execute in parallel, as do all sections in the specimen, where the section that takes the longest serves as the bottleneck. Within the CNN-GNN tasks, the CNN-GNN (broken into serial CNN, graph generation, and GNN subcomponents) for tissue completeness and tumor run in parallel. 95% confidence intervals for median time statistics were estimated via 1000 non-parametric bootstrapped resamplings

Task	Subtask	Median(s)	2.5% CI	97.5% CI
**Tissue Preprocessing**		47.68	44.21	51.22
**CNN-GNN**	**Total**	8.84	7.05	11.4
	**Completeness CNN**	4.31	4.12	4.5
	**Tumor CNN**	5.58	5.28	5.9
	**Completeness Graph Generation**	0.32	0.3	0.35
	**Tumor Graph Generation**	0.34	0.33	0.36
	**Tumor GNN**	0.48	0.45	0.55
	**Completeness GNN**	0.43	0.41	0.49
**Tissue Orientation**		20.96	18.52	24.15
**Image Stitching**		24.39	22.22	28.81
**Total per WSI**	**Parallel**	71.57	66.4	79.23
**Total per Case**	**Parallel**	78.49	65.74	87.73
	**Series**	493.82	367.49	553.44

Supplementary Table 9: Average CPU execution time for workflow subcomponents. Final times for a case were given by the maximum compute time across sections for the case after preprocessing, using **CPU**s for all neural network tasks. After preprocessing a WSI, the CNN-GNN, Tissue Orientation and Image Stitching tasks execute in parallel, as do all sections in the specimen, where the section that takes the longest serves as the bottleneck. Within the CNN-GNN tasks, the CNN-GNN (broken into serial CNN, graph generation, and GNN subcomponents) for tissue completeness and tumor run in parallel. 95% confidence intervals for median time statistics were estimated via 1000 non-parametric bootstrapped resamplings

Task	Subtask	Median(s)	2.5% CI	97.5% CI
**Tissue Preprocessing**		47.68	44.21	51.22
**CNN-GNN**	**Total**	49.21	34.84	59.01
	**Completeness CNN**	30.70	26.99	35.01
	**Tumor CNN**	36.18	31.98	40.47
	**Completeness Graph Generation**	0.32	0.30	0.34
	**Tumor Graph Generation**	0.36	0.35	0.38
	**Tumor GNN**	0.46	0.43	0.50
	**Completeness GNN**	0.41	0.37	0.44
**Tissue Orientation**		20.96	18.52	24.15
**Image Stitching**		24.39	22.22	28.81
**Total per WSI**	**Parallel**	91.89	79.81	110.59
**Total per Case**	**Parallel**	95.55	78.54	132.62
	**Series**	1392.2	907.22	1817.02

Supplementary Videos

Supplementary Video 1: Video of 3D point cloud modeling workflow for three cases, A-C): tissue (left) are extracted frame-by-frame from turntable videos (top) and used to generate 3D models (right), where size is recorded and inking/sectioning recommendations are made

Supplementary Video 2: Video of 3D neural radiance field modeling results for three cases, A-C)**:** Screen recording of interactive neural radiance field (NeRF) display demonstrating addition of tissue orientation indicators through a custom programming package and improved 3D modeling over the point cloud methods.

Supplementary Video 3: Video illustration of surgical tumor mapping from histological findings in real-time: A) viewing predicted tumor and inks from one tissue section via histology pane; B) histological findings and inks are mapped to circle via morphing model; C) real-time video of operating the mapping pane to place a tumor mapping area, from which the predicted tumor map is automatically plotted; D) predicted surgical tumor map; E) hand-drawn tumor map

Supplementary Video 4: Video illustration of 3D histopathology: 3D models of serial tissue sections derived through co-registered serial whole slide images for four separate patients; segmented tumor in red is connected between the serial layers to yield a 3D model of the tumor; 3D histopathology of these tumors lack tissue orientation/position normally derived using inks; surgical tumor map compares favorably to 3D histopathology as a surgical display for surgeons

Supplementary Data

Supplementary Data 1: Comparison of hand-drawn surgical tumor maps (left) to algorithm predicted tumor maps (right): Expanded set of comparisons via PDF file for majority of test set cases; several resection stages have been included on some of these diagrams, though emphasis is placed on the initial resection. See subsequent pages.

Hand Drawn and Predicted Surgical Tumor Maps

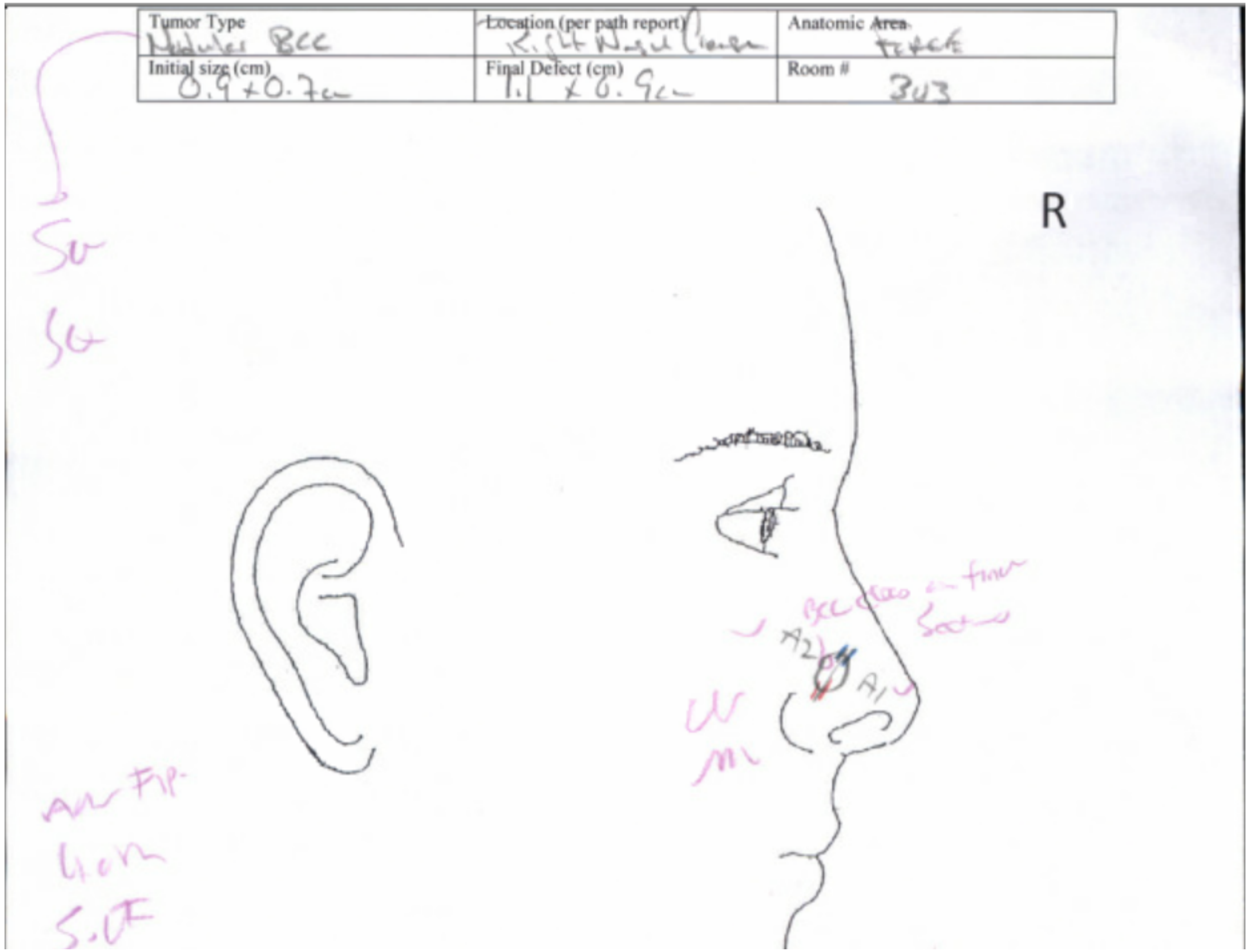

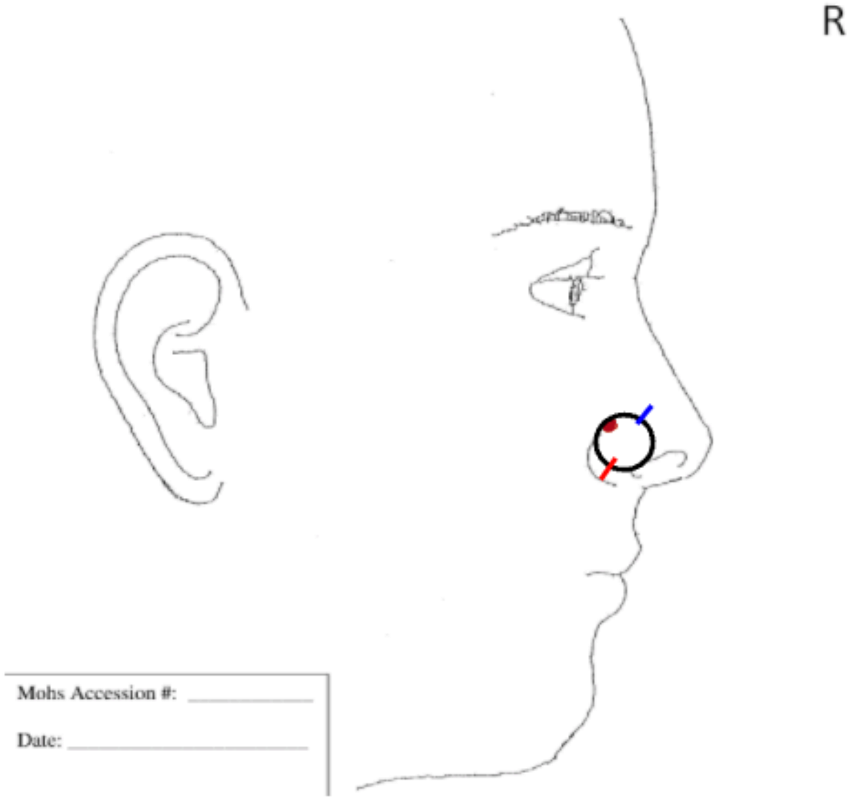

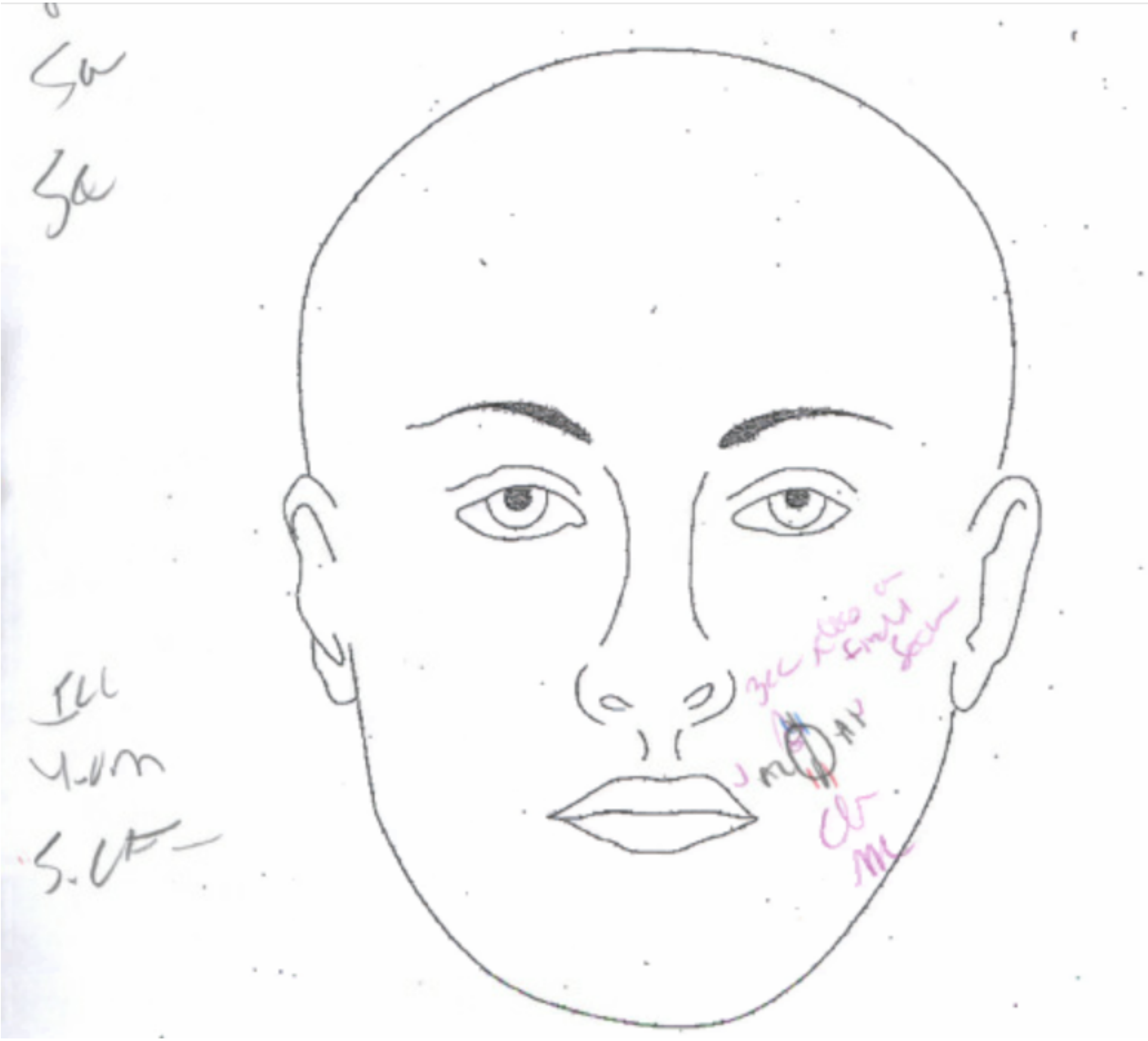

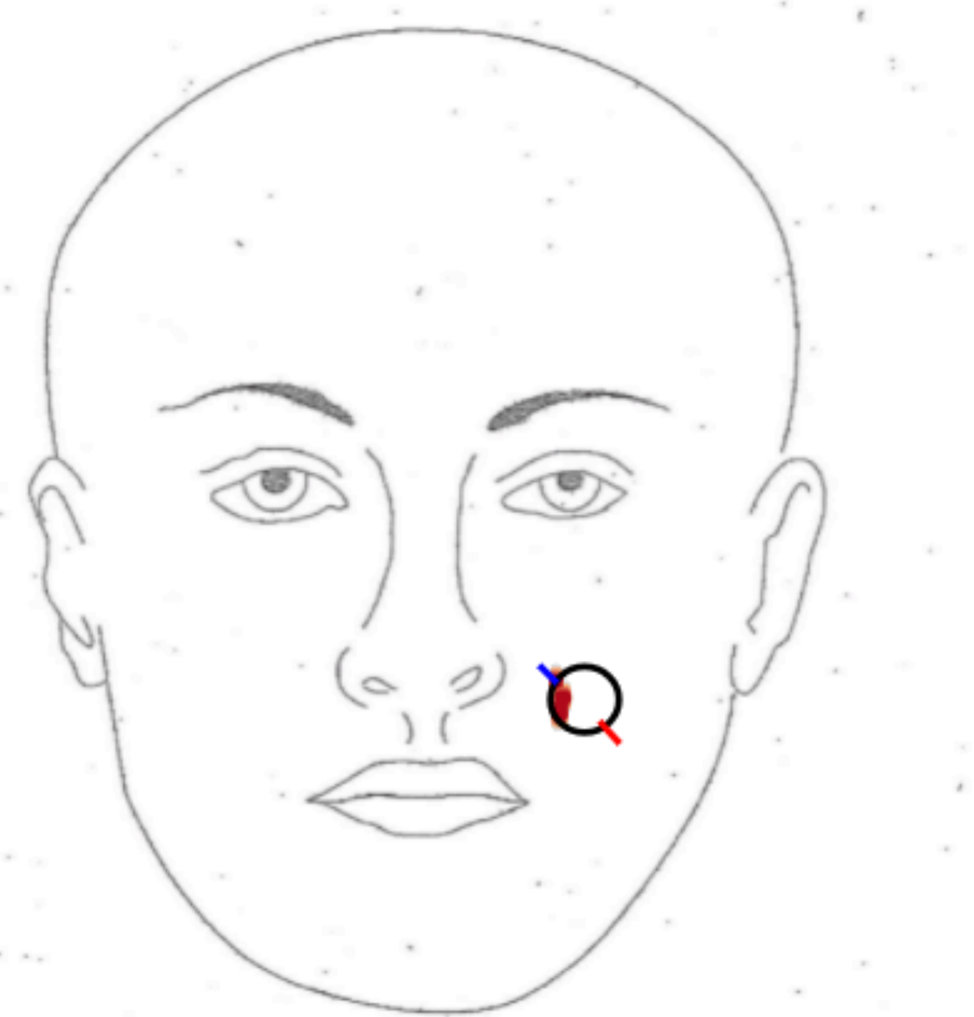

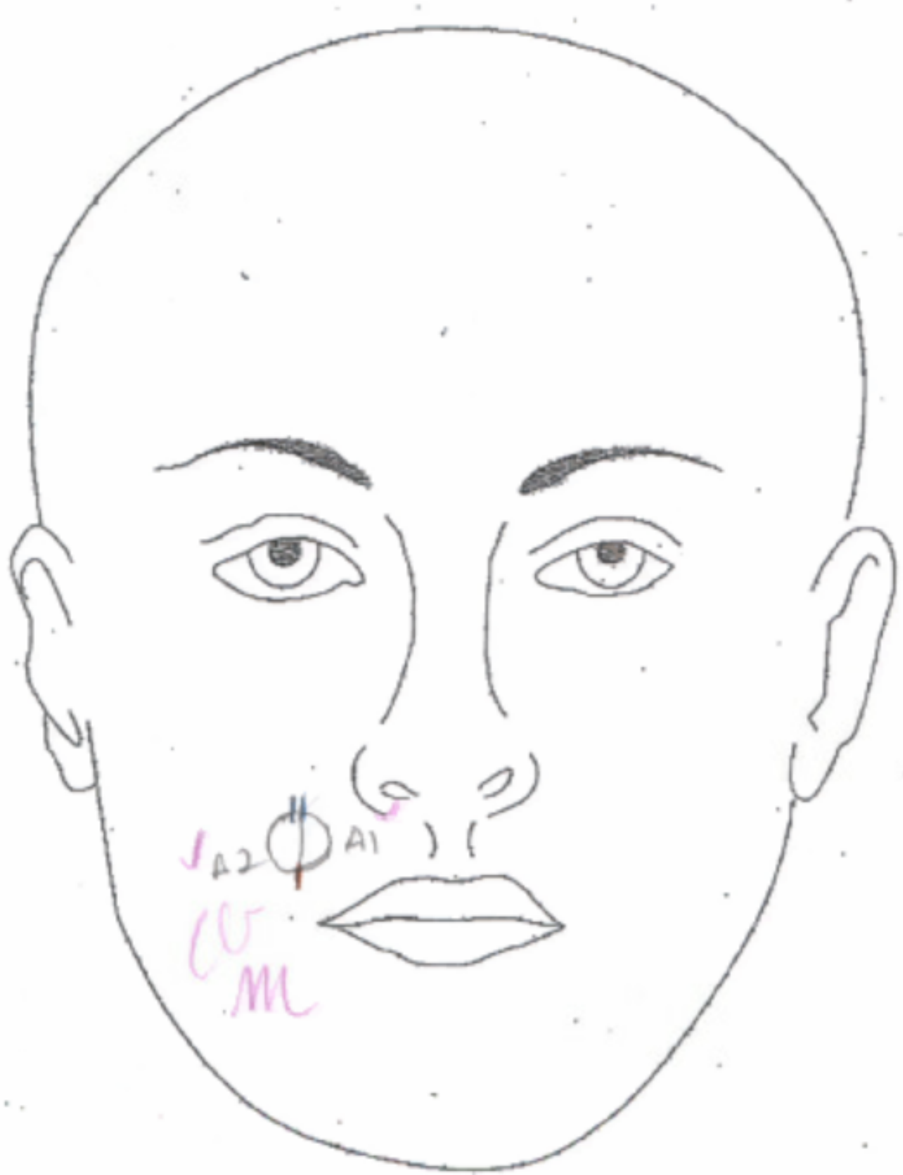
A2
A1

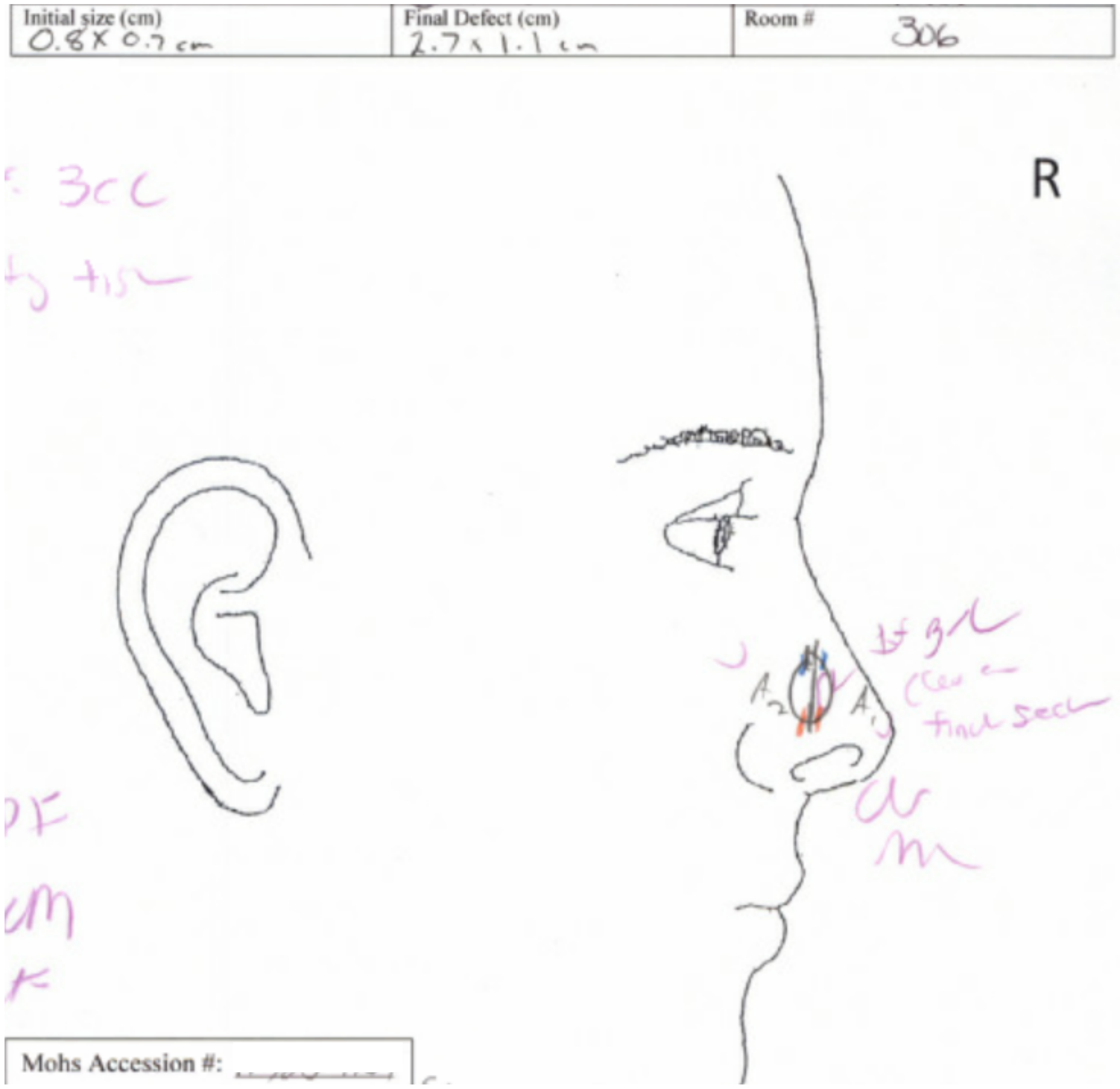
Initial size (cm)
0.8 X 0.7 cm
Final Defect (cm)
2.7 x 1.1 cm
Room #
306
R
Mohs Accession #:

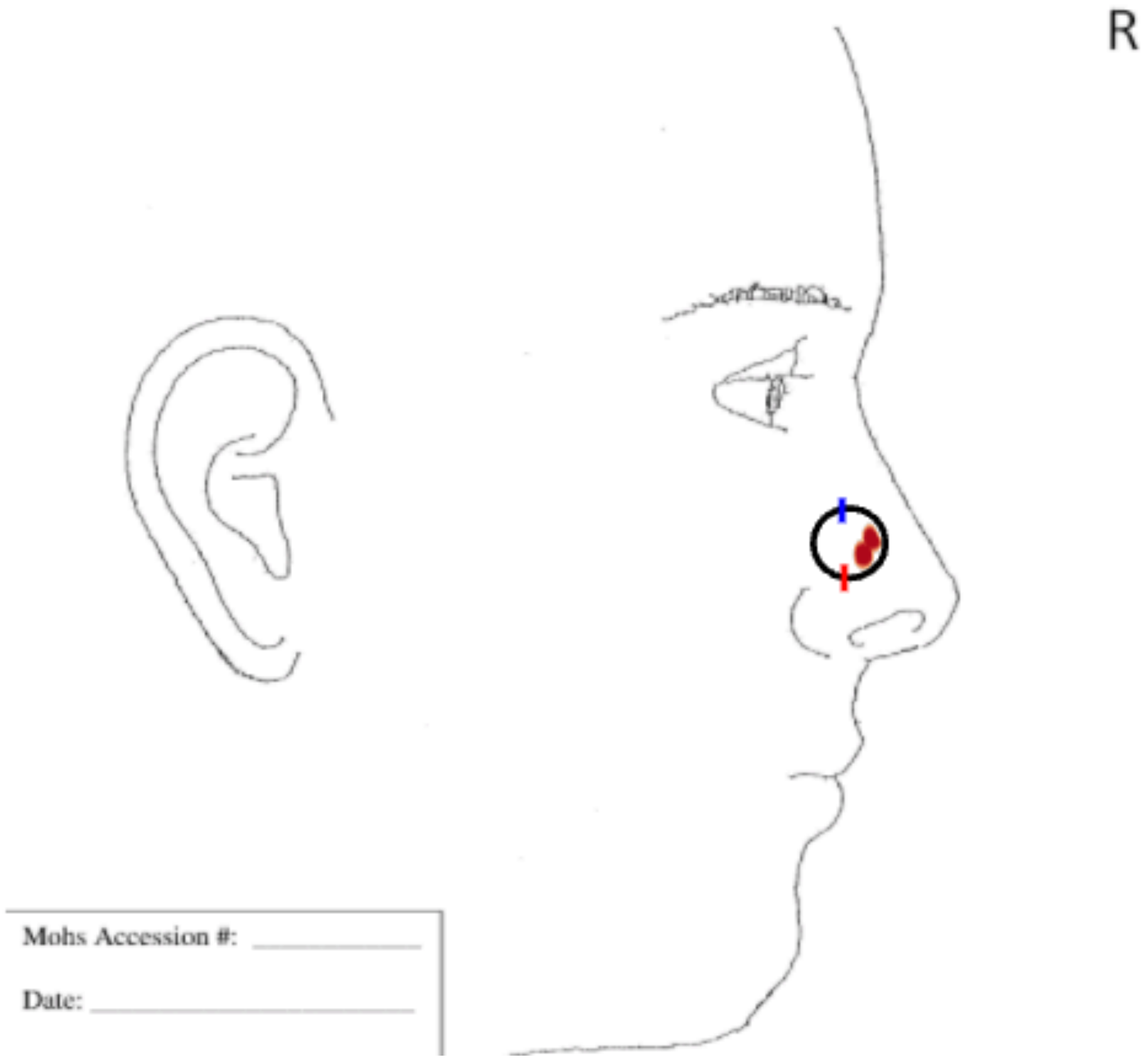
R
Mohs Accession #:
Date:

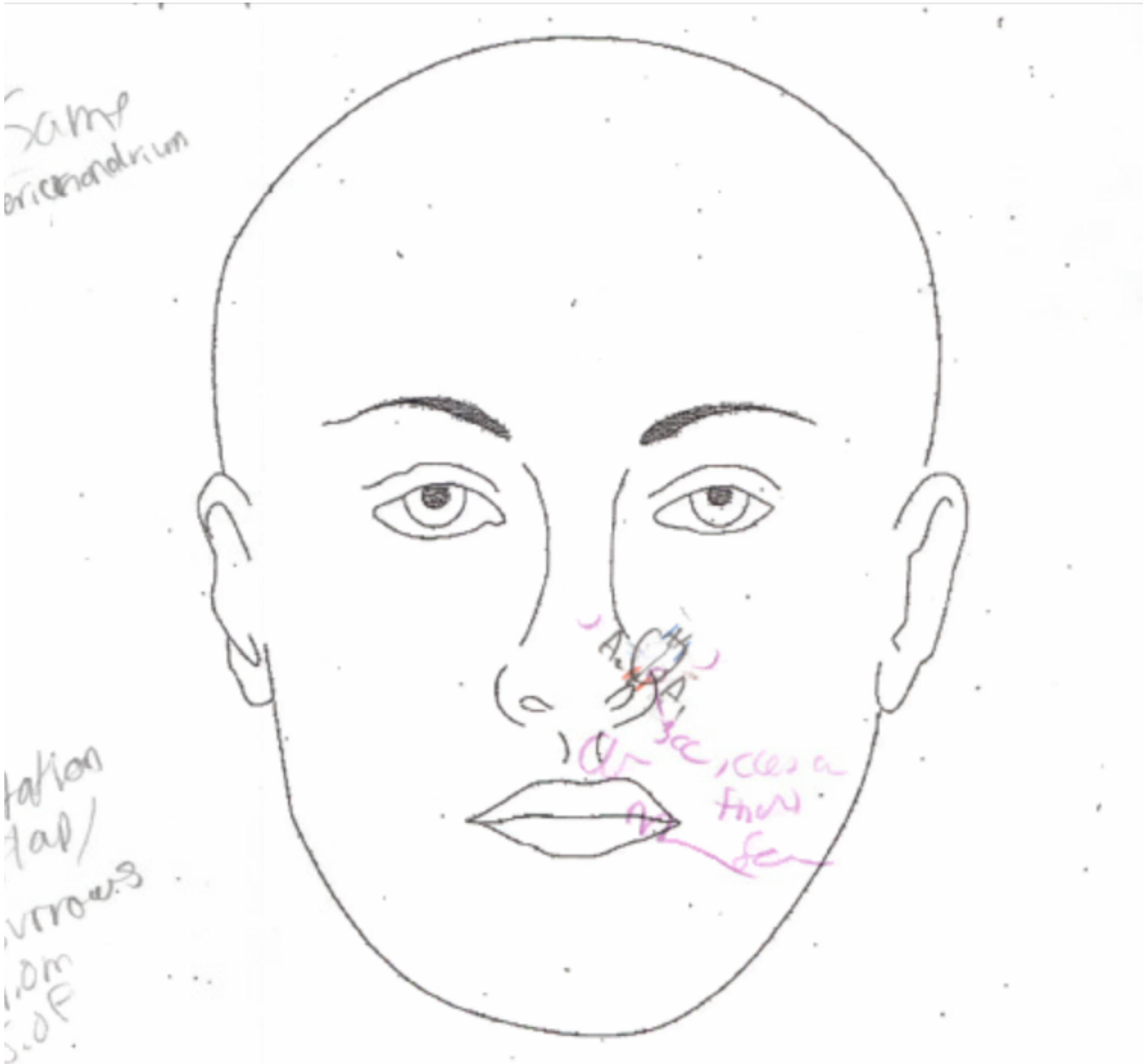

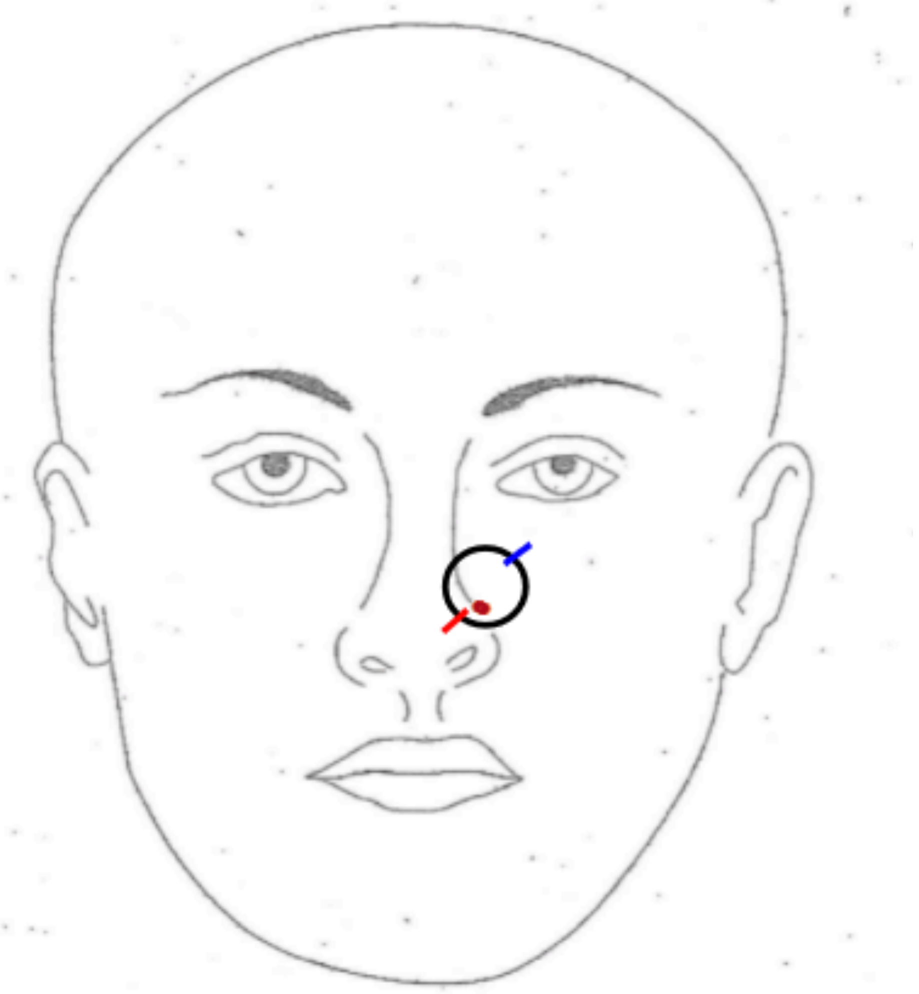

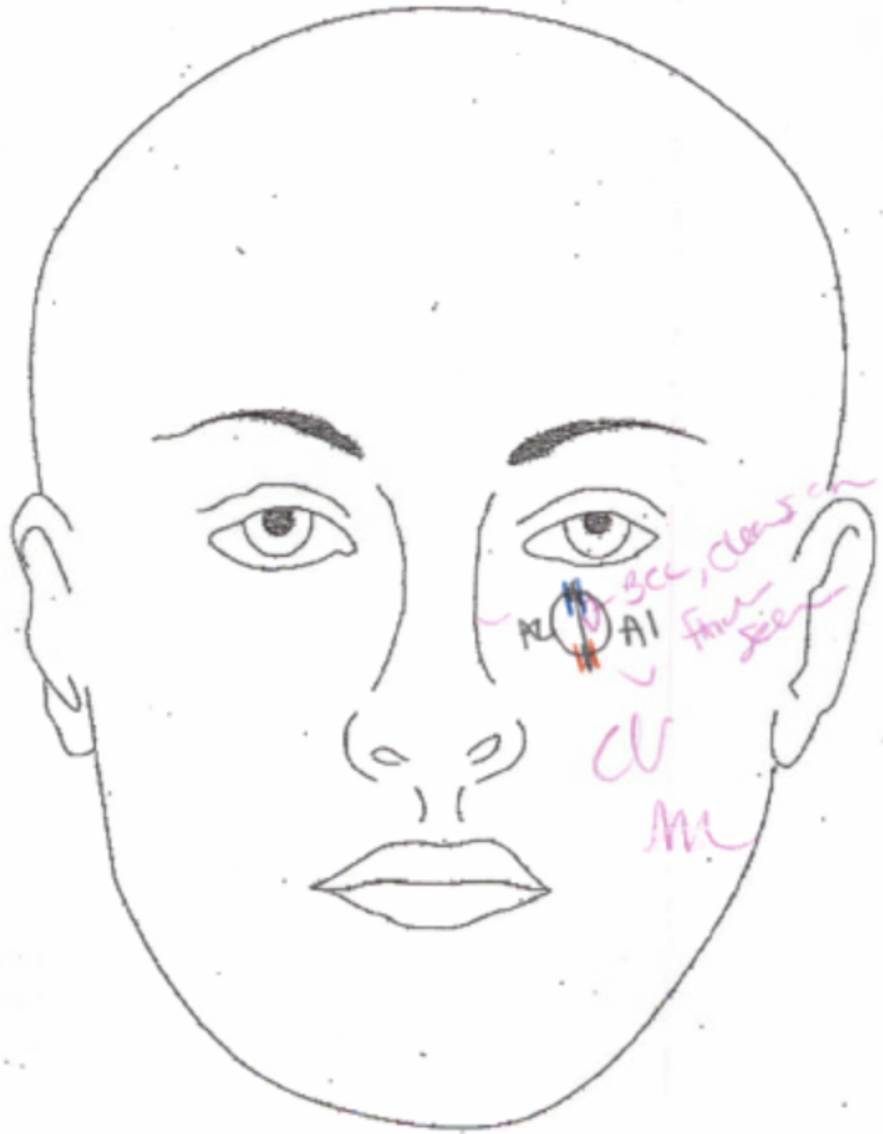
AI
3cc, clean
CU

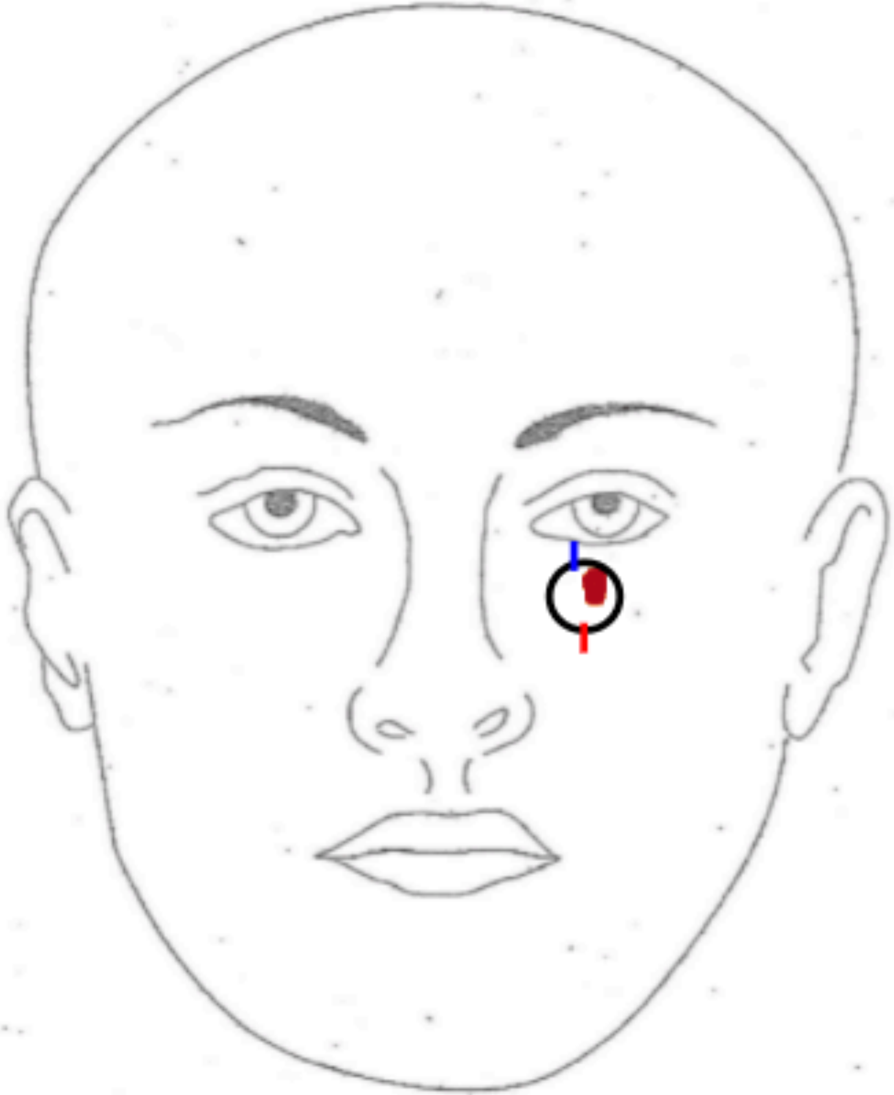

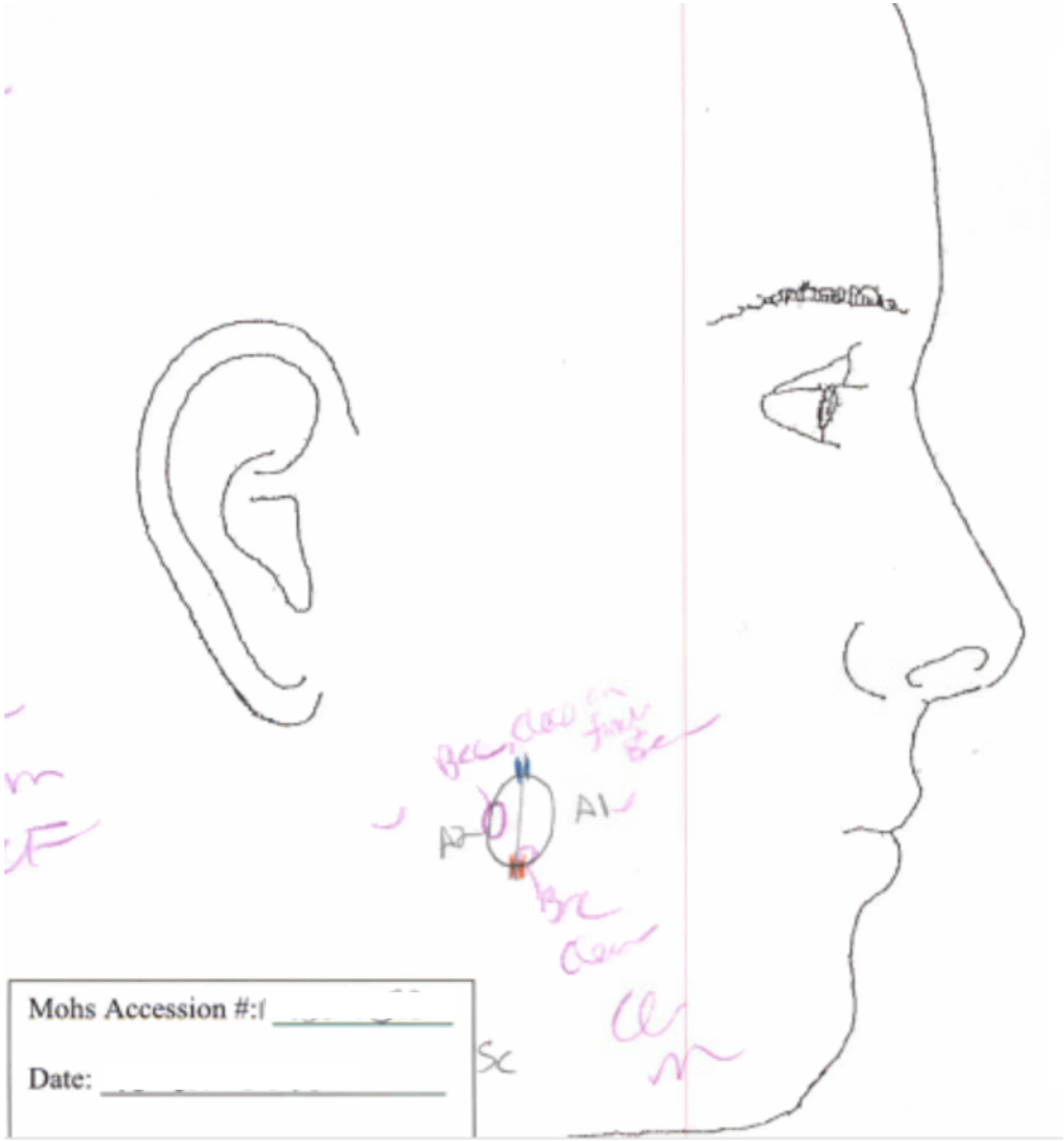
Mohs Accession #:
Date:

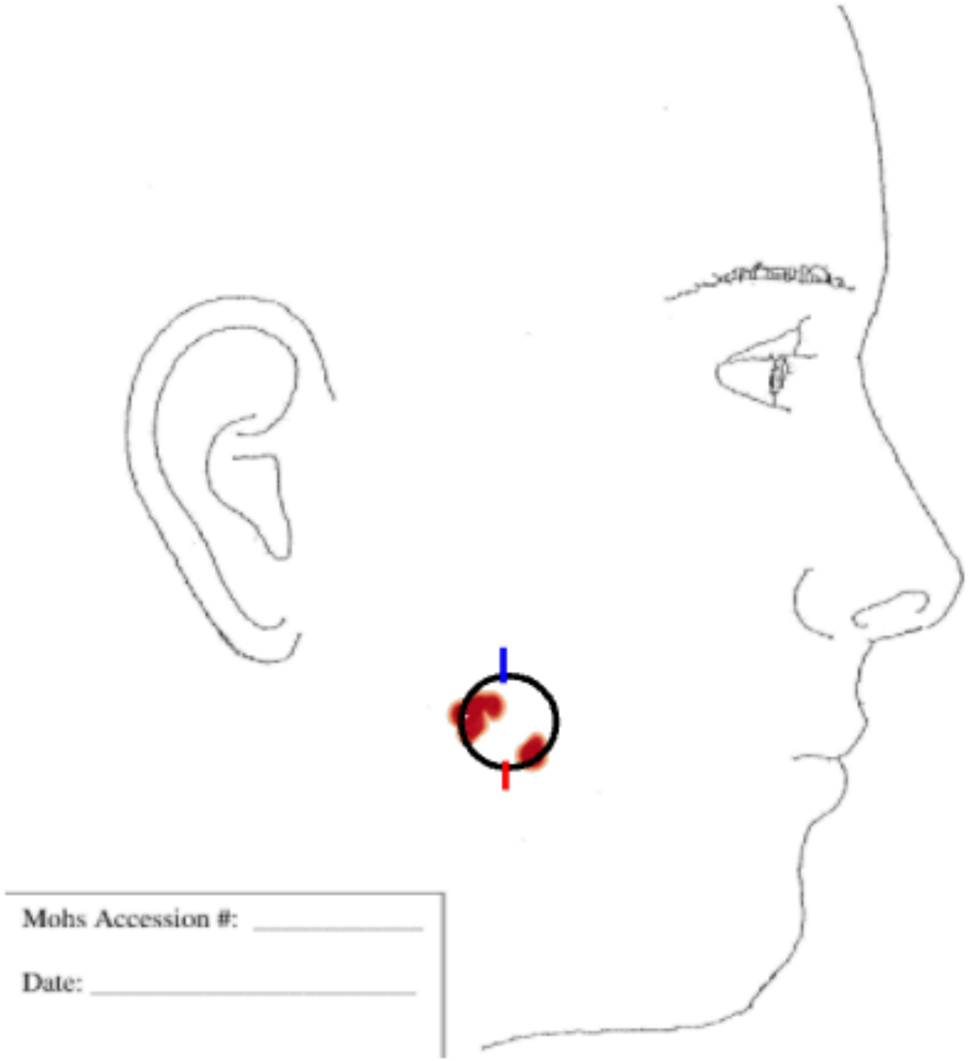
Mohs Accession #:
Date:

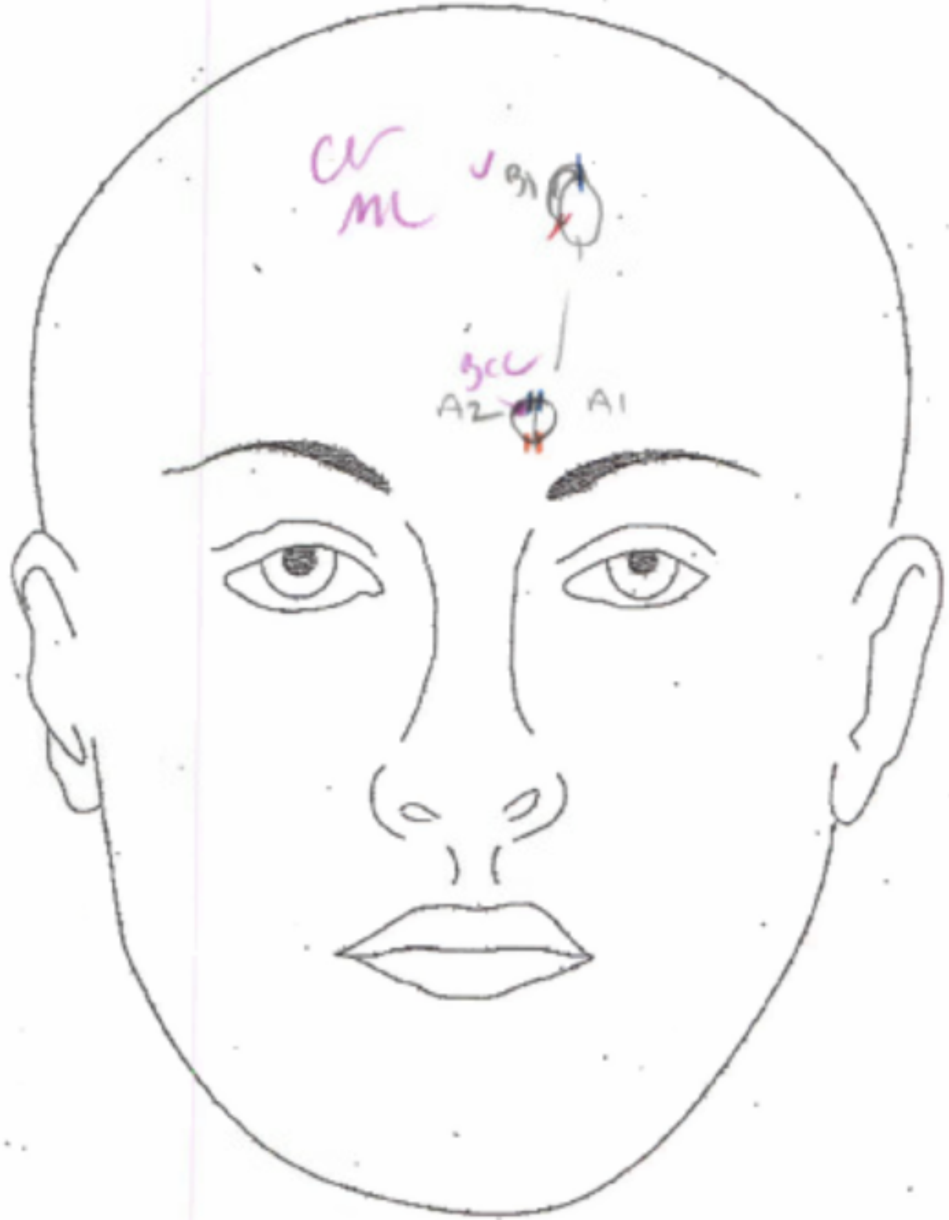
A2
A1

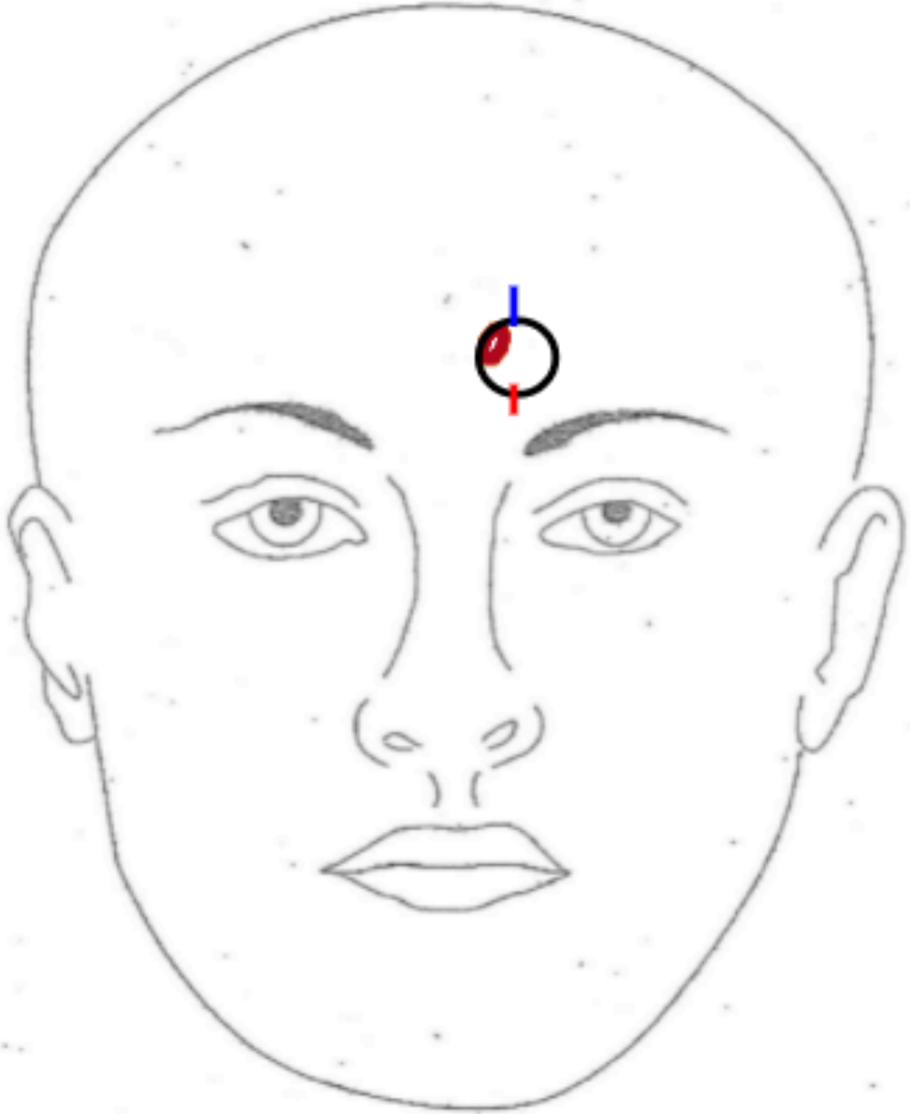

Initial size (cm)	Final Defect (cm)	Room #
1.0x1.3 cm	1.8x1.5 cm	

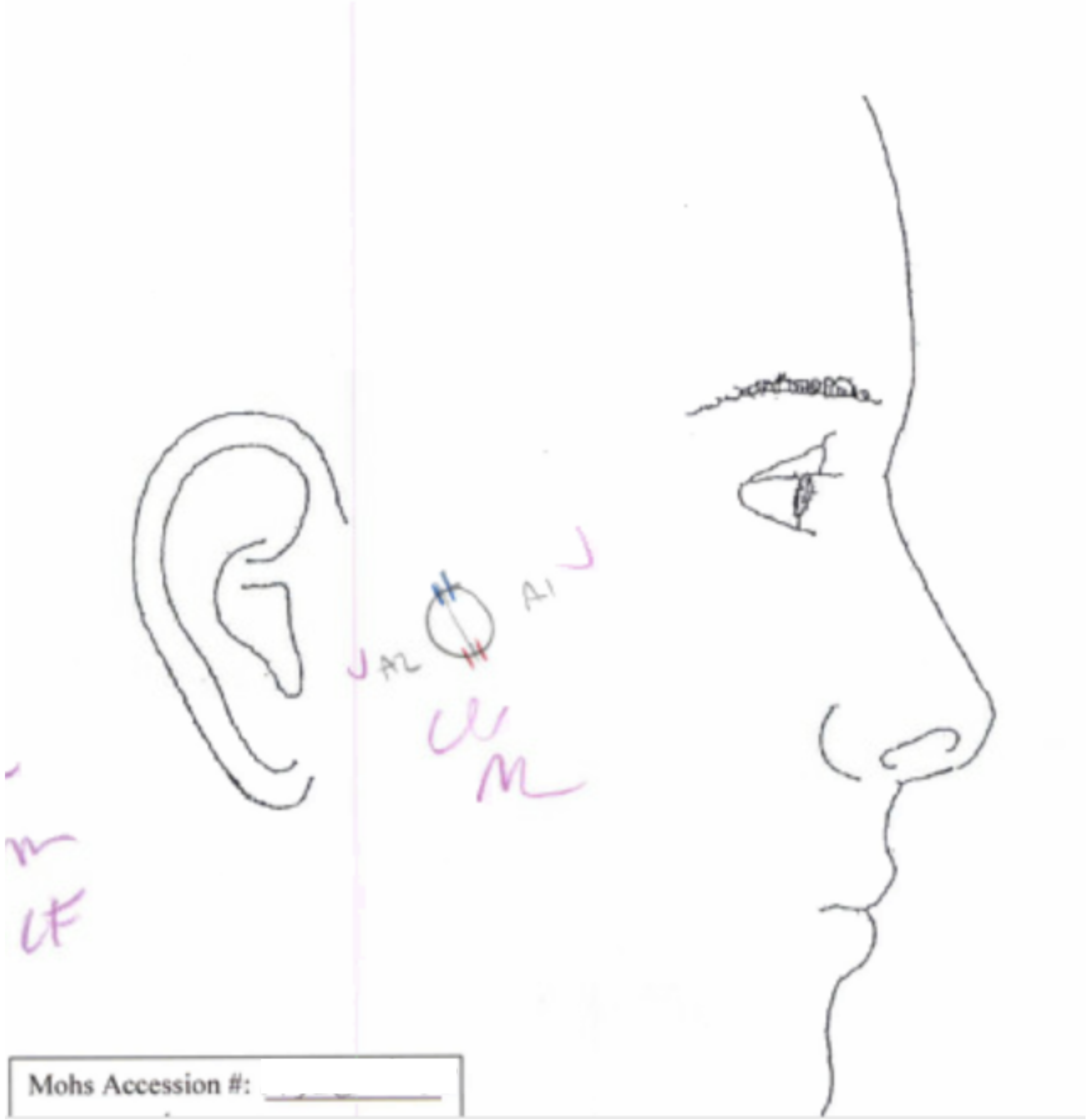

Mohs Accession #: ____________

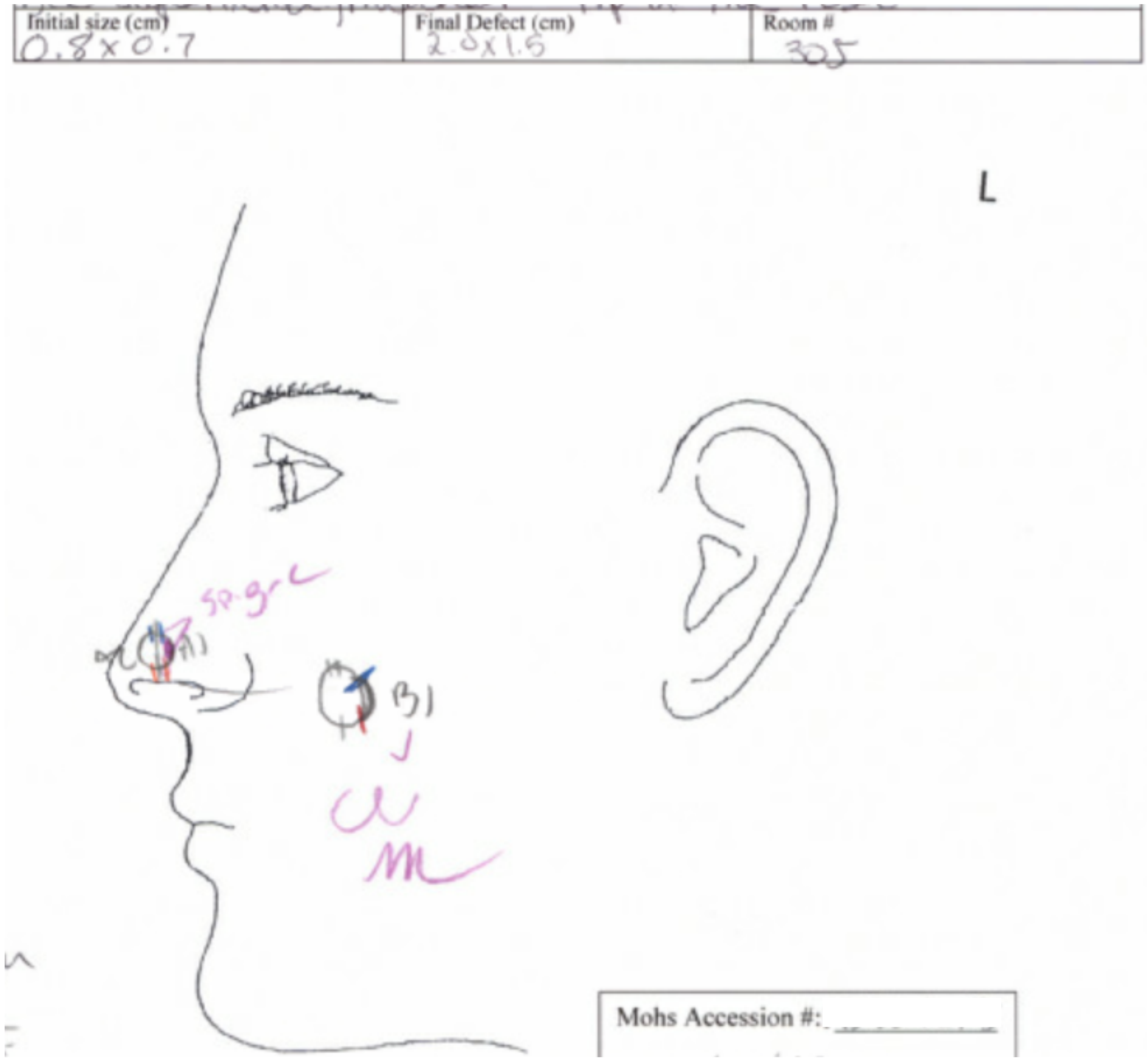

Initial size (cm)	Final Defect (cm)	Room #
0.8x0.7	2.0x1.5	305

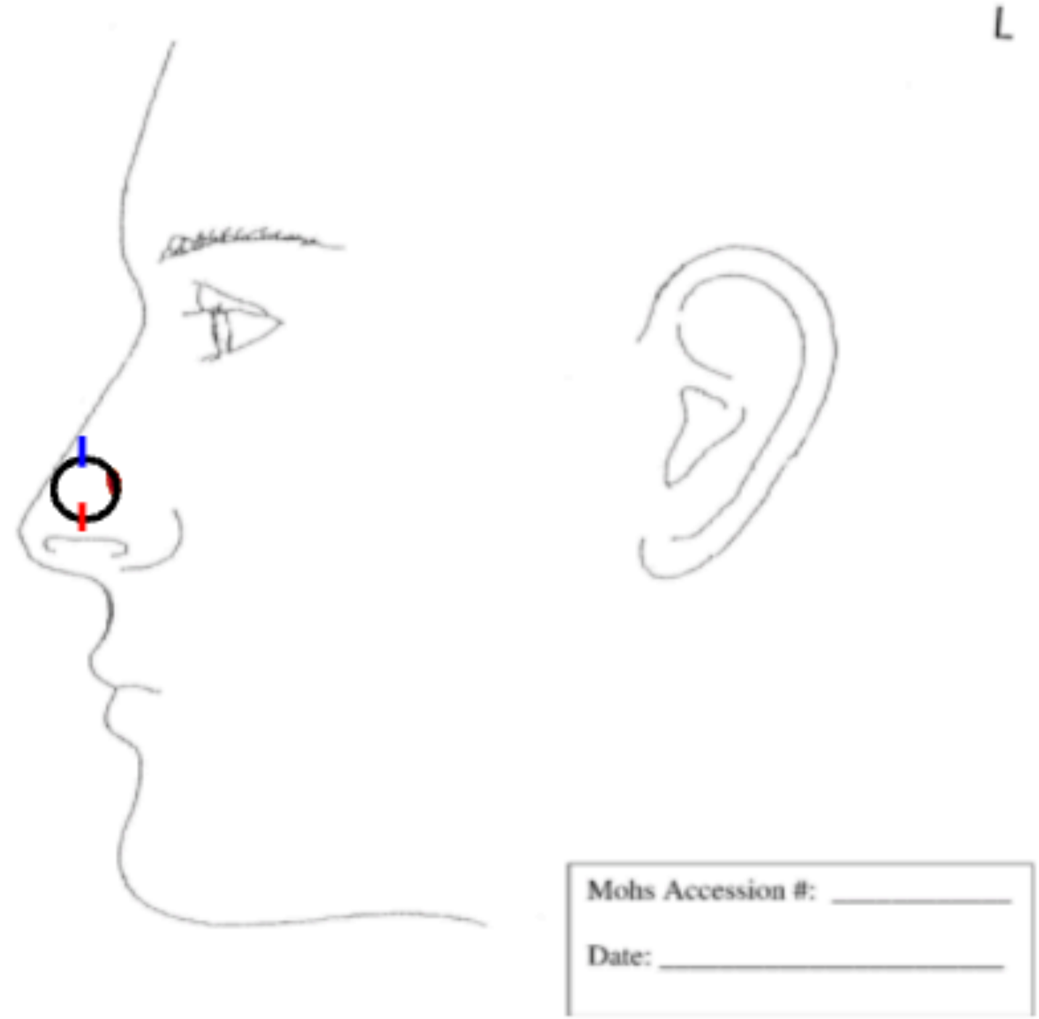

Initial size (cm)	Final Defect (cm)	Room #
0.8 x 1.4 cm	2.6 x 1.7 cm (B)	303

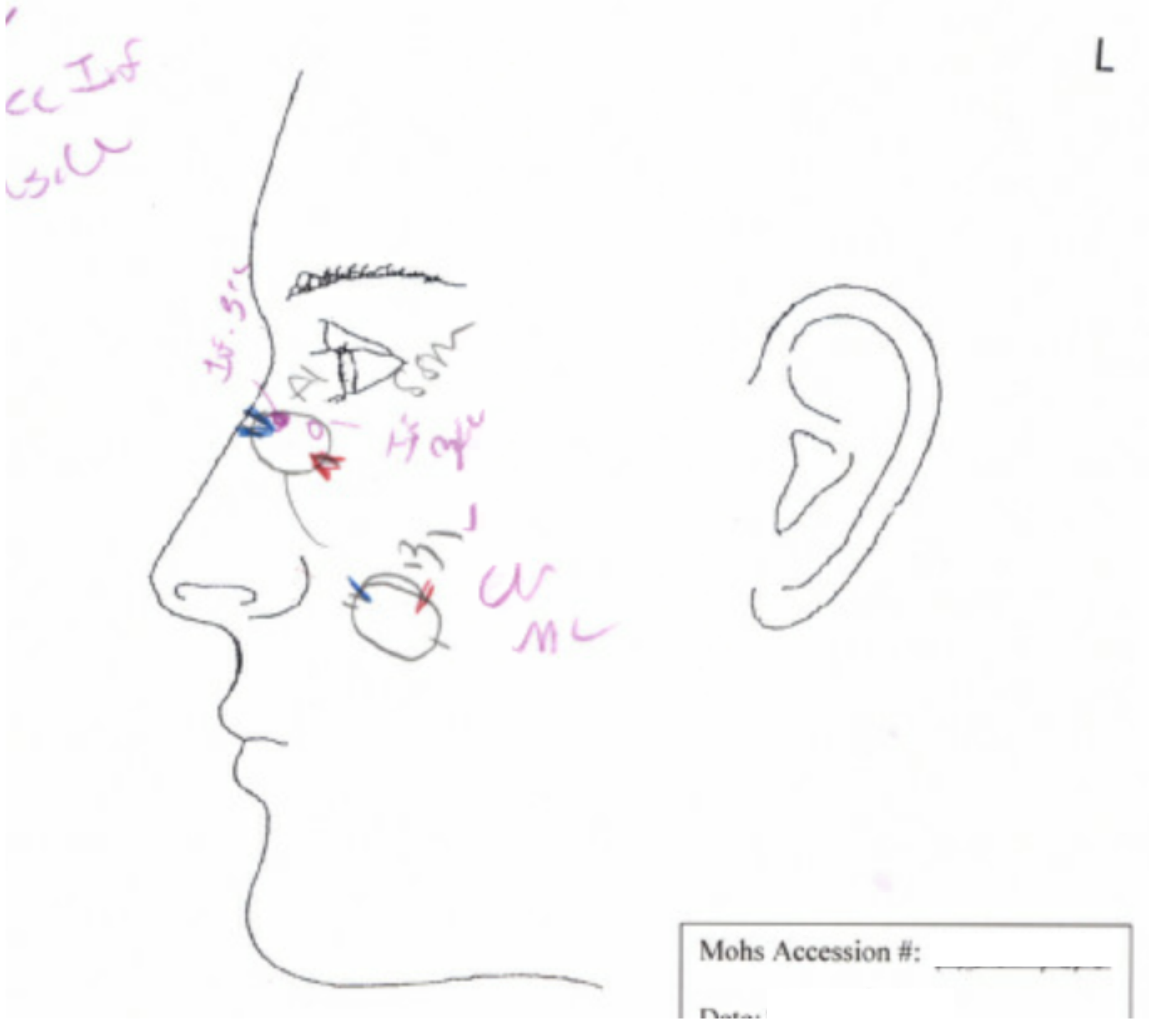

Mohs Accession #:

Date:

A1

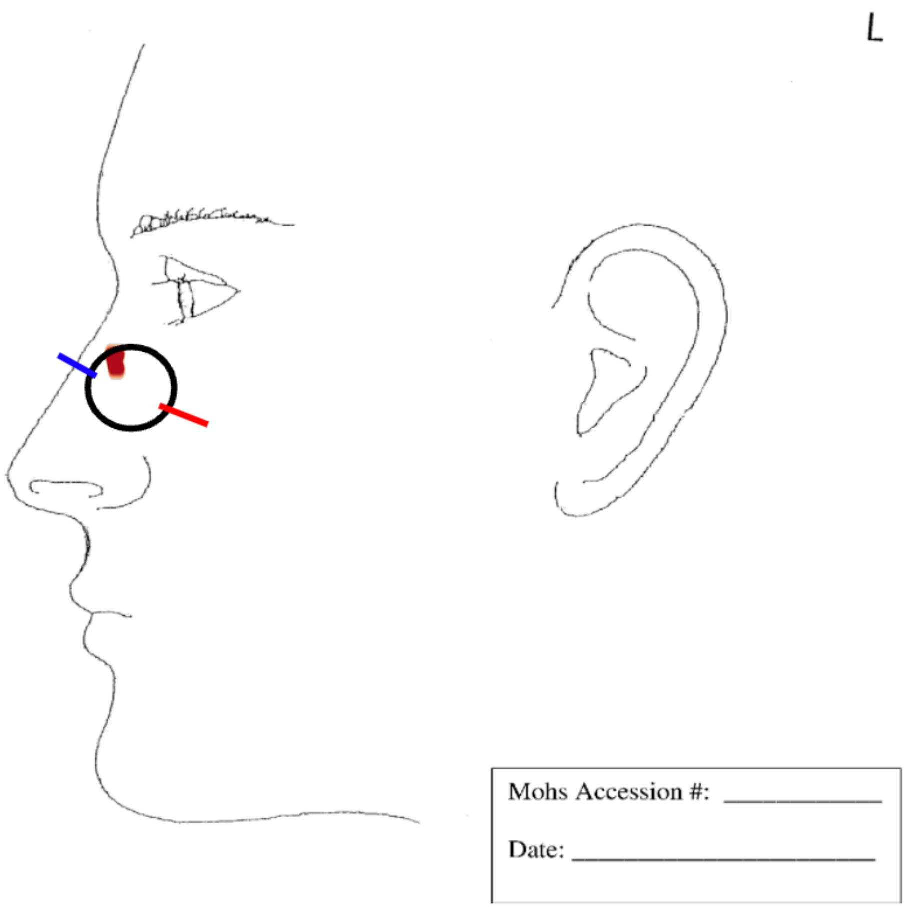

Mohs Accession #: ____________

Date: ______________________

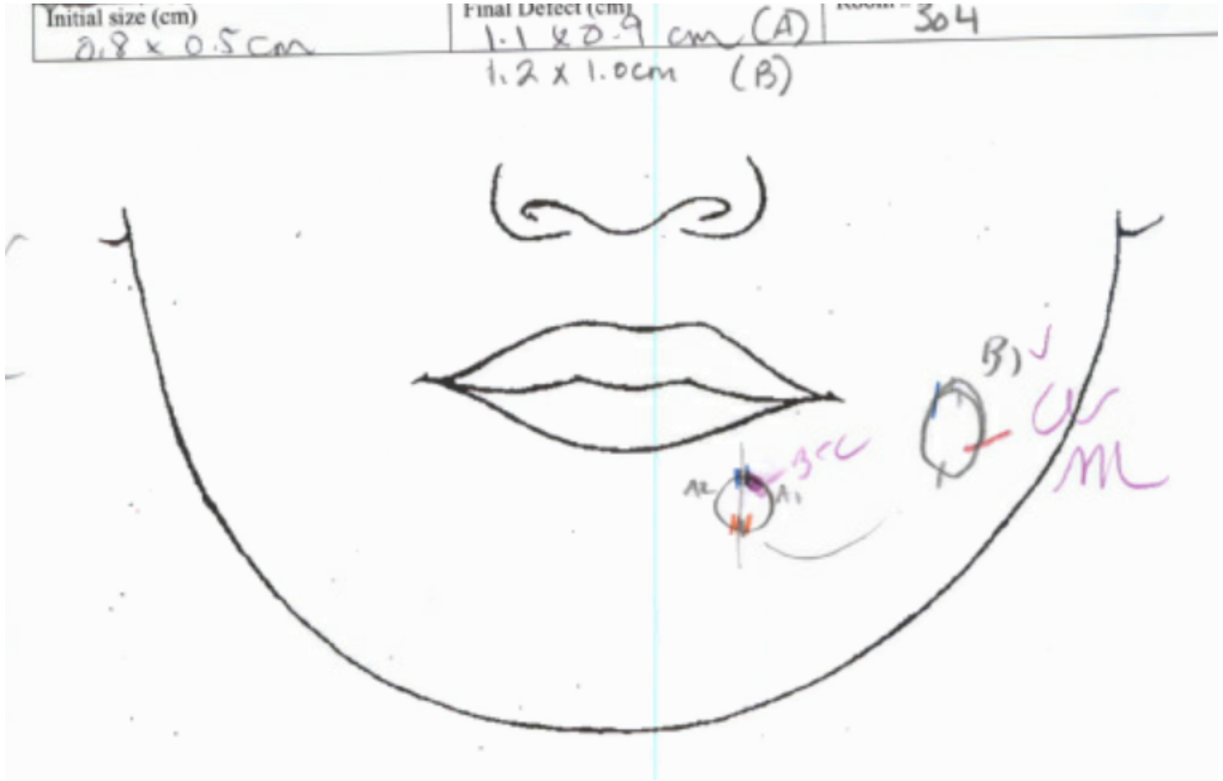
Initial size (cm)
0.8 x 0.5 cm
Final Defect (cm)
1.1 x 0.9 cm (A)
1.2 x 1.0 cm (B)
304
B)
A2
A1

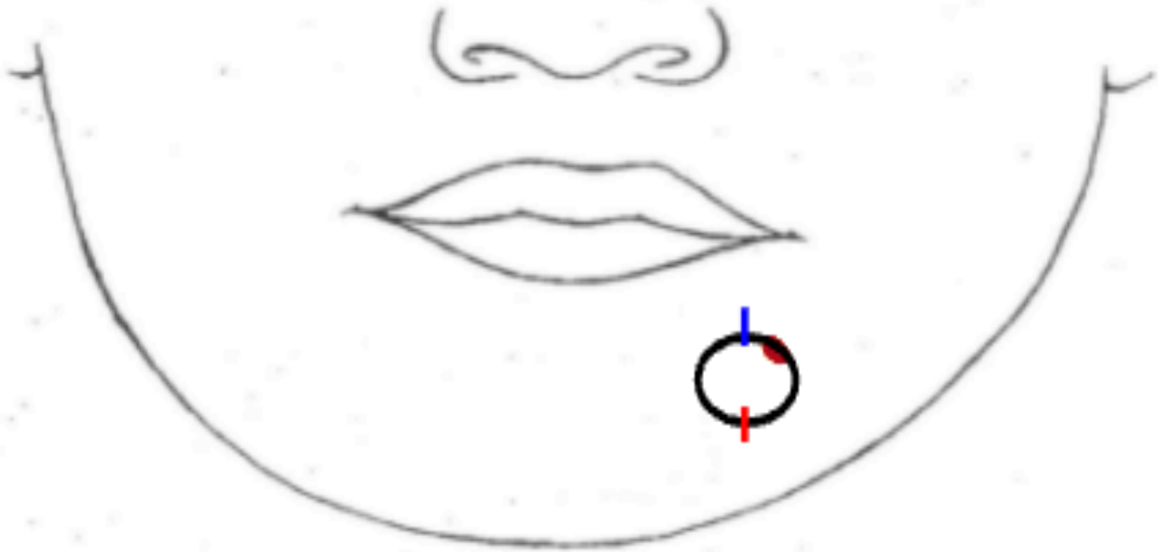

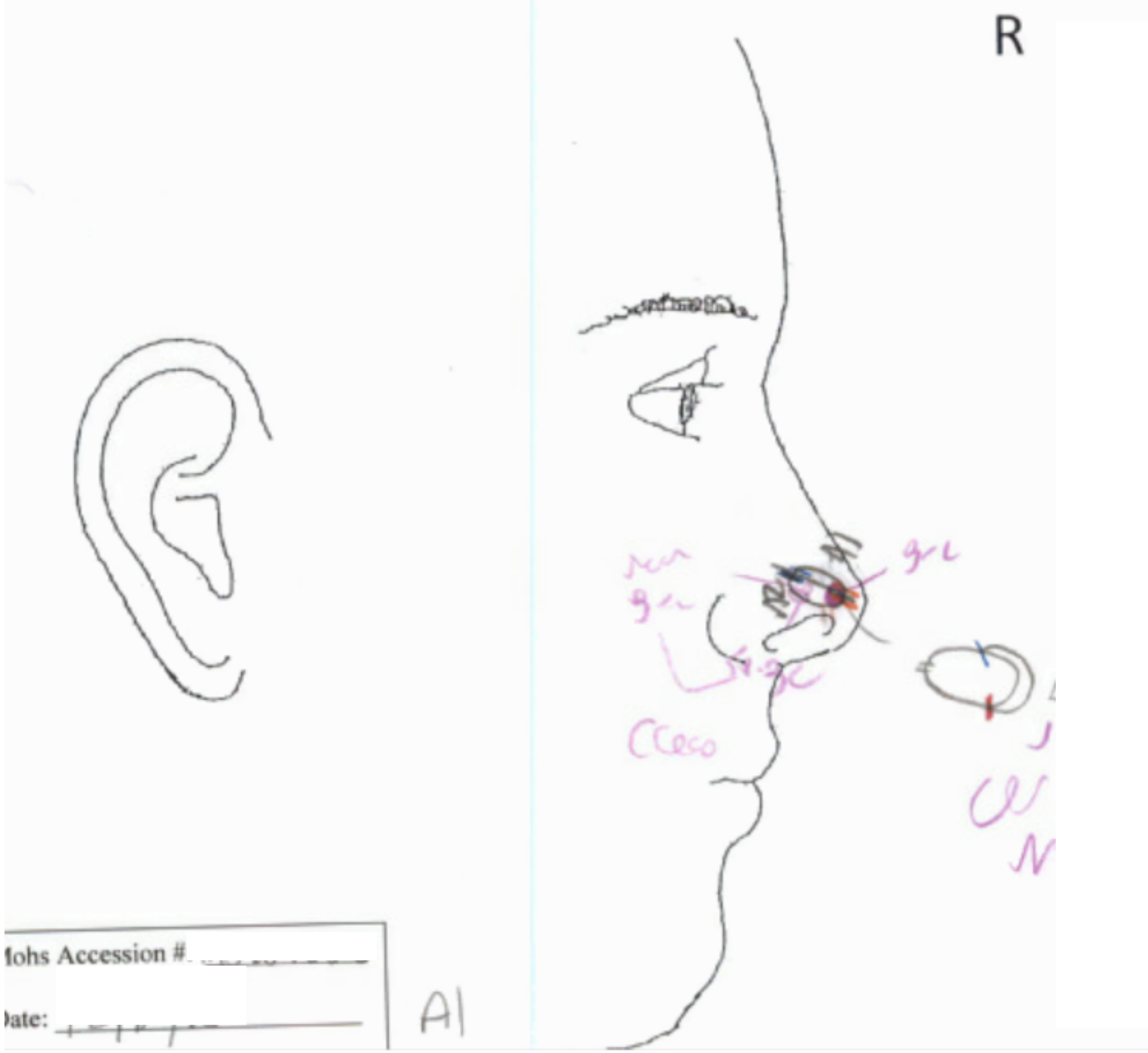
R
Mohs Accession #
Date:
A1

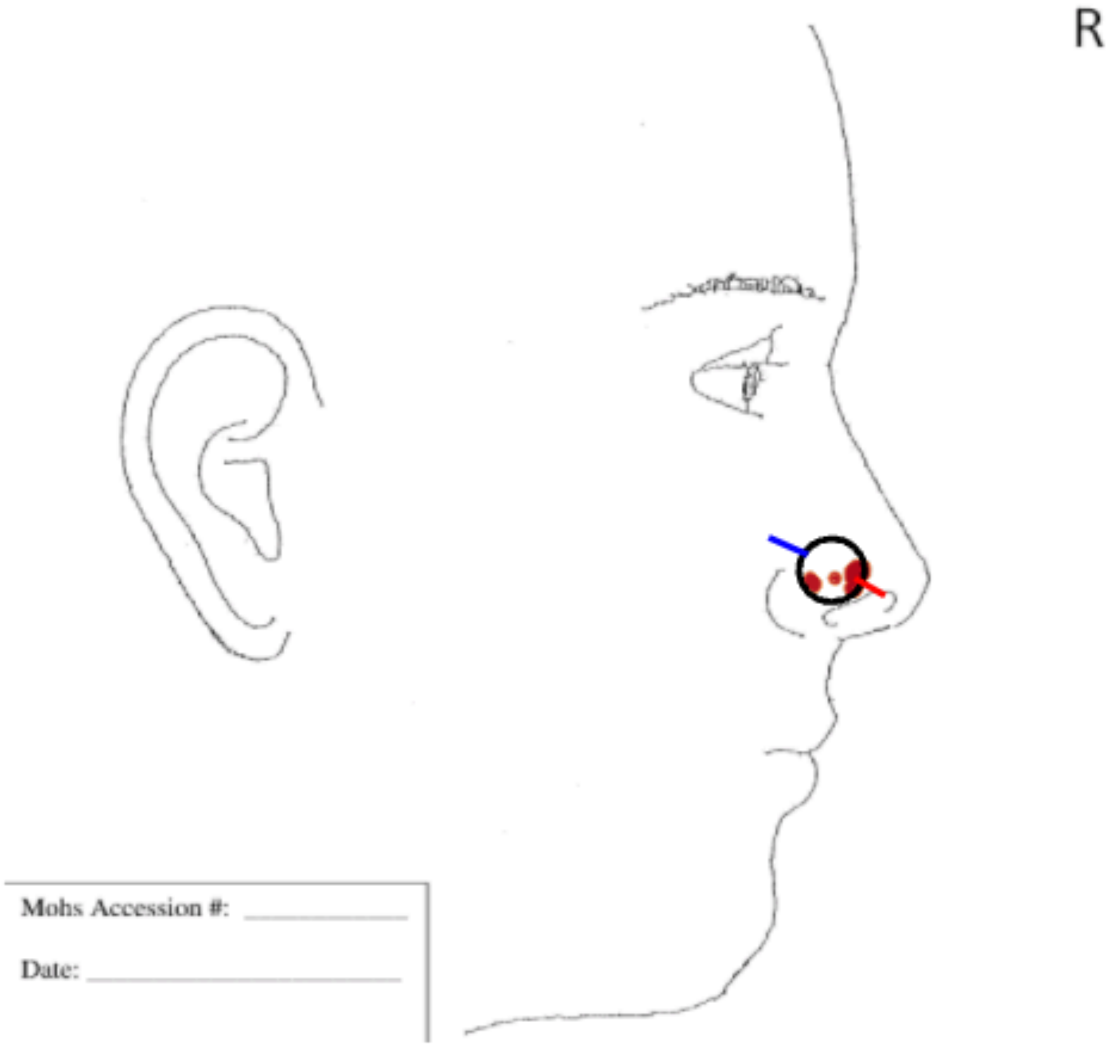
R
Mohs Accession #:
Date:

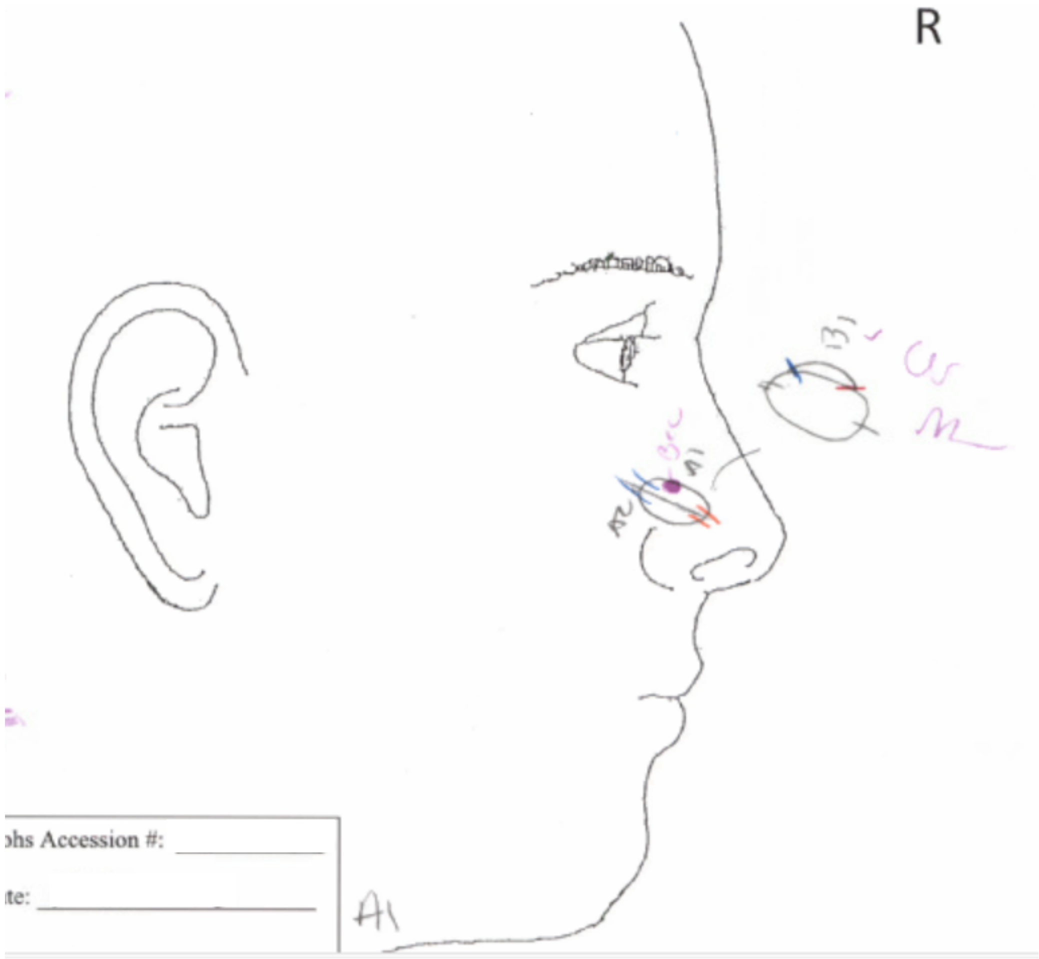
R
hs Accession #:
te:
A1

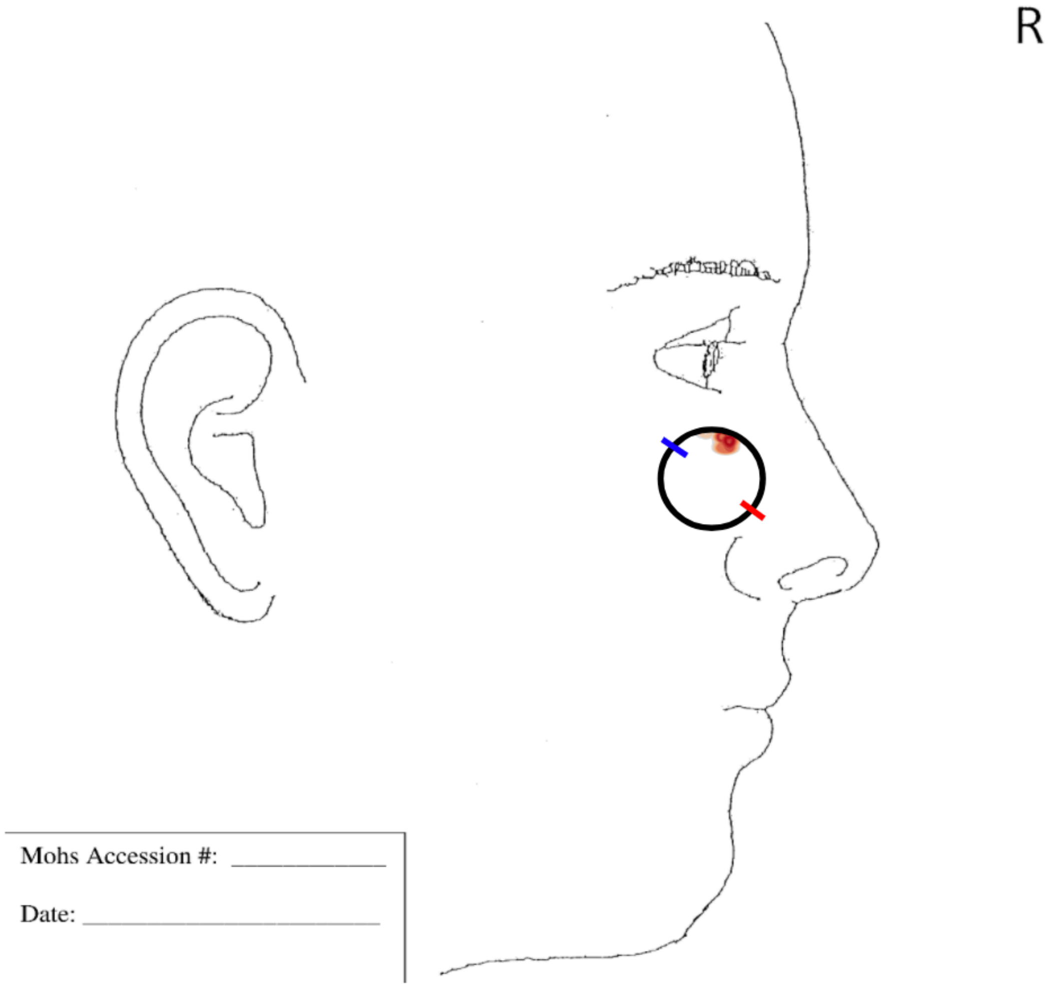
R
Mohs Accession #:
Date:

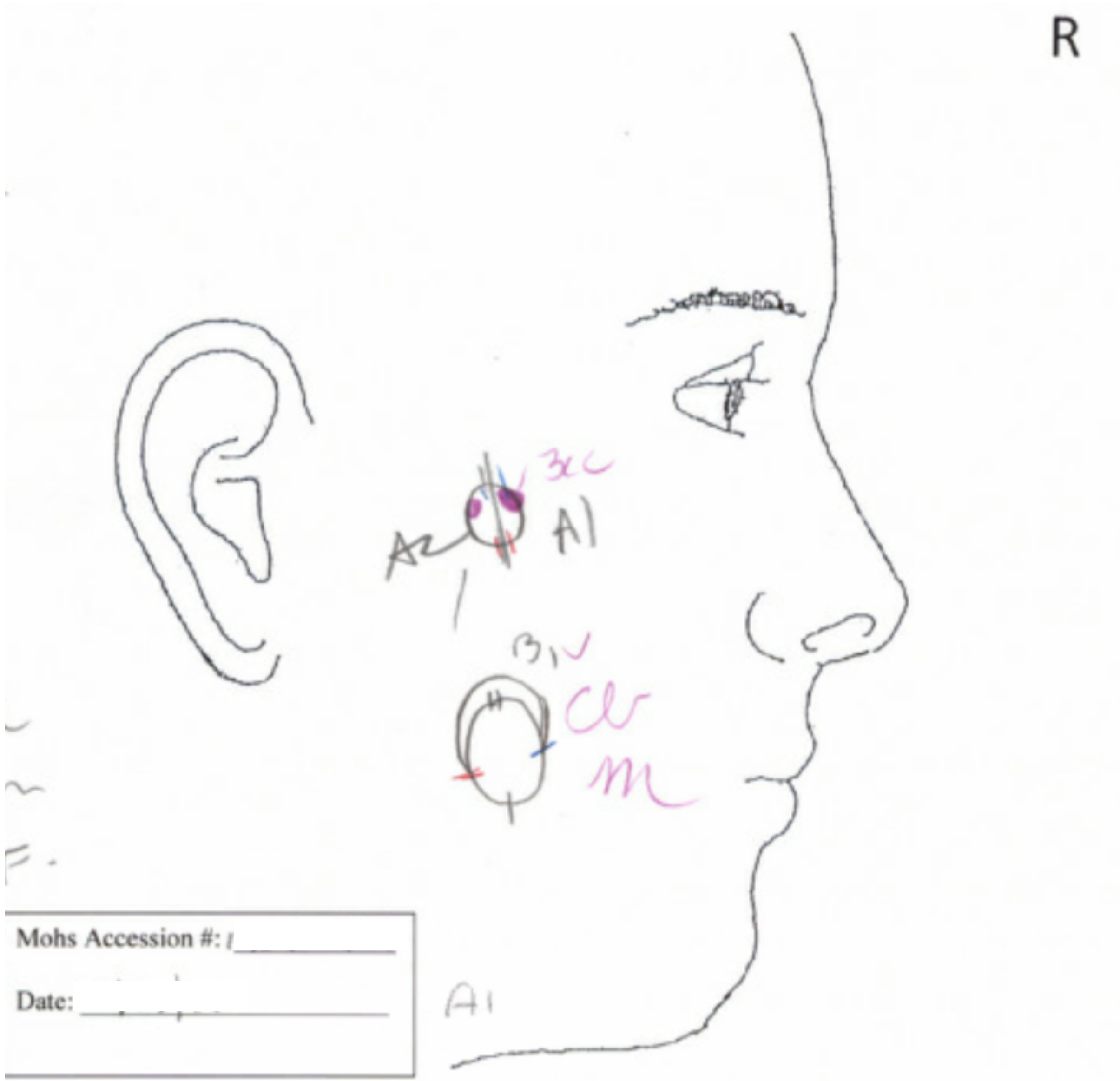
R
A2
A1
Mohs Accession #:
Date:
A1

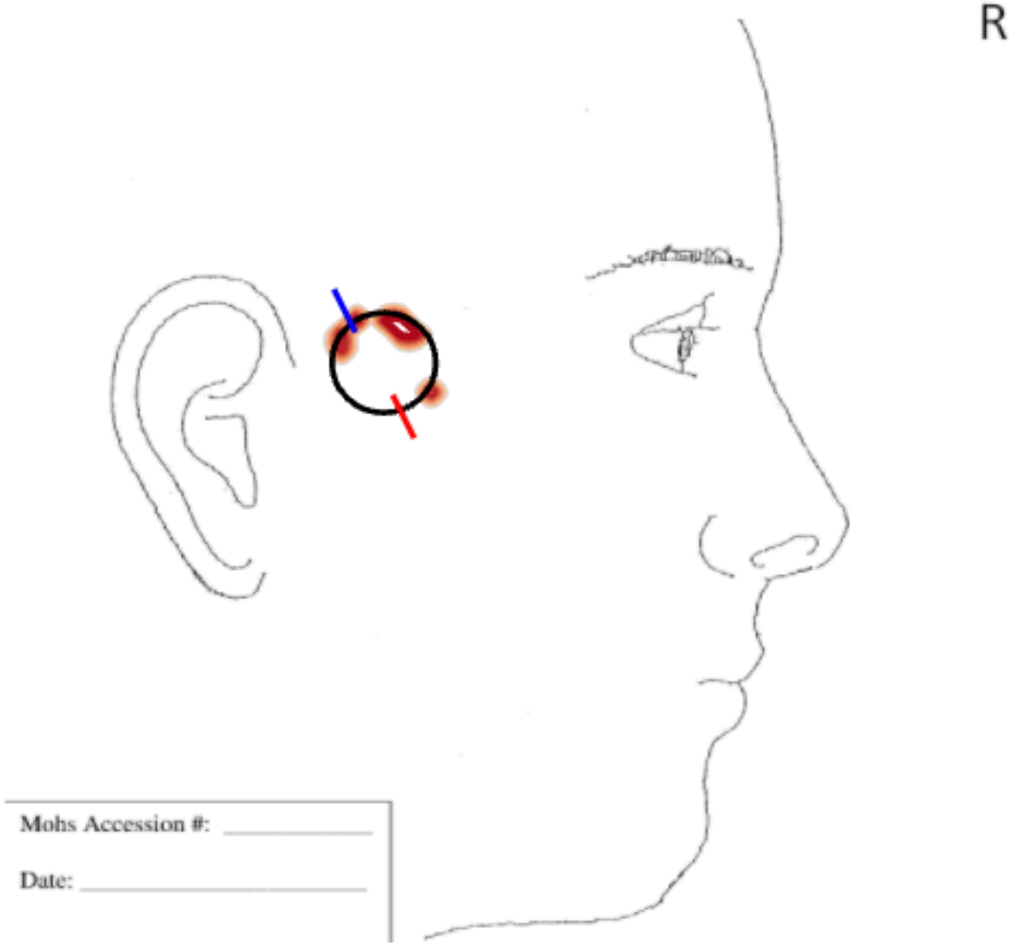
R
Mohs Accession #:
Date:

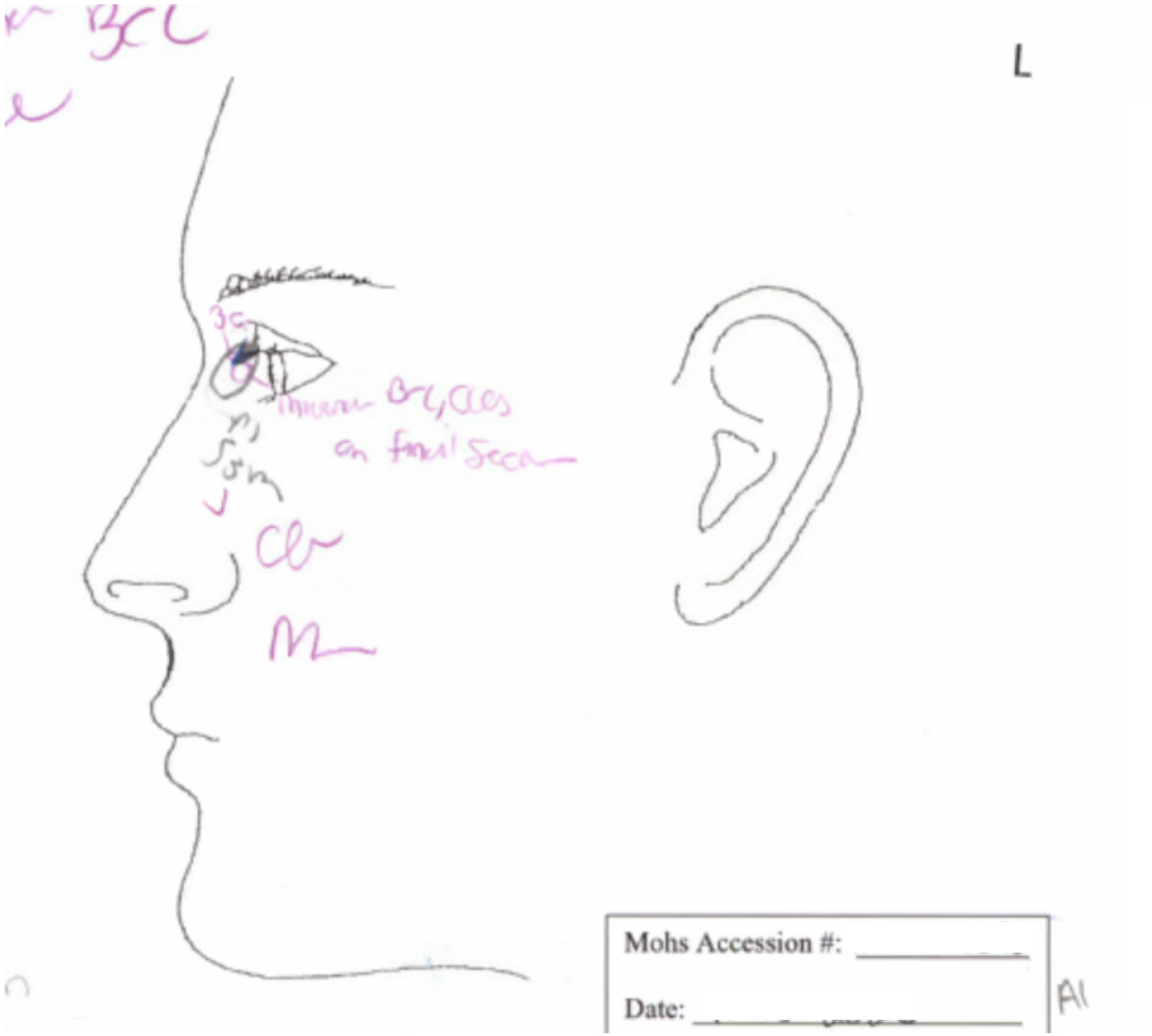
BCC
L
Mohs Accession #:
Date:

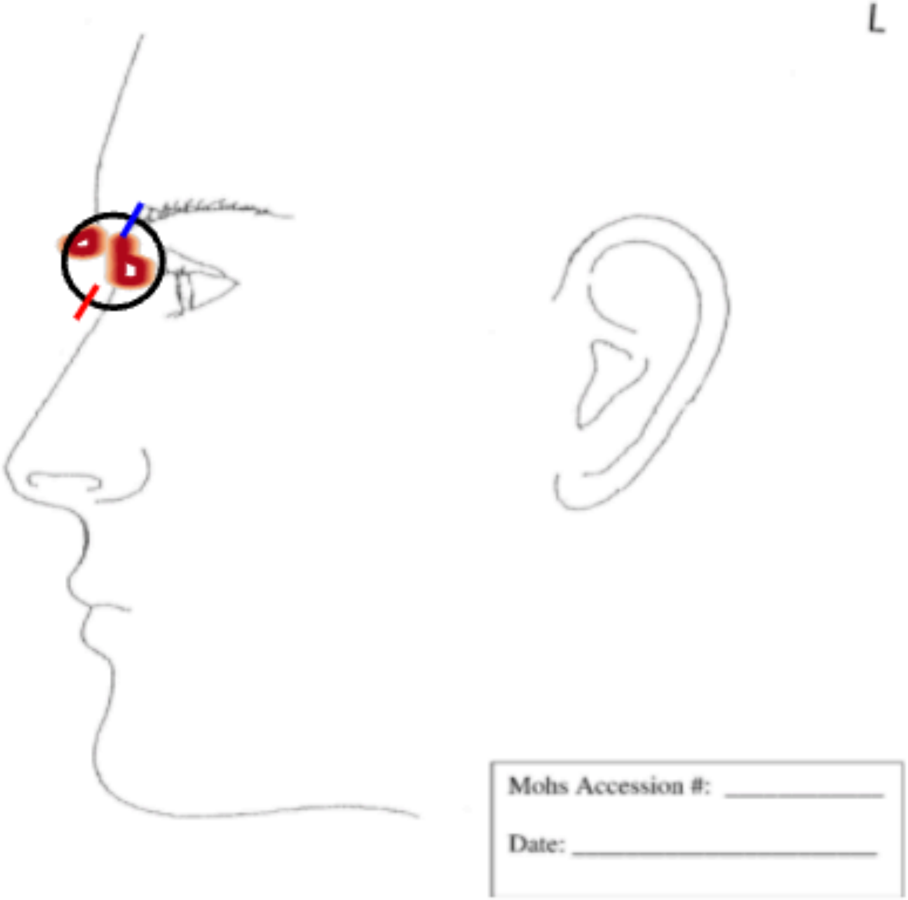
L
Mohs Accession #:
Date:

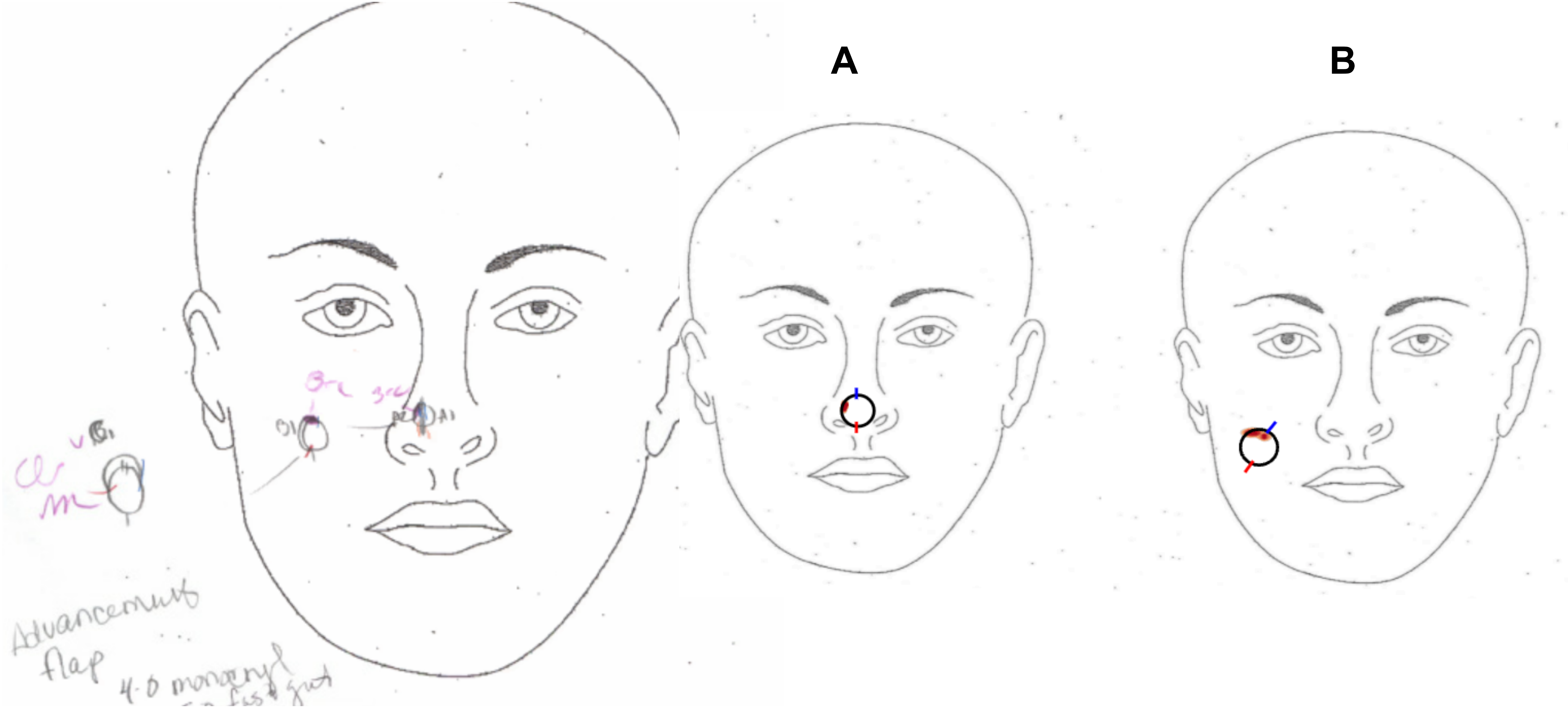

A
B
Advancement flap

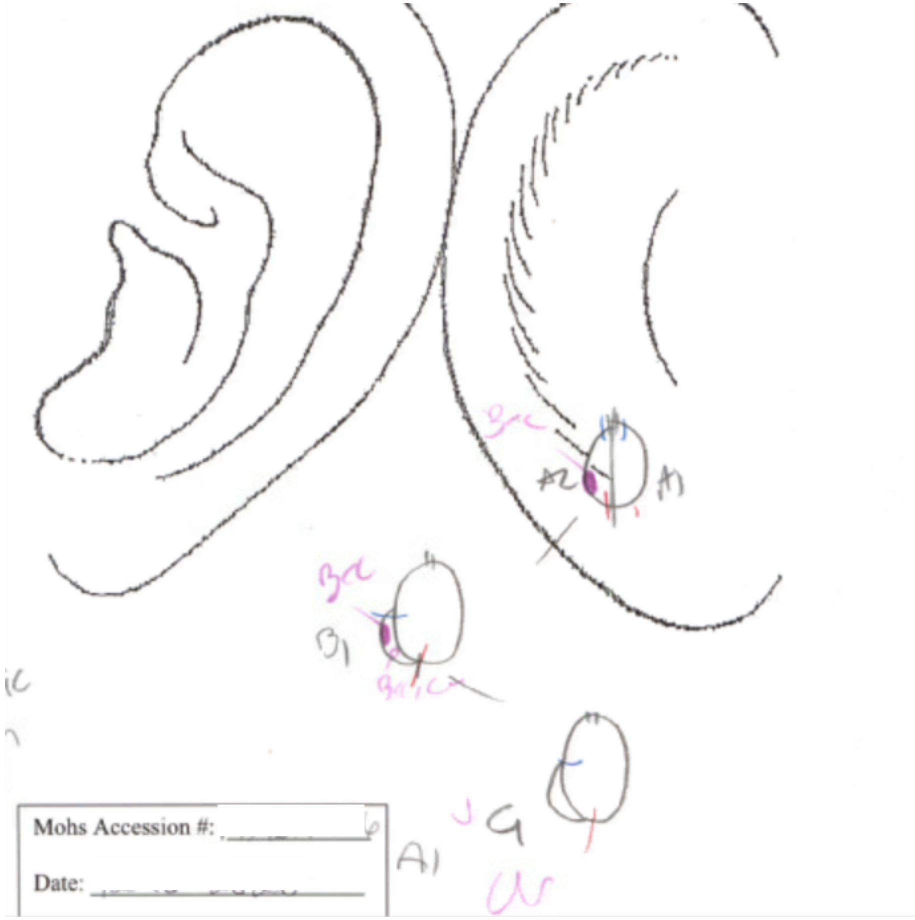

L

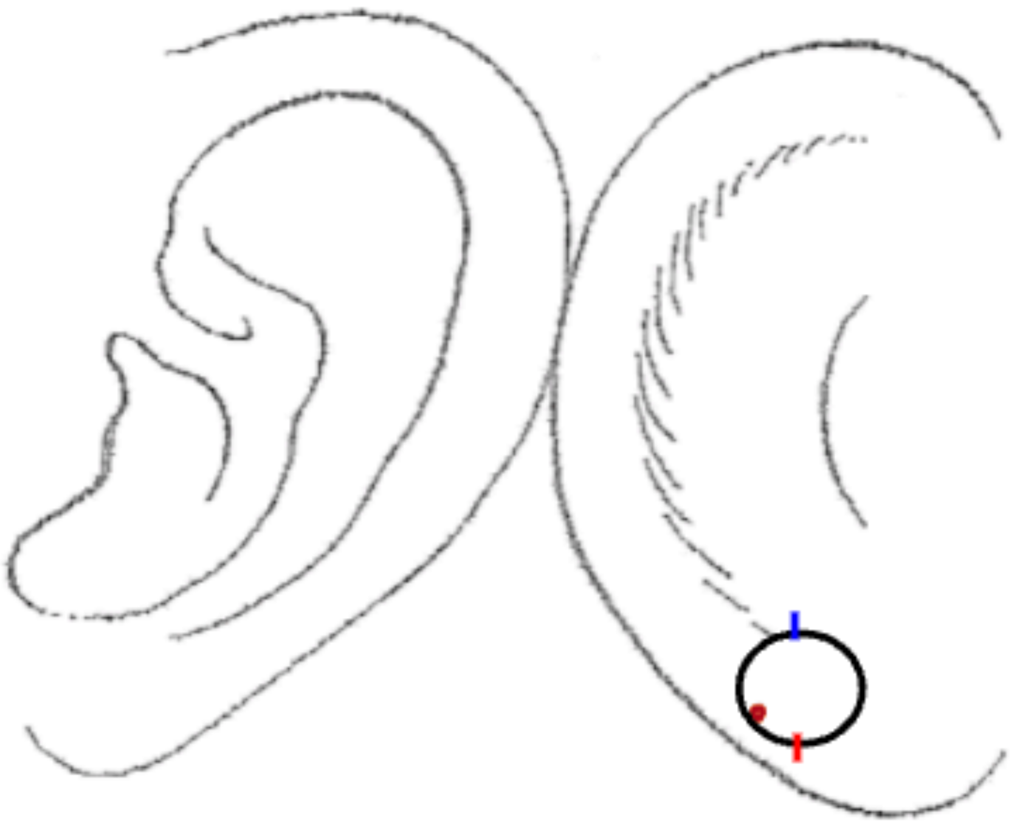

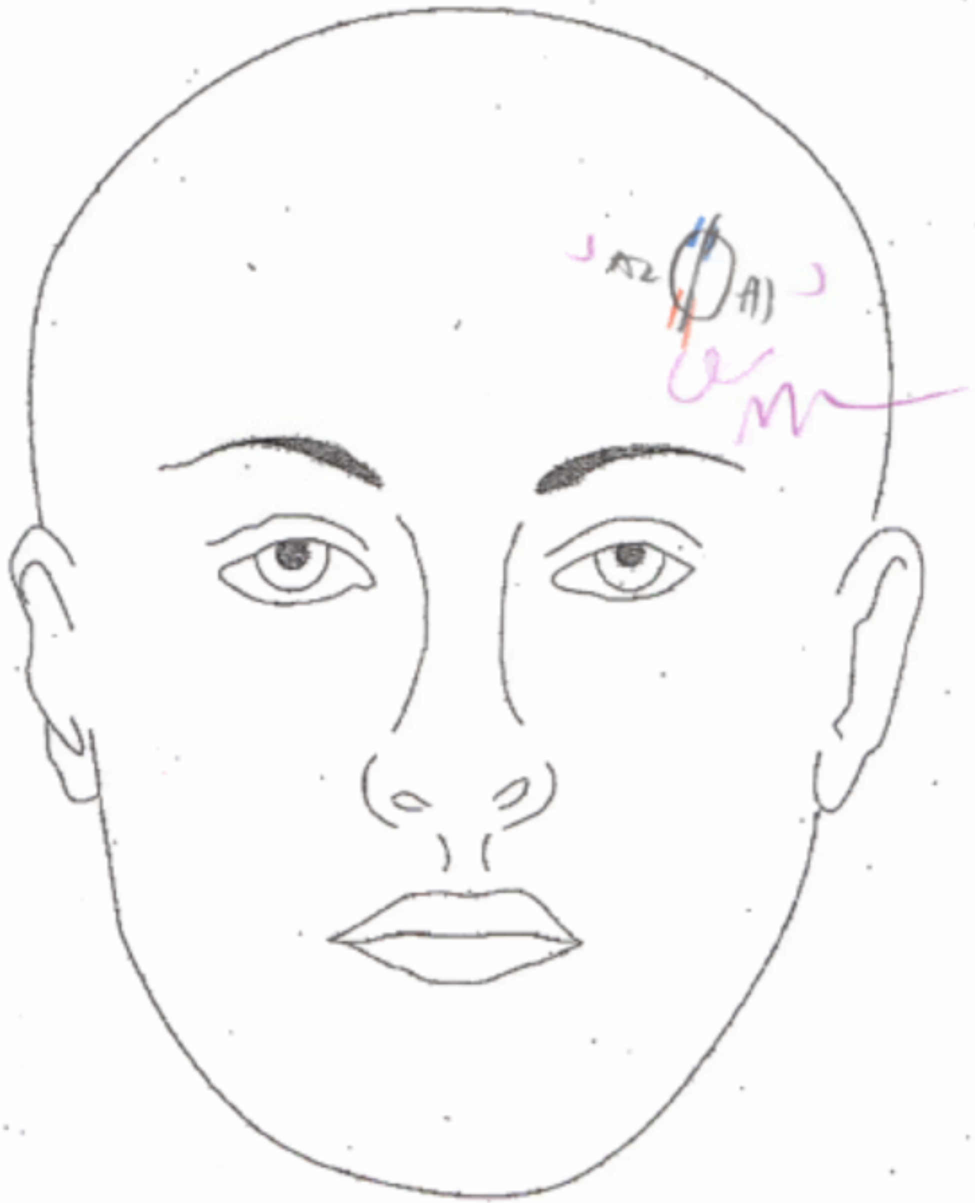
A2
A1

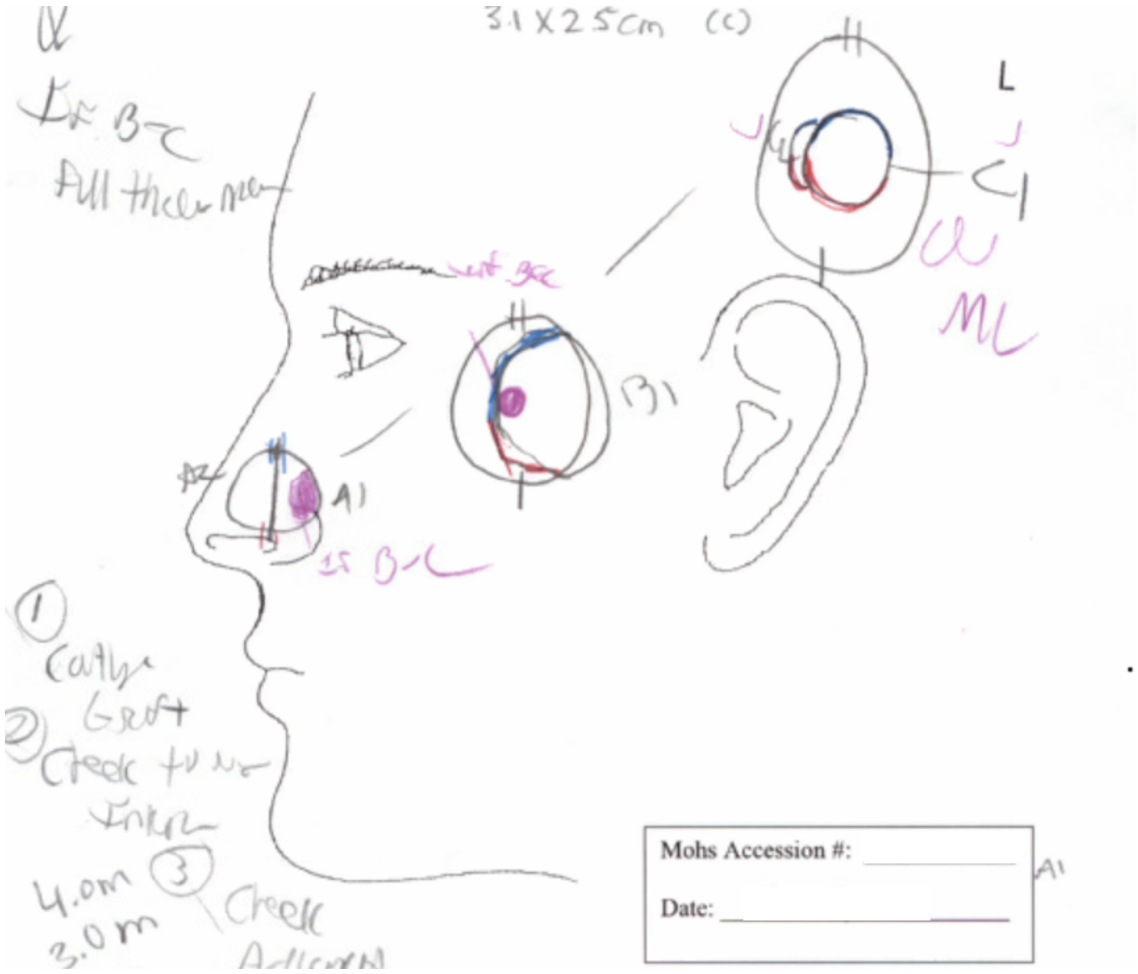

B

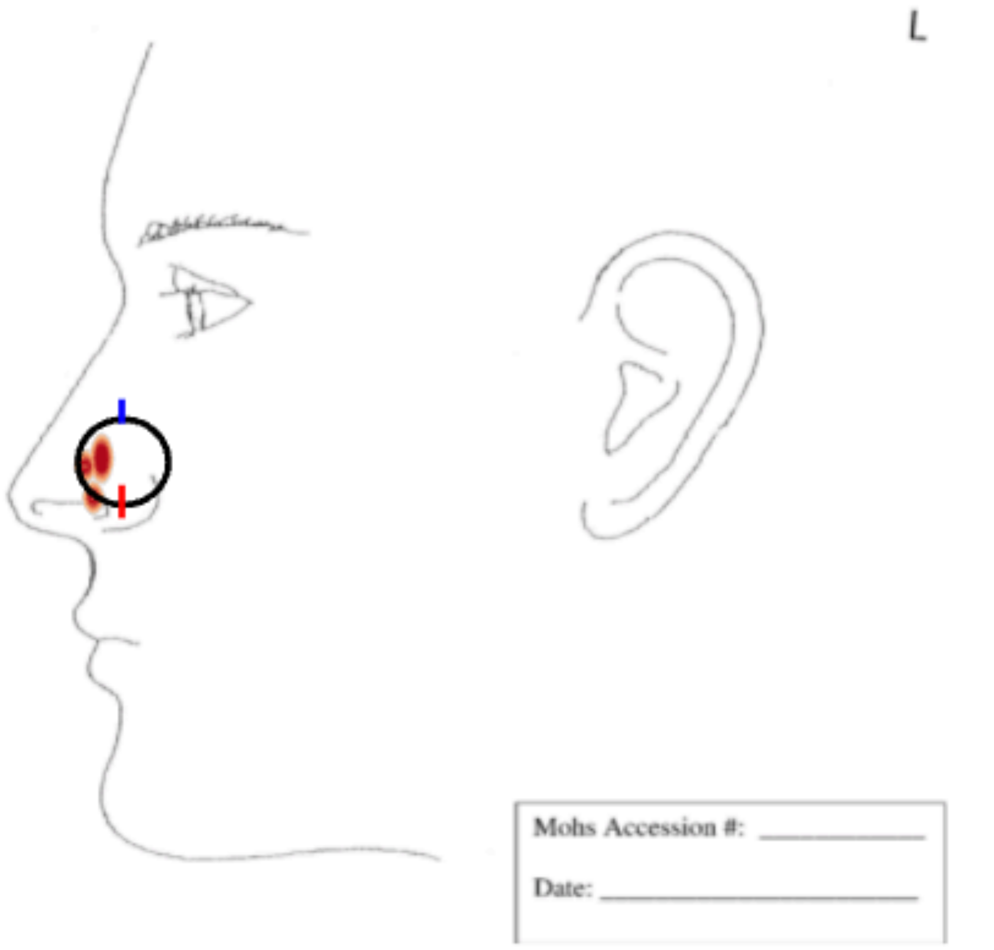

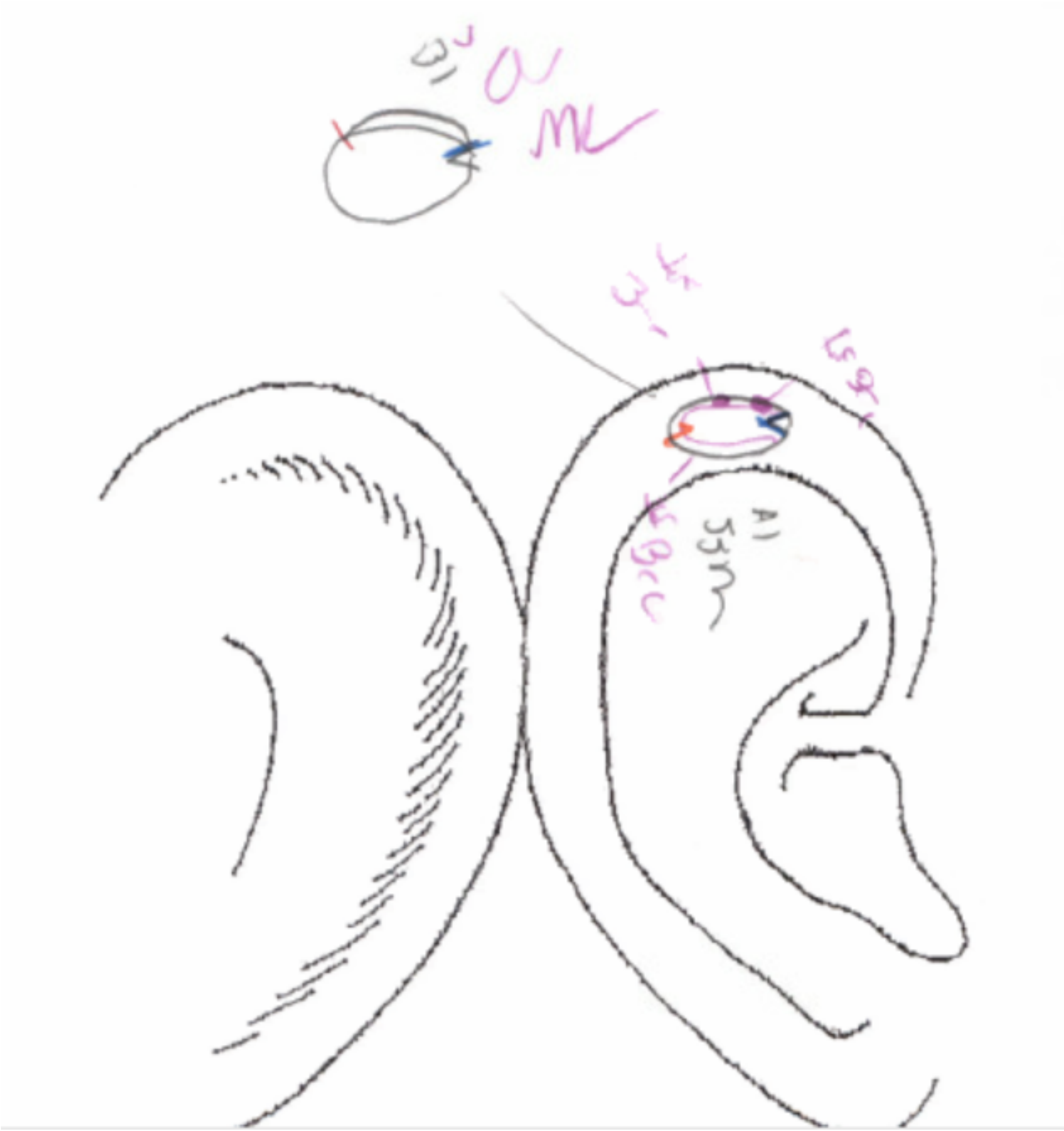

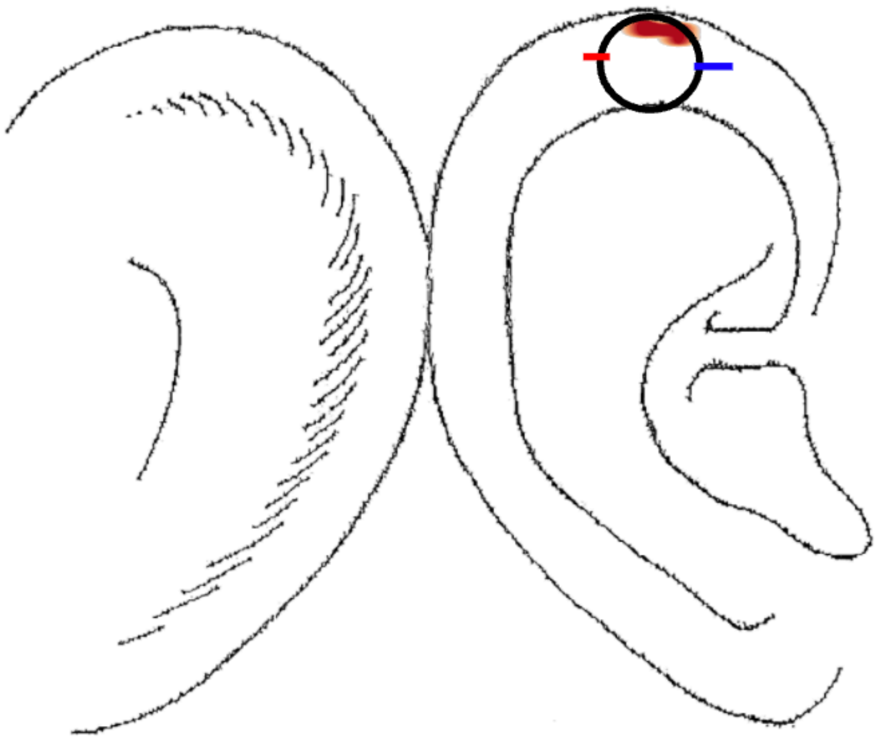

R

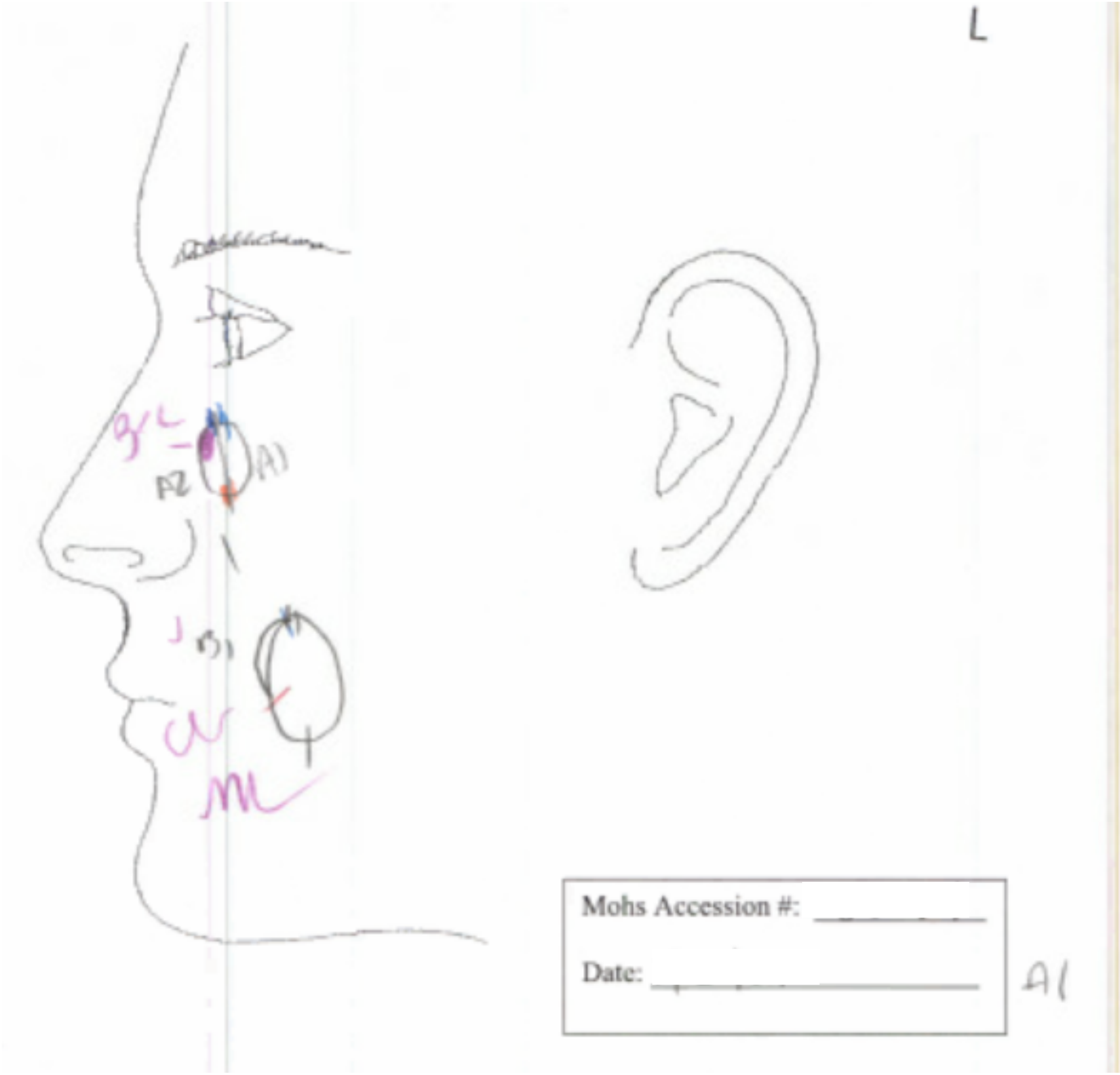
L
A2
A1
Mohs Accession #:
Date:
A1

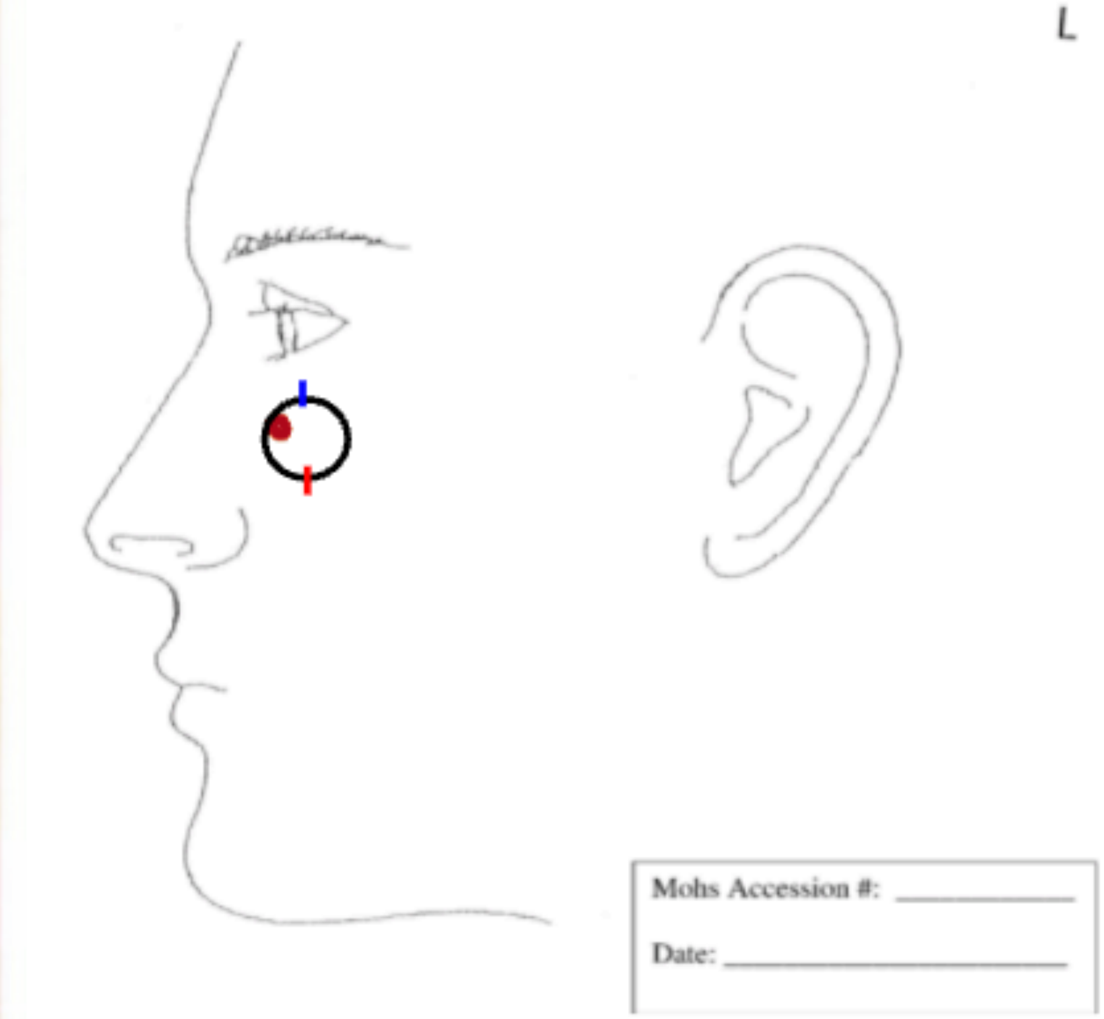
L
Mohs Accession #:
Date:

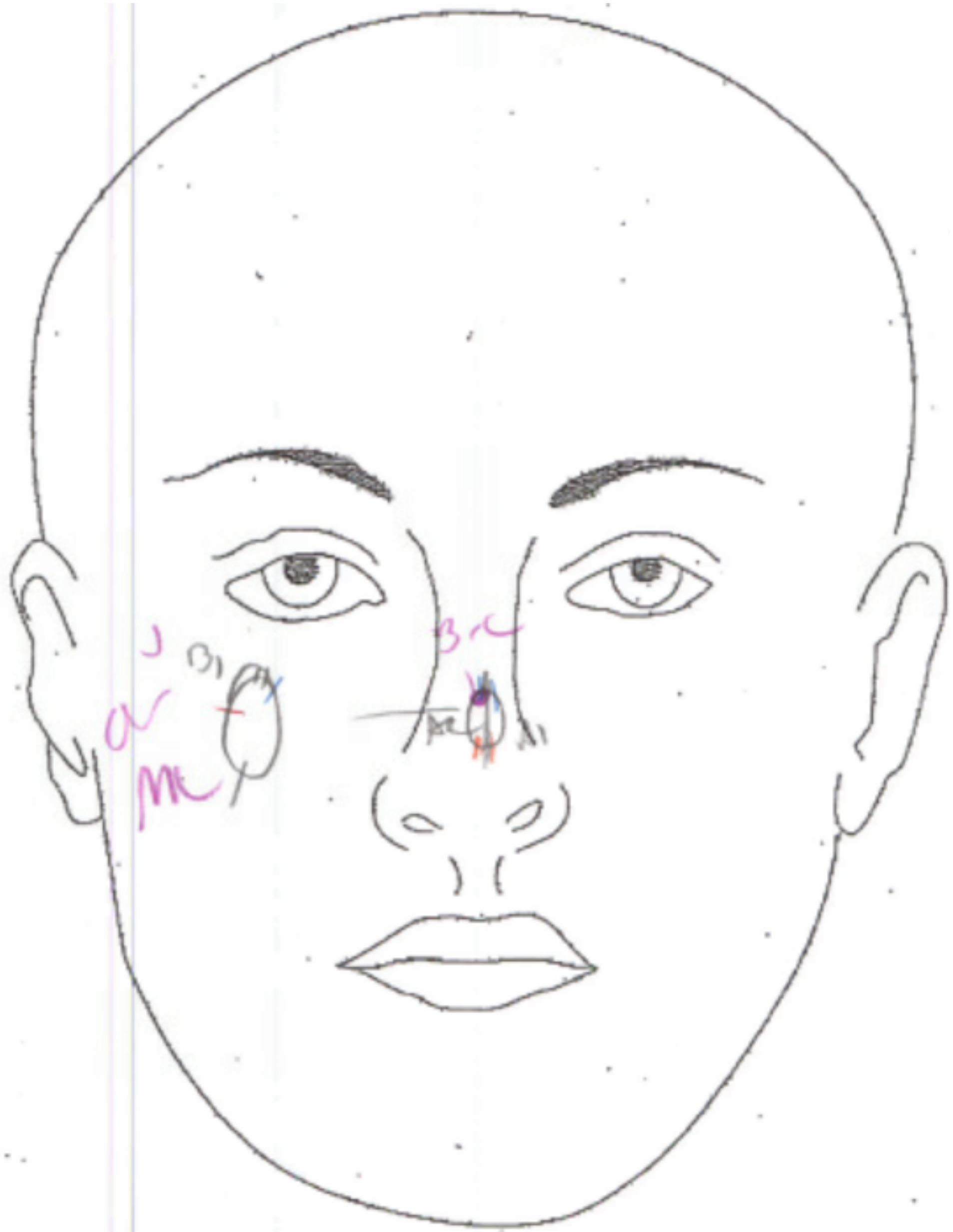

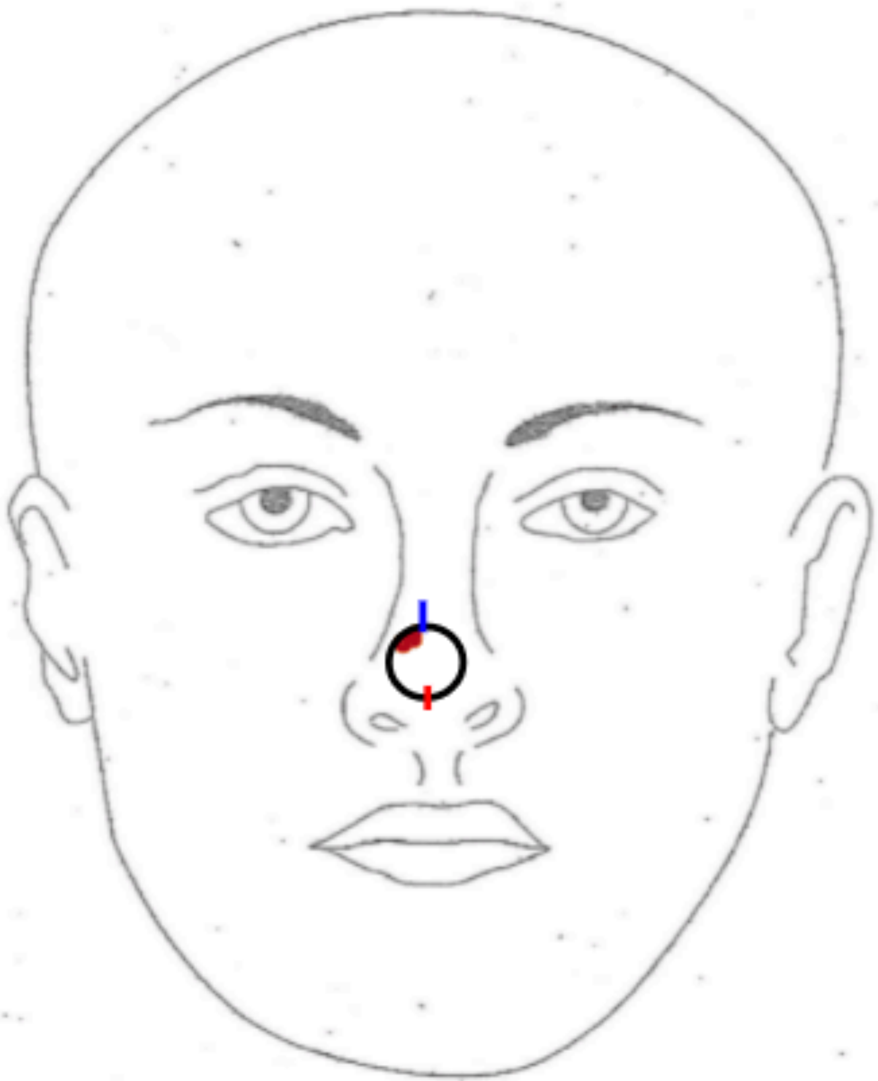

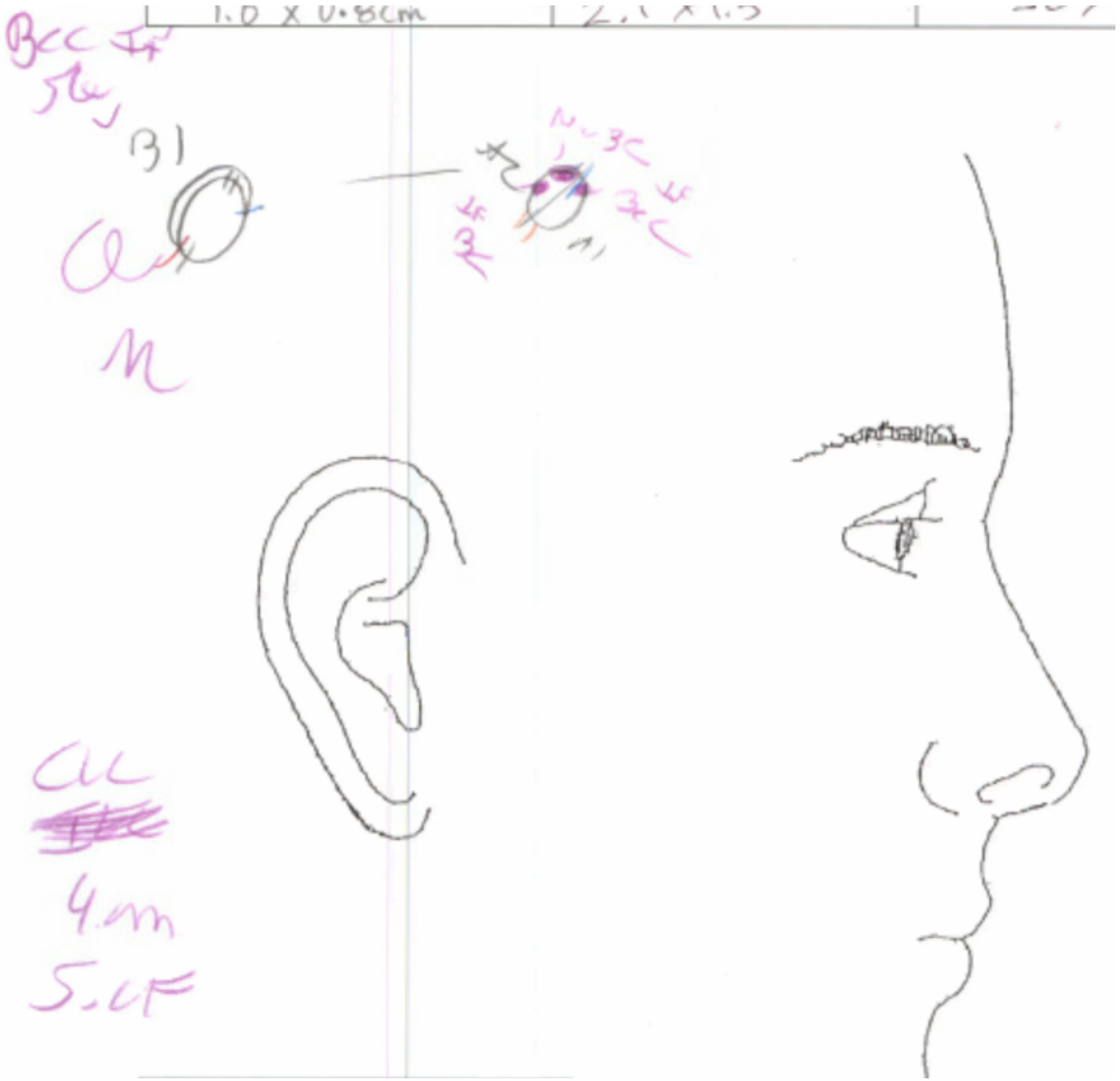

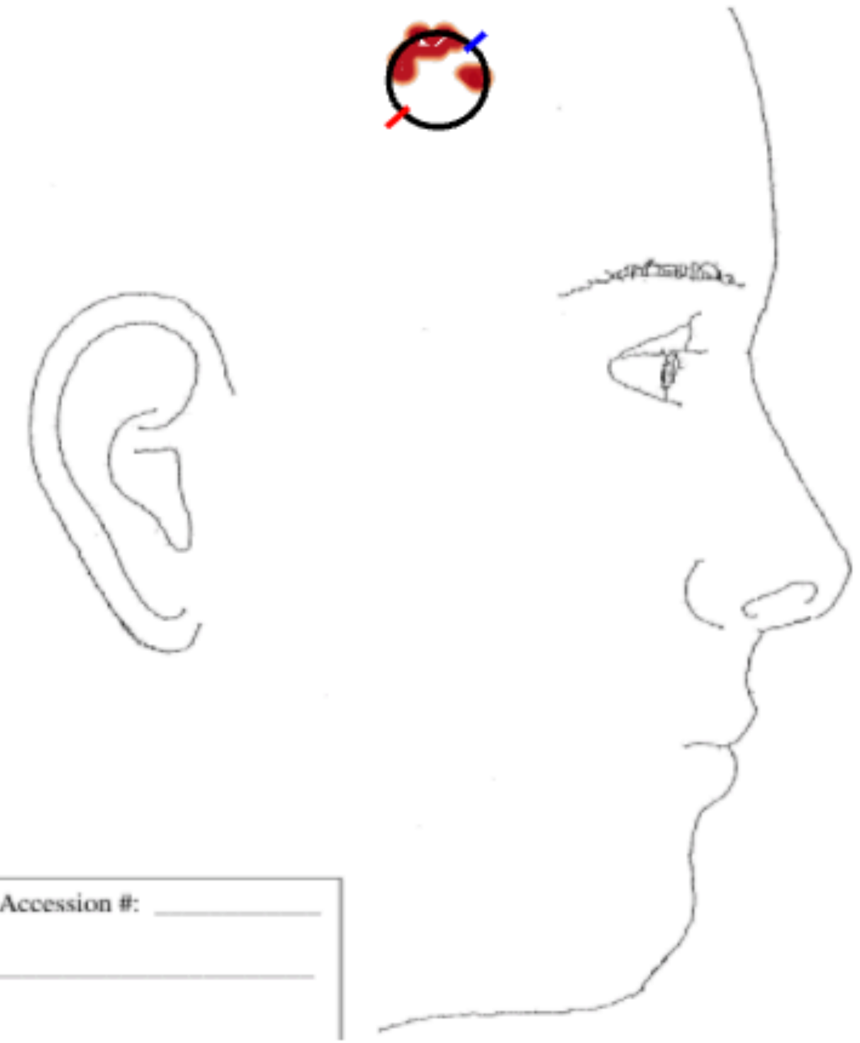

Mohs Accession #: ____________

Date: ____________

R

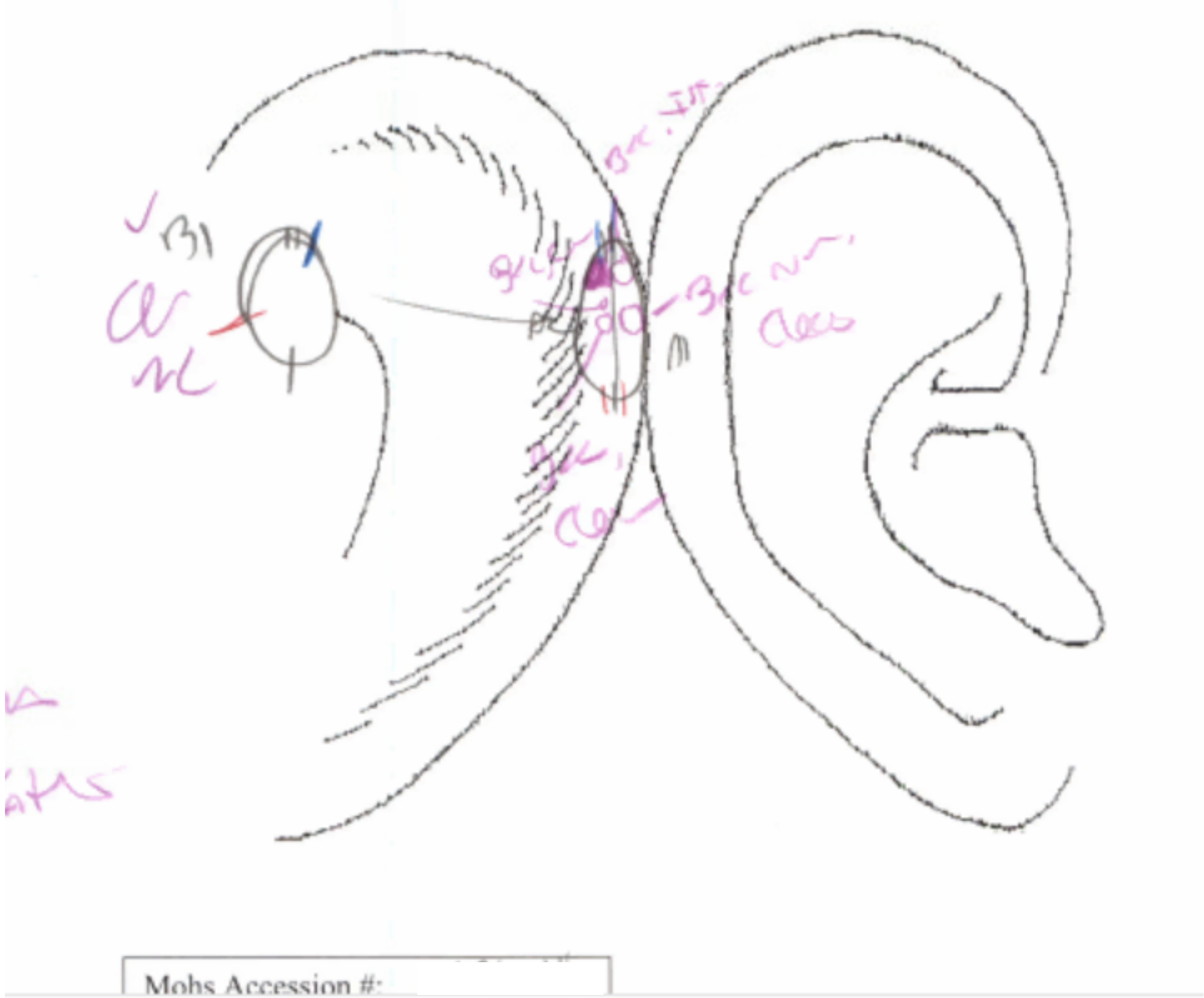

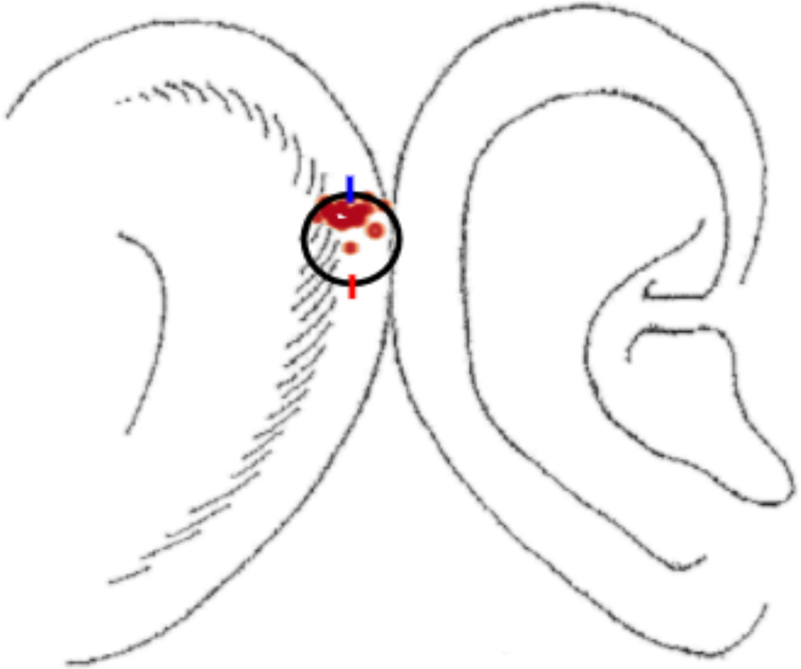

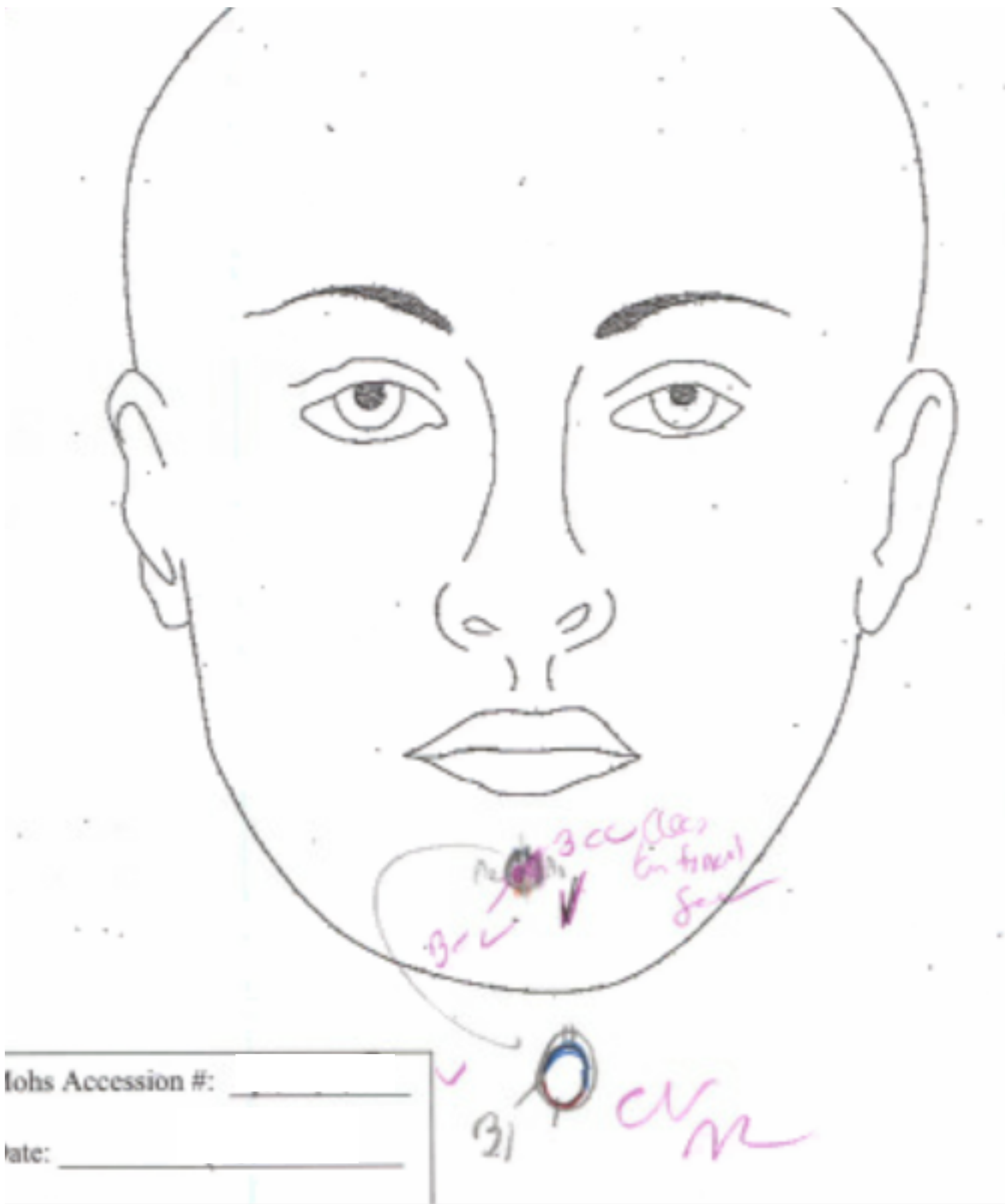

lohs Accession #: ____________

ate: ____________

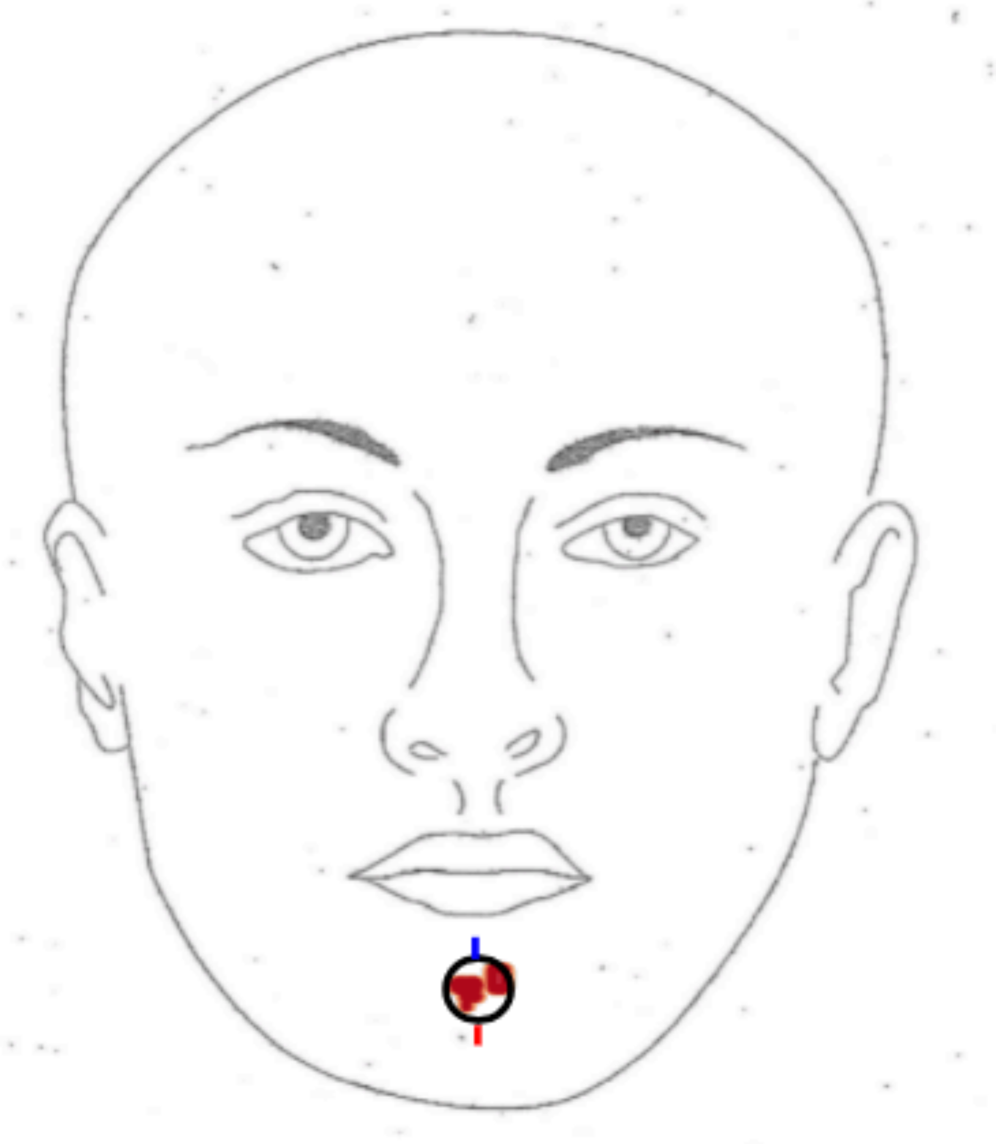

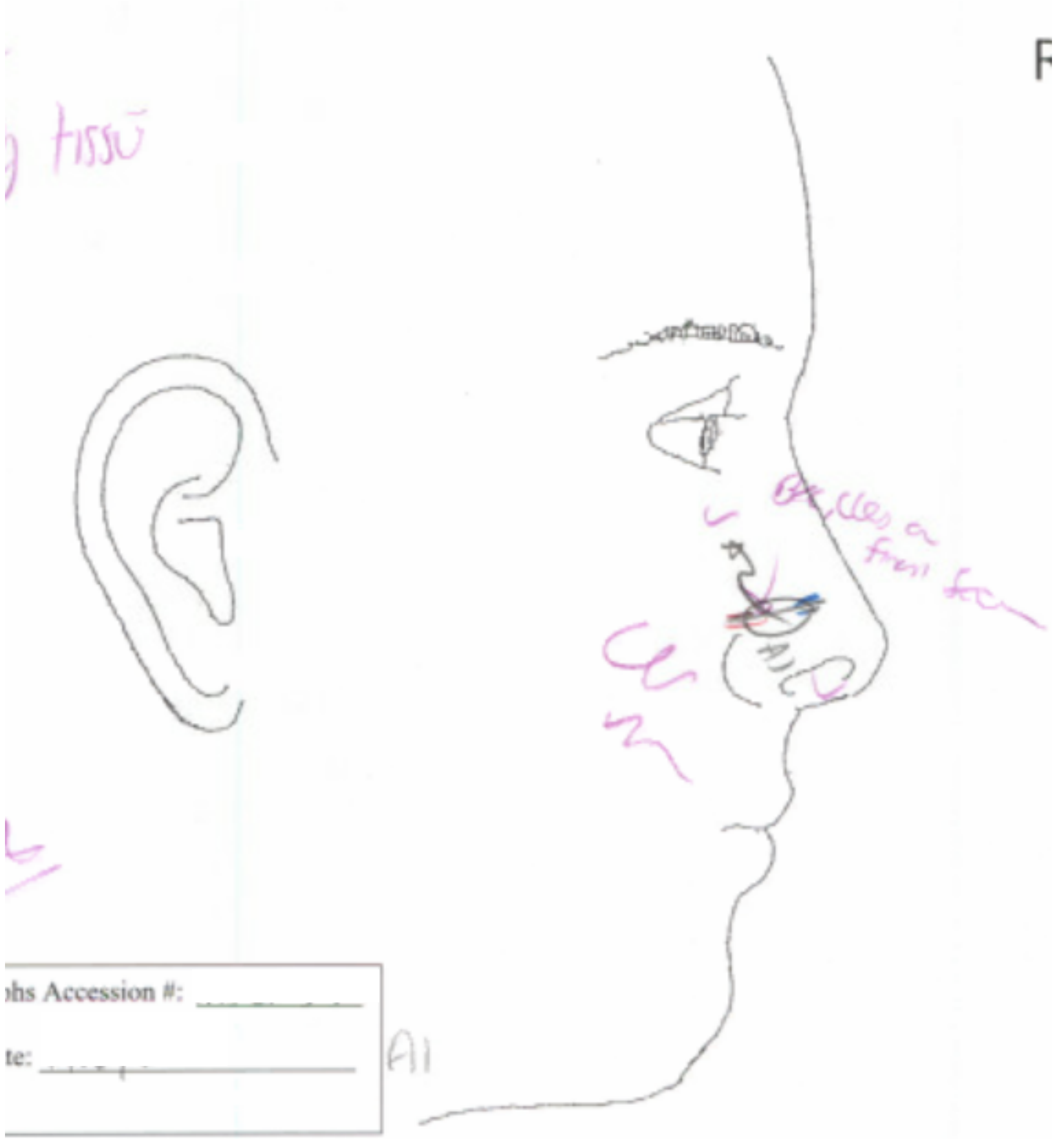
hs Accession #:
te:
A1

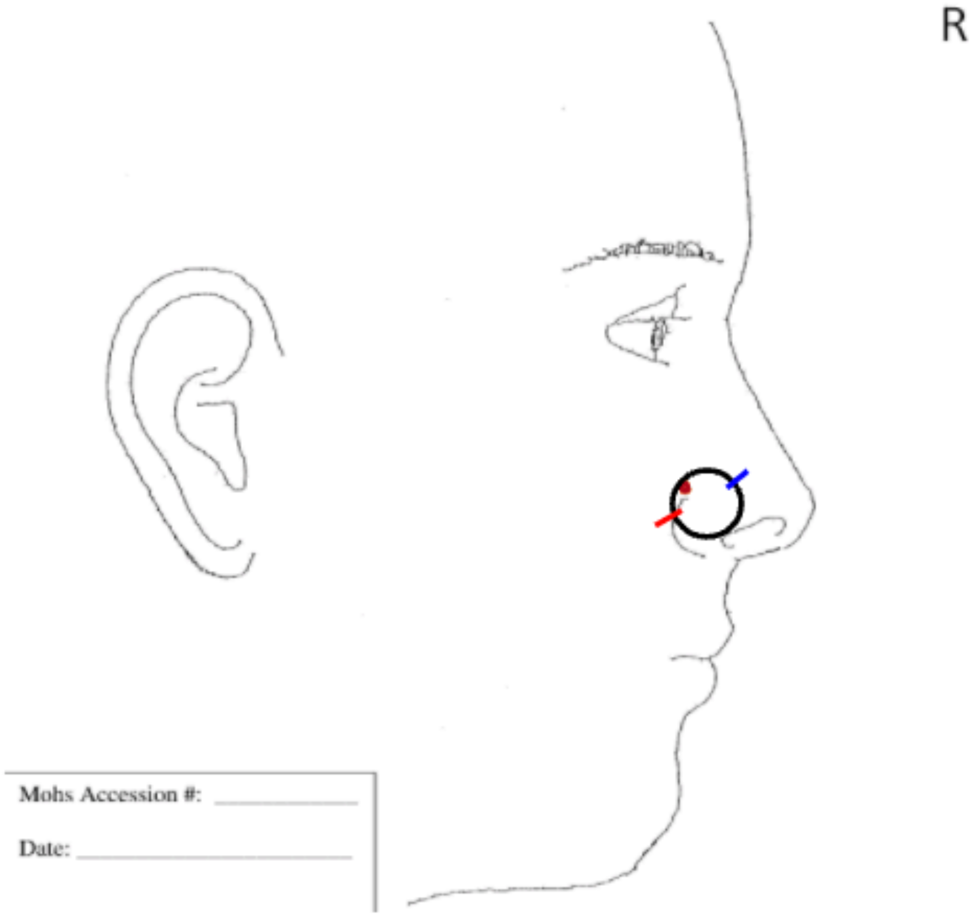
R
Mohs Accession #:
Date:

R

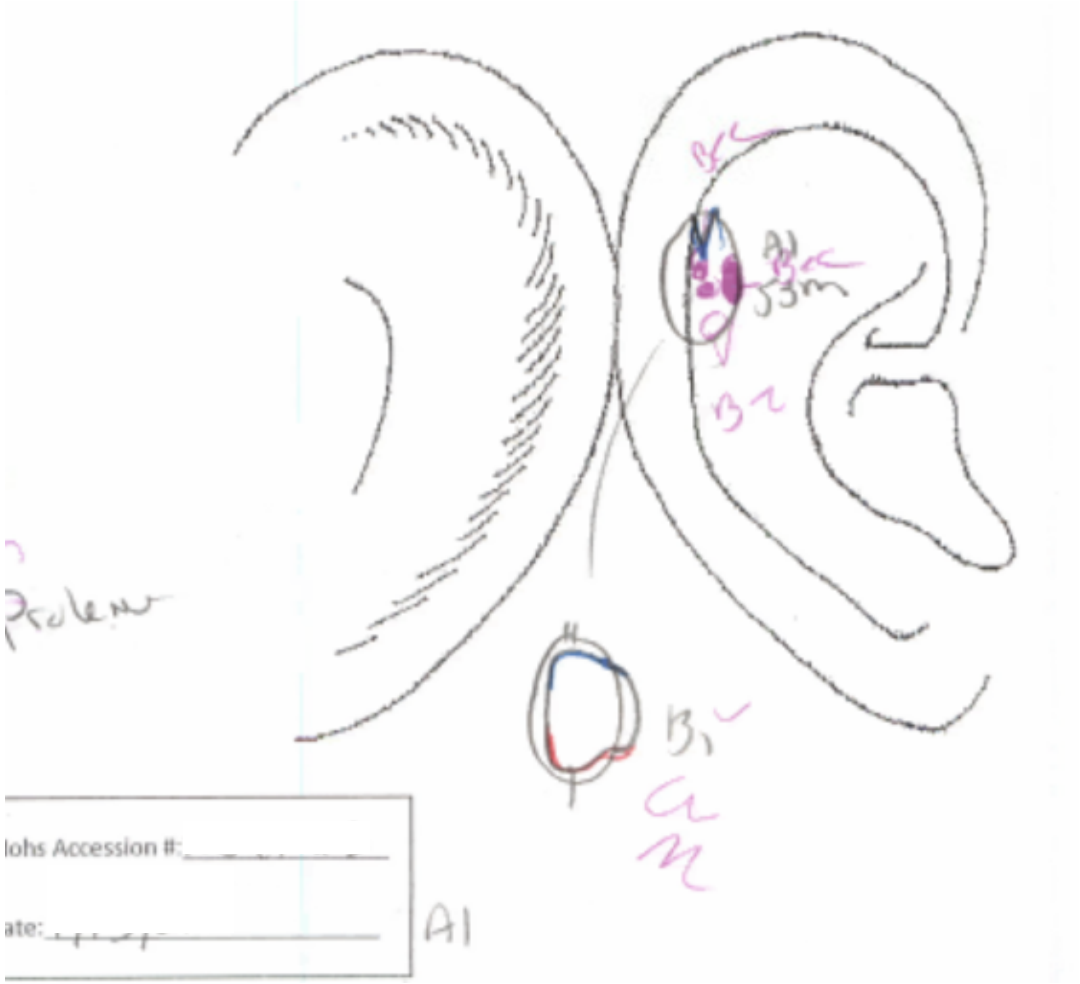

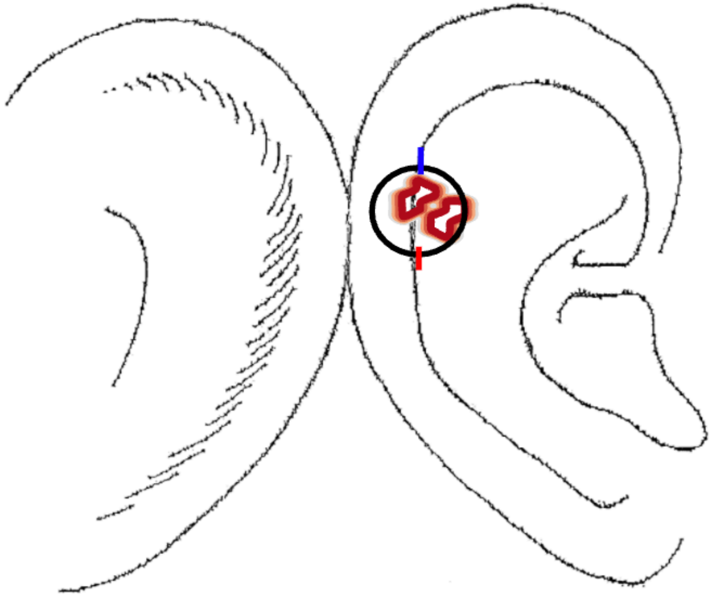

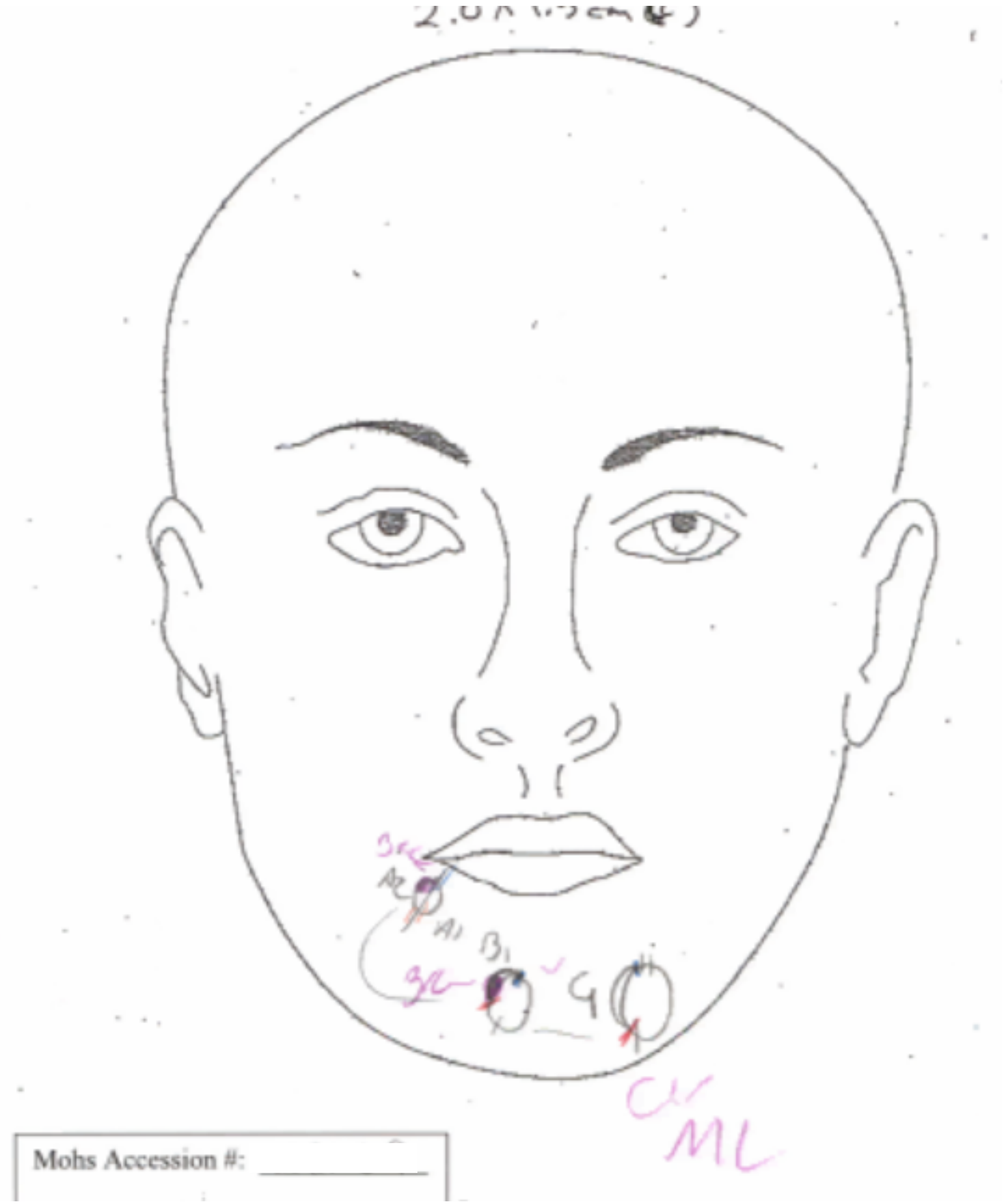

A

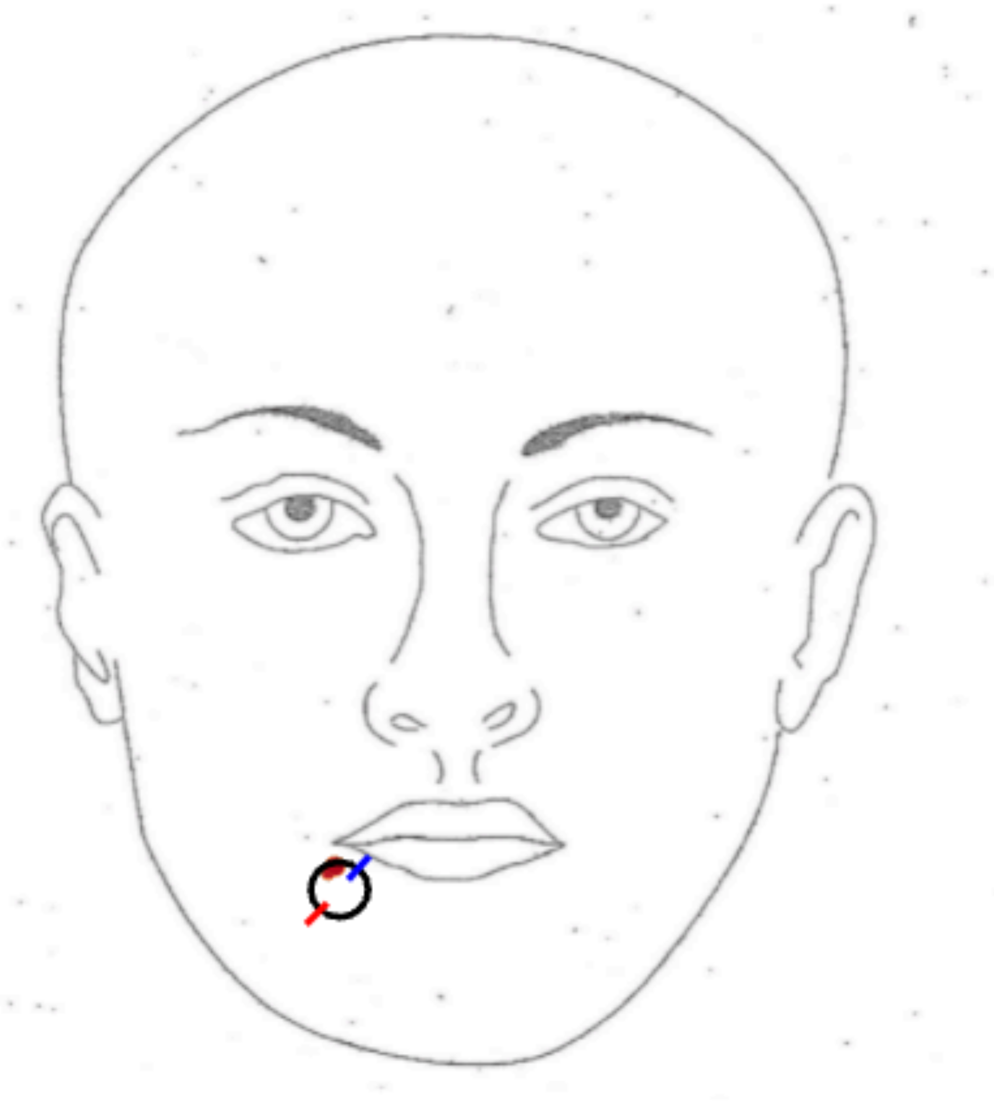

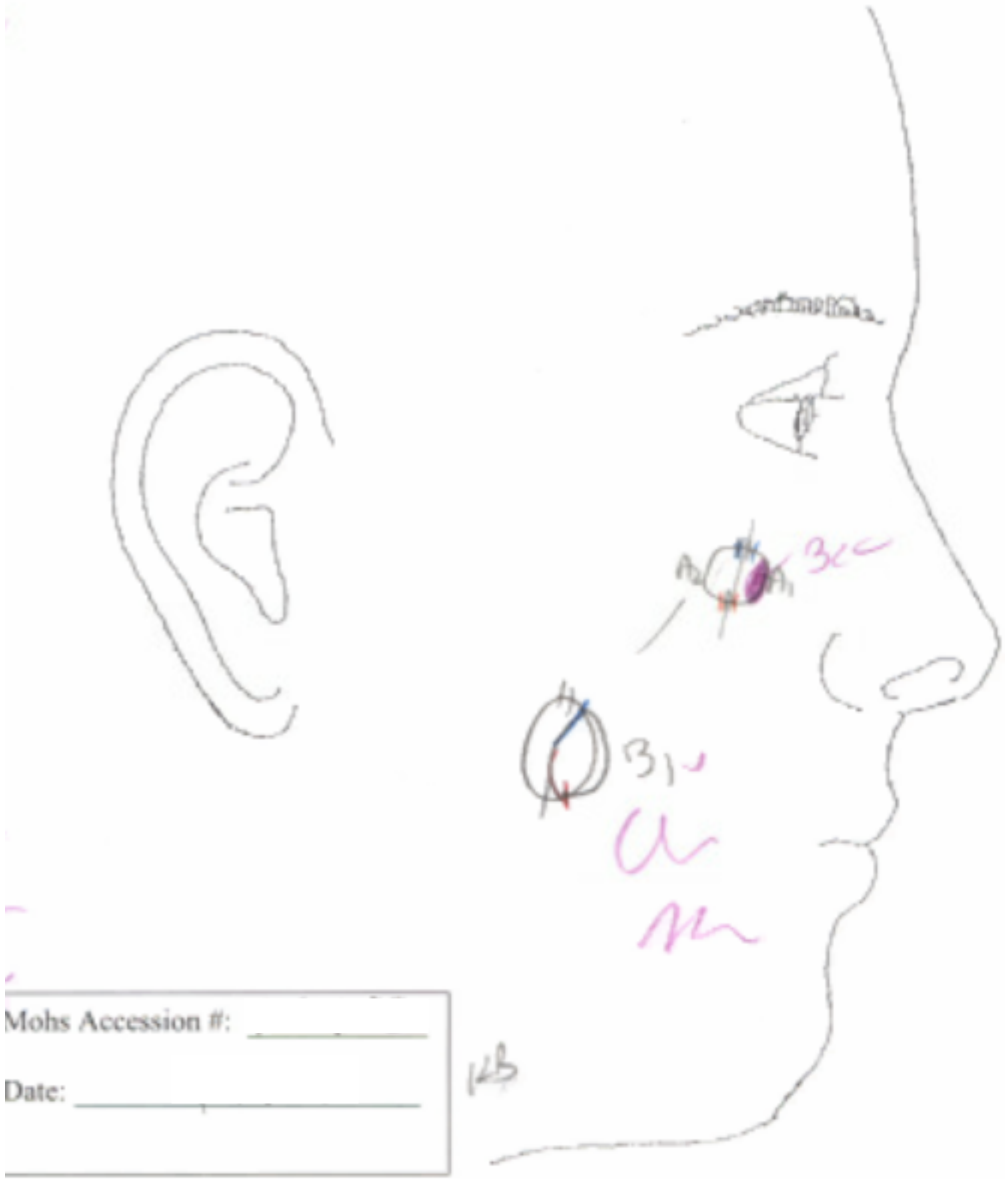
Mohs Accession #:
Date:

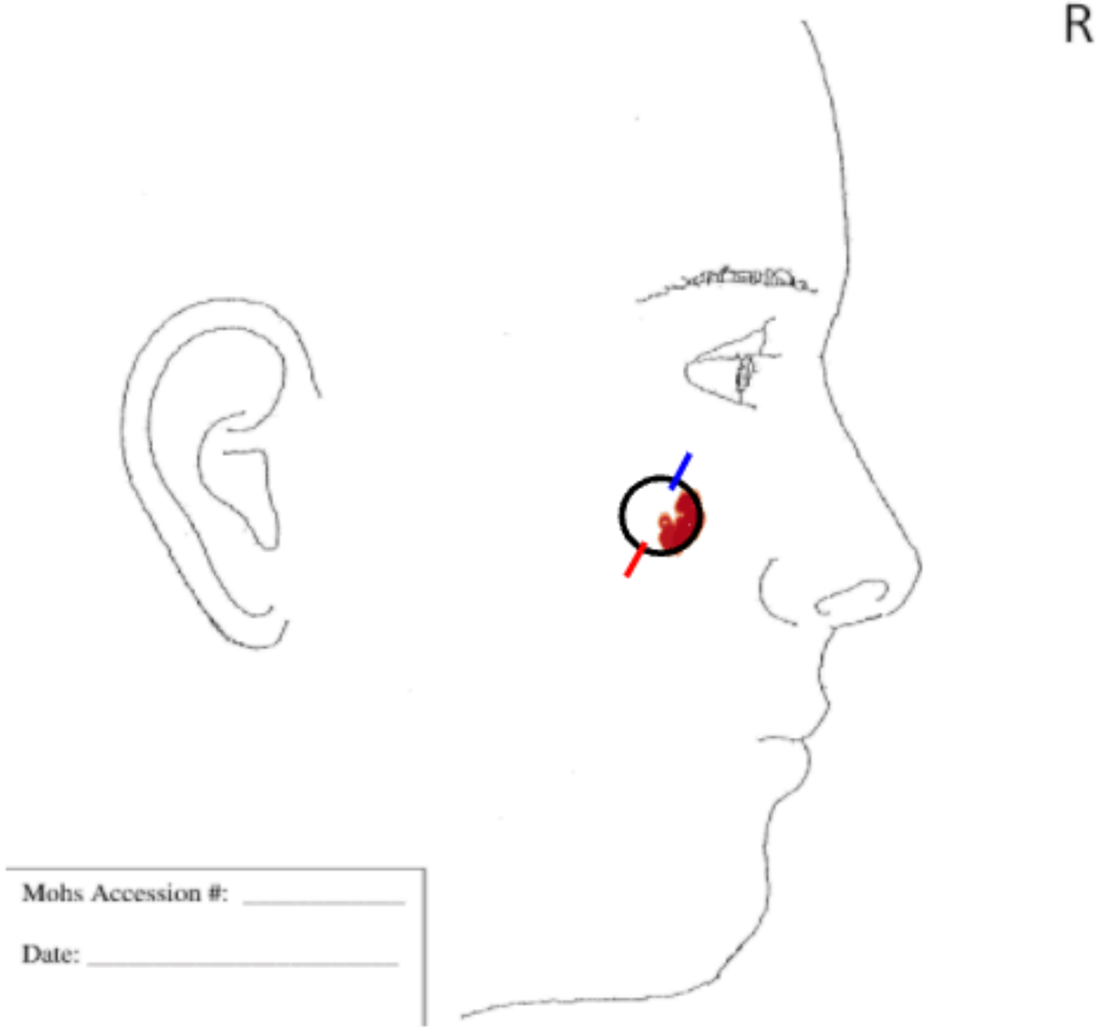
R
Mohs Accession #:
Date:

Tumor Type	Location (per path report)	Anatomic Area
BCC - nodular	(L) neck	N fa[illegible]
**Initial size (cm)**	**Final Defect (cm)**	**Room #**
1.1 x 0.9 cm	1.4 x 1.3 cm	30[illegible]

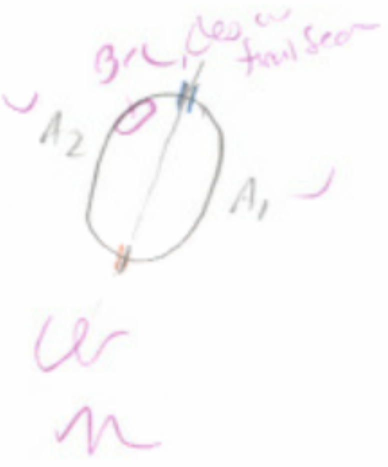

Mohs Accession #: ______________

Date: [illegible]

A1

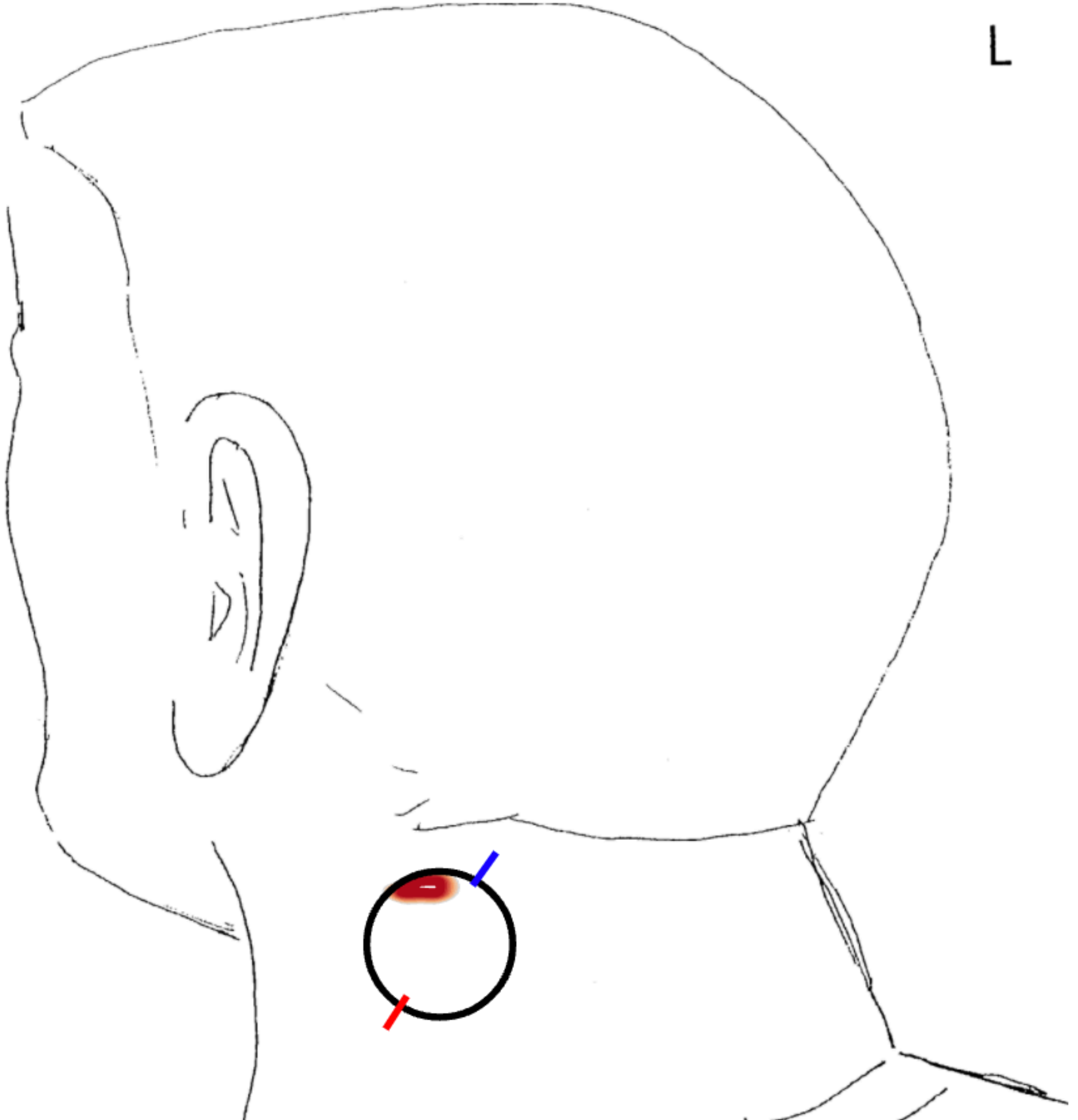

Mohs Accession #: ______________

Date: ______________________
