# Supplementary material for: Intraoperative margin assessment for basal cell carcinoma with deep learning and histologic tumor mapping to surgical site
Source: NPJ Precis Oncol. 2024 Jan 3;8:2. doi: 10.1038/s41698-023-00477-7 (PMC10764333; doi:10.1038/s41698-023-00477-7)
Supplement: Supplementary file 2 — Reporting Summary [file 41698_2023_477_MOESM2_ESM.pdf]

nature portfolio

Corresponding author(s): Joshua Levy

Last updated by author(s): 10/20/2023

Reporting Summary

Nature Portfolio wishes to improve the reproducibility of the work that we publish. This form provides structure for consistency and transparency in reporting. For further information on Nature Portfolio policies, see our Editorial Policies and the Editorial Policy Checklist.

Statistics

For all statistical analyses, confirm that the following items are present in the figure legend, table legend, main text, or Methods section.

n/a	Confirmed	
☐	☒	The exact sample size (n) for each experimental group/condition, given as a discrete number and unit of measurement
☐	☒	A statement on whether measurements were taken from distinct samples or whether the same sample was measured repeatedly
☐	☒	The statistical test(s) used AND whether they are one- or two-sided<br>*Only common tests should be described solely by name; describe more complex techniques in the Methods section.*
☐	☒	A description of all covariates tested
☐	☒	A description of any assumptions or corrections, such as tests of normality and adjustment for multiple comparisons
☐	☒	A full description of the statistical parameters including central tendency (e.g. means) or other basic estimates (e.g. regression coefficient) AND variation (e.g. standard deviation) or associated estimates of uncertainty (e.g. confidence intervals)
☐	☒	For null hypothesis testing, the test statistic (e.g. F, t, r) with confidence intervals, effect sizes, degrees of freedom and P value noted<br>*Give P values as exact values whenever suitable.*
☐	☒	For Bayesian analysis, information on the choice of priors and Markov chain Monte Carlo settings
☐	☒	For hierarchical and complex designs, identification of the appropriate level for tests and full reporting of outcomes
☐	☒	Estimates of effect sizes (e.g. Cohen's d, Pearson's r), indicating how they were calculated

Our web collection on statistics for biologists contains articles on many of the points above.

Software and code

Policy information about availability of computer code

Data collection	After Institutional Review Board approval, we assessed specimens from 194 patients undergoing tumor excision in the Mohs Micrographic Surgery (MMS) setting for the treatment basal cell carcinoma (BCC). Tissue from 16 patients (17 specimens) were used for tissue grossing algorithms, while tissue from the remaining 178 patients were used for histological assessment and tumor mapping algorithms. All specimens first underwent accessioning and gross measurement. For the tissue grossing algorithm, the gross specimen was placed on a turntable and imaged using low resolution video capture. The remaining cases underwent grossing, inking, processing, cryoembedding (frozen section), sectioning, and staining with hematoxylin and eosin (H&E). From these 178 cases, 351 slides corresponding to 1,065 serial sections and 1,537 tissue pieces were scanned at 20X resolution using the Leica Aperio AT2 scanner and stored as Whole Slide Images (WSI) in either SVS or TIFF file format.
Data analysis	ArcticAI is a publicly available package that can be installed using pip (pypi: arctic-ai) and is hosted on GitHub at the following URL: https://github.com/jlevy44/ArcticAI/. To ensure reproducible computing, ArcticAI is also available as a Docker/Singularity container, with installation instructions on the GitHub website. For readers who are interested in developing their own intraoperative margin assessment workflows, documentation for operating the application programming interface (API) and command line interface (CLI) can be found on our GitHub wiki (https://github.com/jlevy44/ArcticAI/wiki) and on our accompanying ReadtheDocs website (https://jlevy44.github.io/ArcticAI/). Please note that as the neural network models were developed at a single site– separate models will need to be trained at each institution and models trained for this manuscript can be made available upon reasonable request. A tutorial for training graph neural networks can be found at the following GitHub URL: https://github.com/jlevy44/WSI-GTFE. To learn more about the ArcticAI approach and how to operate it, we have provided a publicly available web application at the following URL: https://arcticai.demo.levylab.host.dartmouth.edu/. This application demonstrates 3D specimen grossing recommendations, histological examination, and mapping of histological results back to the surgical site for select cases. We have also included a video demonstrating operation of this web application at the following URL (Supplementary Figures 16-18): https://www.youtube.com/watch?v=I1Gfe6xI3Yg .

ArcticAI relies on the following Python packages: alphashape (v1.3.1), click (v8.1.3), dask (v2023.2.1), fire (v0.5.0), kornia (v0.6.10), pathflowai/pathpretrain (latest version), numpy (v1.24.2), pandas (v1.5.3), tifffile (v2021.11.2), opencv (v4.5.5.62), scikit-learn (v1.2.1), scikit-image (v0.18.3), pytorch (v1.13.1), torchvision (v0.14.1), torch-geometric (all related packages with versions compatible with pytorch), detectron2 (latest version), seaborn (v0.12.0), shapely (v2.0.1), matplotlib (v3.7.0), plotly (v5.17.0), dash (v2.14.0), toil (v5.12.0), instant-ngp (latest version), and POT (python optimal transport). Jupyter notebooks were used for custom prototyping, training and evaluation of the model results while ArcticAI was used for large scale inference across the test set slides. Default training/inference input arguments can be found in the GitHub repository. Credible intervals for concordance assessments between hand-drawn and digital tumor maps developed using R v4.1.

For manuscripts utilizing custom algorithms or software that are central to the research but not yet described in published literature, software must be made available to editors and reviewers. We strongly encourage code deposition in a community repository (e.g. GitHub). See the Nature Portfolio guidelines for submitting code & software for further information.

Data

Policy information about availability of data

All manuscripts must include a data availability statement. This statement should provide the following information, where applicable:

- Accession codes, unique identifiers, or web links for publicly available datasets
- A description of any restrictions on data availability
- For clinical datasets or third party data, please ensure that the statement adheres to our policy

The datasets presented in this article are not readily available because of participant privacy concerns. A subset data produced in the present study may be made available upon reasonable request to the authors. A subset of data is also readily accessible through our web application: https://arcticai.demo.levylab.host.dartmouth.edu/ . Requests to access the datasets should be directed to joshua.j.levy@dartmouth.edu.

Research involving human participants, their data, or biological material

Policy information about studies with human participants or human data. See also policy information about sex, gender (identity/presentation), and sexual orientation and race, ethnicity and racism.

Reporting on sex and gender	This study will represent analysis of biospecimens, and data collected from individuals with Basal Cell Carcinoma (BCC) currently undergoing surgical resection of tumor. Data will include medical histories abstracted from medical records, histological findings along with report of various BCC subtypes. The selection of females for the study reflects incidence rates of BCC in women in New Hampshire.
Reporting on race, ethnicity, or other socially relevant groupings	This study will represent analysis of biospecimens, and data collected from individuals with Basal Cell Carcinoma (BCC) currently undergoing surgical resection of tumor. Data includes medical histories abstracted from medical records, histological findings along with report of various BCC subtypes. The selection of minorities for the study reflects incidence rates of BCC for these groups, considering demographics statistics on race and ethnicity in the New Hampshire region.
Population characteristics	There will only be a few minority participants since New Hampshire is over 95% Caucasian and BCC more commonly occurs in a Caucasian population. The selection of females for the study reflects incidence rates of BCC in women in New Hampshire.
Recruitment	The proposed study for this application will consist of subjects undergoing Basal Carcinoma (BCC) tumor resection, where skin tissue from BCC tumor resection site will be collected prospectively in collaboration with a MOHS surgeon at Dartmouth Hitchcock Medical Center who has agreed to help with our grant aims. In this proposal, the existing data collection protocol does not require informed consent (IRB: STUDY00031766), which streamlines data collection.
Ethics oversight	Human Research Protection Program (IRB) of Dartmouth Hitchcock Medical Center. Consent for review of medical records, study questionnaires, and collection of the samples was waived due to retrospective review. The authors complied with all relevant ethical regulations including the Declaration of Helsinki. Human Research Protection Program (institutional review board, IRB) of Dartmouth Hitchcock Medical Center gave ethical approval for this work. All necessary patient/participant consent was obtained, including written consent, and the appropriate institutional forms have been archived, and that any patient/participant/sample identifiers included were not known to anyone (e.g., hospital staff, patients or participants themselves) outside the research group so cannot be used to identify individuals.

Note that full information on the approval of the study protocol must also be provided in the manuscript.

Field-specific reporting

Please select the one below that is the best fit for your research. If you are not sure, read the appropriate sections before making your selection.

☒ Life sciences ☐ Behavioural & social sciences ☐ Ecological, evolutionary & environmental sciences

For a reference copy of the document with all sections, see nature.com/documents/nr-reporting-summary-flat.pdf

Life sciences study design

All studies must disclose on these points even when the disclosure is negative.

Sample size	Sample size was based on data availability to calculate concordance and accuracy. Confidence intervals for the study findings were calculated

Sample size	using non-parametric bootstrapping and posterior distributions.
Data exclusions	No exclusion from selected cohort.
Replication	None to disclose
Randomization	None to disclose
Blinding	None to disclose

Reporting for specific materials, systems and methods

We require information from authors about some types of materials, experimental systems and methods used in many studies. Here, indicate whether each material, system or method listed is relevant to your study. If you are not sure if a list item applies to your research, read the appropriate section before selecting a response.

Materials & experimental systems

n/a	Involved in the study
☒	☐ Antibodies
☒	☐ Eukaryotic cell lines
☒	☐ Palaeontology and archaeology
☒	☐ Animals and other organisms
☒	☐ Clinical data
☒	☐ Dual use research of concern
☒	☐ Plants

Methods

n/a	Involved in the study
☒	☐ ChIP-seq
☒	☐ Flow cytometry
☒	☐ MRI-based neuroimaging
